# Quantum Chemical Study Aimed at Modeling Efficient Aza-BODIPY NIR Dyes: Molecular and Electronic Structure, Absorption, and Emission Spectra

**DOI:** 10.3390/molecules25225361

**Published:** 2020-11-17

**Authors:** Alexander E. Pogonin, Artyom Y. Shagurin, Maria A. Savenkova, Felix Yu. Telegin, Yuriy S. Marfin, Arthur S. Vashurin

**Affiliations:** 1 Department of Nanomaterials and Ceramic Technology, Ivanovo State University of Chemistry and Technology, Sheremetevsky av., 7, 153000 Ivanovo, Russia; mariasavenkova@bk.ru; 2 Department of Inorganic Chemistry, Ivanovo State University of Chemistry and Technology, Sheremetevsky av., 7, 153000 Ivanovo, Russia; Shagurin.A.Y@gmail.com (A.Y.S.); f.telegin@mail.ru (F.Y.T.); marfin@isuct.ru (Y.S.M.); vashurin@isuct.ru (A.S.V.)

**Keywords:** quantum chemical calculations, molecular structure, aza-BODIPY, intramolecular rotation, absorption spectra, vibronic spectra

## Abstract

A comprehensive study of the molecular structure of aza-BODIPY and its derivatives, obtained by introduction of one or more substituents, was carried out. We considered the changes in the characteristics of the electronic and geometric structure of the unsubstituted aza-BODIPY introducing the following substituents into the dipyrrin core; phenyl, 2-thiophenyl, 2-furanyl, 3-pyridinyl, 4-pyridinyl, 2-pyridinyl, and ethyl groups. The ground-state geometries of the unsubstituted Aza-BODIPY and 27 derivatives were computed at the PBE/6-31G(d) and CAM-B3LYP/6-31+G(d,p) levels of theory. The time-dependent density-functional theory (TDDFT) together with FC vibronic couplings was used to investigate their absorption and emission spectra.

## 1. Introduction

Optical molecular imaging is a powerful tool to gain a better understanding of biological phenomena and the mechanisms of action of therapeutic agents [1,2]. However, it has certain limitations for clinical applications due to the limited penetrability of visible light. The highest penetration depth can be achieved when the fluorophore absorbs and emits in a region of the electromagnetic spectrum where biological tissues exhibit less absorption and less autofluorescence (therapeutic window: 650−900 nm) [3]. In the search for such fluorescent probes, a series of 4,4′-difluoro-4-bora-3a,4a-diaza-s-indacene (abbreviated as BODIPY) dyes could be regarded as perspective candidates. BODIPY dyes are a fluorescent family with unique characteristics such as intense absorption, high quantum yields, tunable emission wavelength, superior stability, and pH insensitivity, which may survive different in vivo environments [4,5]. The most prevalent applications of BODIPY dyes are related to different aspects of intracellular imaging of various organelles. Aza-BODIPY is a class of heteroatom-containing BODIPY analogues with near-infrared (NIR) absorption. It allows them to benefit from low autofluorescence of biomolecules, a smaller scattering background, and the applicability of low-cost excitation sources [6].

Aza-BODIPY derivatives could be considered as promising compounds for the design of functional materials with predictable properties [7]. The possibility of implementation of a wide range of mechanisms for the fine tuning of the spectral properties of individual compounds, conjugates, and related materials highlights the need for a rational design of new structures [8,9].

The introduction of certain substituents into the parent compound allows aimed modification of its physicochemical properties. In this study, we focused on several aza-BODIPY dyes with red-shifted fluorescence and the potential for the environmental response via charge transfer [10], internal rotation [11], and aggregation causing mechanisms [8,12].

To predict various physicochemical properties of compounds, it is necessary to determine their molecular structure. A large number of works are devoted to the stereochemistry of various classes of compounds. There are experimental data obtained by X-ray diffraction (XRD) analysis [13], microwave spectroscopy [14], gas electron diffraction method [14], etc. Due to the development of computer technology, structural data obtained using quantum chemical (QC) calculations have become dramatically important. As the potential energy surface (PES) might have a large number of local minima, the study of the conformational multiformity of polyatomic molecules is often a difficult task [15,16]. At the same time, understanding the features of the molecular structure and the possibilities of low-energy doped changes, caused by light absorption and associative processes, can play a key role in the prediction of the mechanisms of directional changes in the optical properties of compounds for fine tuning and application in molecular sensorics [17,18].

There are many works dedicated to modeling new effective materials that have the potential for applied use, for example, as sensitizers for dye-sensitized solar cells (DSSC) [19]. In an ever-growing field of chemistry, it is important to understand which metrics are appropriate for which properties. To design efficient sensitizers the following information can be used; the type and shape of the highest occupied molecular orbital (HOMO) and lowest unoccupied molecular orbital (LUMO), and the energies of LUMO and HOMO relative to the conduction band of TiO_2_ and the redox couple [19]. To achieve the most efficient charge transfer the HOMO should be located on the donor moiety and the LUMO on the acceptor moiety [19]. Wherein, the HOMO–LUMO gap can be used to study the photooxidative resistance [20], kinetic stability [21], and electronic properties [19]. Still, the most important parameter, determined by the electronic structure of the compounds, is the position of the absorption and emission bands in the electronic spectra. The rational design of new structures for specific practical applications lies in the directional modification of the spectral band positions. At the same time, the number of fluorophores whose working wavelengths lie in the 400–600 nm region is large, in contrast to the compounds with spectral region close to the IR, which are necessary in medicine and molecular sensorics of biological systems. Taking that into account, the main objective of the screening process is to find the compound with the greatest bathochromic shift of absorption/emission compared to the unsubstituted aza-BODIPY.

**Figure 1:**
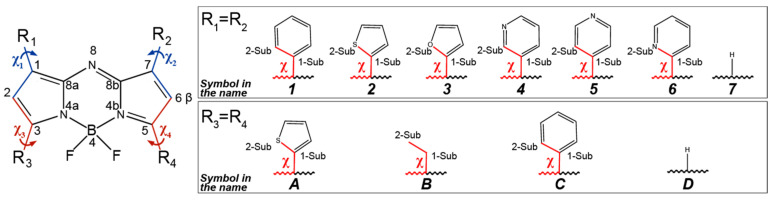
Atom numbering, structures, and naming scheme of the investigated molecules used throughout the paper. Color denotes dihedral angles C_2_-C_1_-C_1-Sub_-X_2-Sub_ (χ_1_), C_6_-C_7_-C_1-Sub_-X_2-Sub_ (χ_2_), C_2_-C_3_-C_1-Sub_-X_2-Sub_ (χ_3_), and C_6_-C_5_-C_1-Sub_-X_2-Sub_ (χ_4_). Molecules are named according to the symbols of substituents R_1_–R_4_: **A1**—1,7-diphenyl-3,5-di(2-thiophenyl)-aza-BODIPY, **A2**—1,3,5,7-tetra(2-thiophenyl)-aza-BODIPY, **A3**—1,7-di(2-furanyl)-3,5-di(2-thiophenyl)-aza-BODIPY, **A4**—1,7-di(3-pyridinyl)-3,5-di(2-thiophenyl)-aza-BODIPY, **A5**—1,7-di(4-pyridinyl)-3,5-di(2-thiophenyl)-aza-BODIPY, **A6**—1,7-di(2-pyridinyl)-3,5-di(2-thiophenyl)-aza-BODIPY, **A7**—3,5-di(2-thiophenyl)-aza-BODIPY, **B1**—1,7-diphenyl-3,5-diethyl-aza-BODIPY, **B2**—1,7-di(2-thiophenyl)-3,5-diethyl-aza-BODIPY, **B3**—1,7-di(2-furanyl)-3,5-diethyl-aza-BODIPY, **B4**—1,7-di(3-pyridinyl)-3,5-diethyl-aza-BODIPY, **B5**—1,7-di(4-pyridinyl)-3,5-diethyl-aza-BODIPY, **B6**—1,7-di(2-pyridinyl)-3,5-diethyl-aza-BODIPY, **B7**—3,5-diethyl-aza-BODIPY, **C1**—1,3,5,7-tetraphenyl-aza-BODIPY, **C2**—1,7-di(2-thiophenyl)-3,5-diphenyl-aza-BODIPY, **C3**—1,7-di(2-furanyl)-3,5-diphenyl-aza-BODIPY, **C4**—1,7-di(3-pyridinyl)-3,5-diphenyl-aza-BODIPY, **C5**—1,7-di(4-pyridinyl)-3,5-diphenyl-aza-BODIPY, **C6**—1,7-di(2-pyridinyl)-3,5-diphenyl-aza-BODIPY, **C7**—3,5-diphenyl-aza-BODIPY, **D1**—1,7-diphenyl-aza-BODIPY, **D2**—1,7-di(2-thiophenyl)-aza-BODIPY, **D3**—1,7-di(2-furanyl)-aza-BODIPY, **D4**—1,7-di(3-pyridinyl)-aza-BODIPY, **D5**—1,7-di(4-pyridinyl)-aza-BODIPY, **D6**—1,7-di(2-pyridinyl)-aza-BODIPY, **D7**—aza-BODIPY.

In this paper, we studied the effect of introducing the following substituents into the different positions; phenyl, 2-thiophenyl, 2-furanyl, 3-pyridinyl, 4-pyridinyl, 2-pyridinyl, and ethyl groups (R_1_–R_4_, Figure 1).

The molecule of the original aza-BODIPY possesses C_2v_ symmetry [22]. Placing polyatomic substituents in the C_1_/C_3_/C_5_/C_7_ positions of aza-BODIPY leads to distortion of the planar structure of the heterocycle. In case of aza-BODIPY derivatives, it is necessary to study the conformational multiformity of a molecule in order to find an appropriate minimum on PES. In addition, the study of rotations of cyclic appendages is relevant as the dihedral angle between dipyrrin and substituent group will have a great influence on the HOMO-LUMO energy gap and absorption spectra [23]. Therefore, the first aim of the present contribution is to study different conformers of aza-BODIPY derivatives formed as a result of the rotation of substituent groups R_1_–R_4_ (χ_1_, χ_2_, χ_3_, χ_4_).

**Figure 2:**
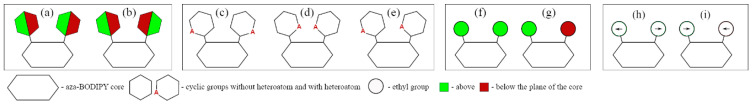
Conformer models of aza-BODIPY derivatives with substituent groups at C_1_, C_7_ (or C_3_, C_5_) positions. Depiction of conformational multiformity coupled to different mutual orientations of neighboring cyclic groups: (**a**) structure of C_2_ symmetry; (**b**) structure of C_s_ symmetry; to different arrangement of heteroatoms with respect to the center of the molecule: (**c**) heteroatoms in cyclic groups are oriented by “outer” direction with respect to center of molecule; (**d**) heteroatoms in cyclic groups are directed by “inner” direction with respect to center of molecule; (**e**) one heteroatom in one cyclic group is directed by “inner” direction, another heteroatom in another group—by “outer” direction; different orientations of ethyl groups: (**f**) model of C_s_ symmetry according to which ethyl groups are oriented in the same direction relating to aza-BODIPY core; (**g**) model of C_2_ symmetry according to which ethyl groups are oriented in the opposite direction relating to aza-BODIPY core; (**h**) two ethyl groups are in plane of molecular core and the groups are oriented by “outer” direction with respect to center of molecule; (**i**) two ethyl groups are in plane of molecular core with direction of the ethyl group toward each other.

**Figure 3:**
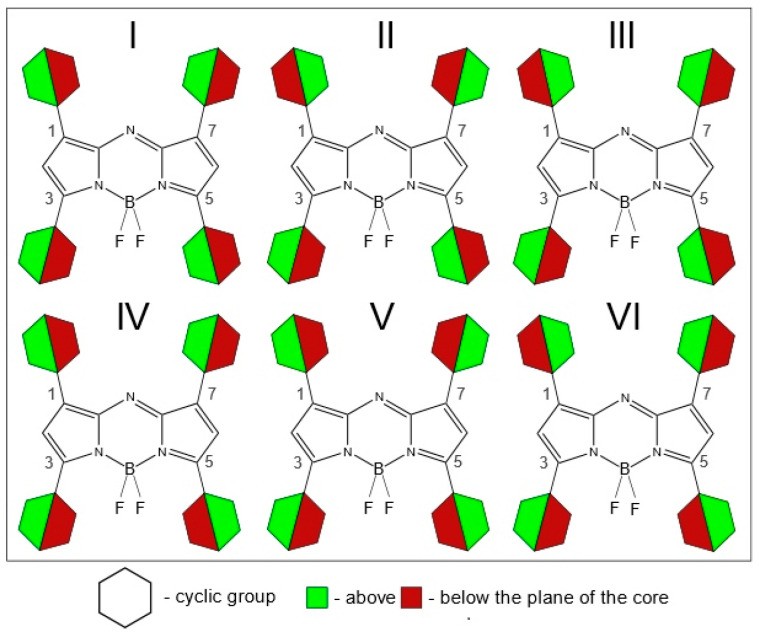
Conformation models of aza-BODIPY analogues substituted by cyclic groups in positions 1, 3, 5, and 7. Depiction of conformational multiformity coupled to different mutual orientations of cyclic groups: **I**—all four cyclic substituents are quasi-parallel to each other; **II**—neighboring cyclic groups R_1_ and R_2_, R_3_ and R_4_ are quasi-parallel to each other in pairs, however R_1_ and R_3_, R_2_ and R_4_ are not quasi-parallel to each other in pairs; **III**—neighboring cyclic groups R_3_ and R_4_ are quasi-parallel, R_1_ and R_2_ are mirrored relative to each other; **IV**—neighboring cyclic groups R_3_ and R_4_ are mirrored relative to each other, R_1_ and R_2_ are quasi-parallel; **V**—neighboring cyclic groups R_1_ and R_2_, R_3_ and R_4_ are mirrored relative to each other in pairs, R_1_ and R_3_, R_2_ and R_4_ are quasi-parallel to each other in pairs; **VI**—neighboring cyclic groups R_1_ and R_2_ are mirrored relative to each other in pairs, R_1_ and R_3_, R_2_ and R_4_ are not quasi-parallel to each other in pairs.

In the case of molecules with two substituents (**A7**, **B7**, **C7**, **D1**–**D6**), the main reasons for the conformational diversity are different mutual orientations of neighboring (R_1_ and R_2_ or R_3_ and R_4_) cyclic and ethyl groups (Figure 2). In case of the presence of four cyclic substituents, the number of possible conformers grows. Figure 3 shows a graphical representation of possible conformer multiformity caused by the mutual arrangement of not only neighboring substituents but also long-distance substituents. Figure 3 does not take into consideration possible locations of asymmetric cyclic groups with a heteroatom (Figure 2c–e).

The efficient strategy for conformers searching is to use meta-dynamical simulations. Meta-dynamical simulations work by applying a biasing potential (here it is based on root mean square deviation (RMSD) between the current and reference structure in Cartesian coordinates) that ensures that the collective variables (i.e., all atom coordinates) do not converge to the same local minima often and can, therefore, find other, sometimes unexpected structures. More theoretical explanation is available in [24]. The semiempirical method used was shown [25,26] to be in good agreement to density functional theory (DFT) when it comes to the qualitative results, i.e., which conformer is the lowest in energy. Thereby, meta-dynamical calculations were used as the first step in the search for the most energetically favorable conformers.

In order to learn more about the structural implications of different substituents and to reconfirm our conformational findings at the higher level, we have examined the process of rotation of substituent groups around C-C bonds. To minimize computer time, we decided to analyze conformers of **A1**–**A7**, **B1**–**B7**, **C1**–**C7**, and **D1**–**D7** in several steps:

1. To study molecules obtained by introducing one substituent (1–6) into the aza-BODIPY (**D7**) in position C_1_;
2. To study molecules **D1**–**D6** obtained by introducing two substituents (1–6) into the aza-BODIPY (**D7**) in positions C_1_/C_7_;
3. To study molecules obtained by introducing one substituent (A–C) into the aza-BODIPY (**D7**) in position C_3_;
4. To study molecules **A7**, **B7**, and **C7** obtained by introducing two substituents (A–C) into the aza-BODIPY (**D7**) in positions C_3_/C_5_;
5. Based on the analysis (steps No. 2 and 4), to select the appropriate conformers of **A1**–**A6**, **B1**–**B6**, and **C1**–**C6** for further investigation. Generally this step uses the assumption that the substituents in positions C_1_/C_7_ only slightly affect the rotations of the groups located in positions C_3_/C_5_ (and vice versa).

Due to the need for a large number of calculations in steps 1–4, we selected a combination of PBE functional and 6-31G* basis set. The choice of the PBE functional was made taking into account the small amount of CPU time needed for a single point calculation [27]. It has been shown [28] that the 6-31G* basis set is enough to estimate the geometries of the aza-BODIPY dyes correctly.

The second task of this work is to study the simulated electronic absorption and emission spectra. QC calculations of molecules in the solvated state (solvent is CH_2_Cl_2_) were performed using CAM-B3LYP functional [29] and 6-31+G(d,p) basis set in the framework of conductor-like polarizable continuum model (CPCM) [30,31]. Electronic absorption spectra of aza-BODIPY derivatives were modeled on the time-dependent density-functional theory (TDDFT) calculation results. CAM-B3LYP was chosen for its good performance for both ground and excited electronic state geometries [32]. The choice of CH_2_Cl_2_ as a solvent was made for easy comparison to the experimental data due to its prevalence in the aza-BODIPY synthesis procedure.

For calculating vibrationally-resolved absorption and emission spectra we decided to use the 6-31G(d) basis set to minimize the computer time.

## 2. Results and Discussion

### 2.1. Searching the Most Energetically Favorable Conformers: Meta-Dynamical Simulations

First, energetically favorable conformers for each molecule are shown in Figures S1–S4 in the Supplementary Materials. For **A1**–**A7**, the algorithm predicts the out-facing position (Figure 2c) of 2-thiophenyl substituents in positions C_3_/C_5_ for the lowest conformers. It should be noted the advantage of five-membered rings—because of the difference in ring size, all of them have a larger distance between C_2_/C_6_ and C_2-sub_-H hydrogens in the substituent allowing for a smaller dihedral angle.

For **B1**–**B7**, the lowest energy conformation corresponds to the in-plane or “side-facing” position of ethyl groups (R_3_, R_4_). The orientation of the R_1_,R_2_-substituents is the same as in the “A-series” counterparts. There is a sharp increase in the total amount of unique PES minima for each molecule compared to the aryl substituted dyes. Some of those states are quite low-lying. Those conformers are apparently thermally accessible due to the low barrier of rotation for ethyl groups.

For **C1**–**C7**, the algorithm predicts unusual mirror-symmetric orientation (Figure 2b) of phenyl rings (R_3_, R_4_) in the main conformer.

### 2.2. Internal Rotation of One Cyclic Substituent around Bonds C-C

**Figure 4:**
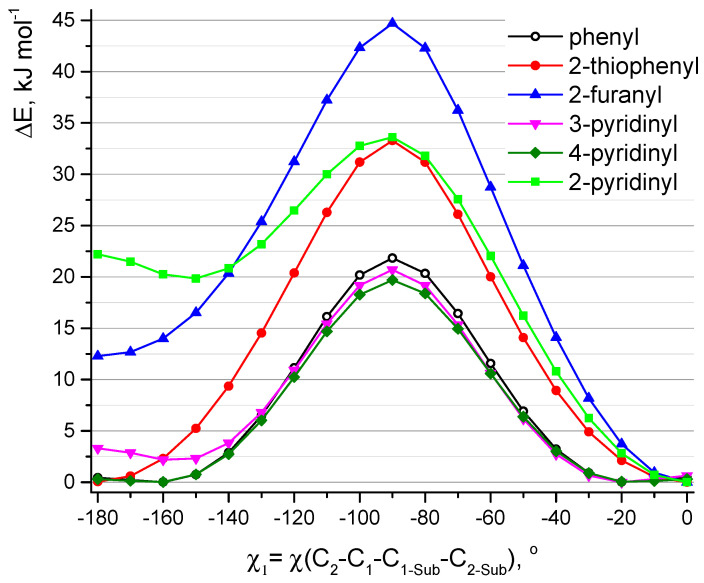
Relaxed potential energy function (PBE/6-31G(d)) of internal rotation of one group R_1_ in the derivatives of aza-BODIPY molecules around the C_1_-C_1_-_Sub_ bond. Positions C_3_, C_5_, C_7_ (Figure 1) in these molecules are occupied by hydrogen atoms.

At the first step, QC relaxed PES scan calculations (PBE/6-31G(d)) of aza-BODIPY derivatives with only one substituent R_1_ were carried out. Hydrogen atoms in these objects are in positions C_3_, C_5_, C_7_ (Figure 1). Rotation barriers of these groups increase in the sequence: 4-pyridinyl ≈ 3-pyridinyl ≈ phenyl < 2-thiophenyl ≈ 2-pyridinyl < 2-furanyl (Figure 4). 2-Furanyl, 2-thiophenyl, and 2-pyridinyl groups are located in the plane of the molecule. Phenyl, 4-pyridinyl, and 3-pyridinyl groups are not located in the plane of the cycle (χ_1_ = ∼18°), and therefore lead to a weak distortion of the aza-BODIPY skeleton. This is apparently explained by the steric repulsion associated with a short distance between H_2_ and H_2-Sub_: leaving the plane of the cycle leads to the distance r_e_(H_2_···H_2-Sub_) increase by ∼0.11 Å for phenyl, 4-pyridinyl, 3-pyridinyl (2.173 → 2.283 Å, 2.217 → 2.315 Å, 2.199 → 2.333 Å, respectively). The location (Figure 2) of substituents, where a heteroatom is oriented in the “outer” direction (χ_1_ ≈ 0°) with respect to the center of the molecule, is energetically favorable for 2-furanyl, 3-pyridinyl, 2-pyridinyl groups.

**Figure 5:**
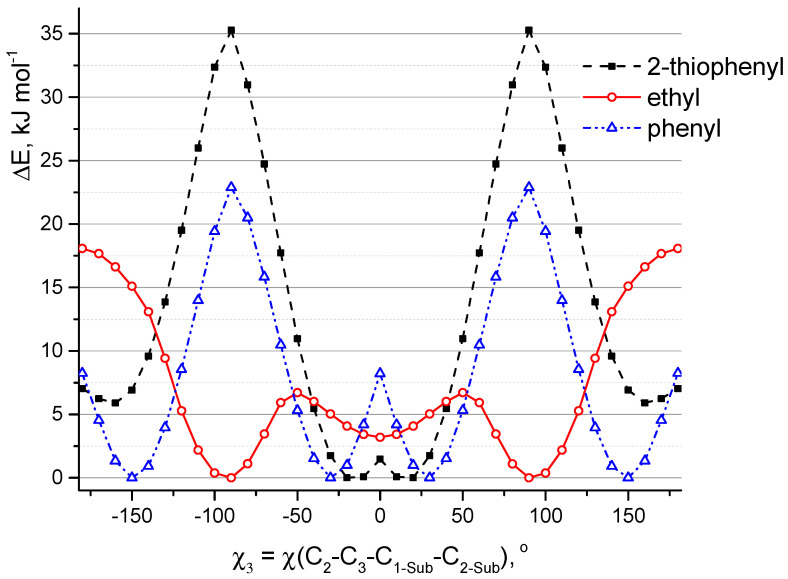
Relaxed potential energy function (PBE/6-31G(d)) of internal rotation of one group R_3_ in the derivatives of aza-BODIPY molecules around the C_3_-C_1-Sub_ bond. Positions C_1_, C_5_, and C_7_ (Figure 1) in these molecules are occupied by hydrogen atoms.

Similarly, QC calculations of aza-BODIPY derivatives with only one substituent R_3_ at position C_3_ were carried out (Figure 5). Positions C_1_, C_5_, and C_7_ in these molecules are occupied by hydrogen atoms. In addition to phenyl, 2-thiophenyl also moves out of the plane of the heterocycle (χ_3_ = ∼25°). The location of these groups in the plane of the molecule corresponds to the saddle point on PES. Substituents leave the plane via rotation as a result of the formation of a hydrogen bond between fluorine and hydrogen atoms. For the model in which sulfur atom of 2-thiophenyl is directed towards fluorine atoms, the planar structure is also not preferable. Apparently, this is due to the steric repulsion associated with a short distance r(H_2_···H_2-Sub_) = 2.192 Å (in planar conformation) and the presence of interaction between sulfur and fluorine atoms: according to QTAIM (Quantum Theory of Atoms in Molecules) analysis of non-flat conformation the value of electron density distribution function ρ(r) in appropriate bond critical point (BCP) is 0.015 and ∇ρ(r) is 0.057.

Ethyl group is found to be perpendicular to the plane of the heterocycle. The potential function of C_2_H_5_-rotation has three maxima corresponding to the values of the torsion angle χ_3_ equal to ±50° and 180° (Figure 5). The planar structure, in which ethyl group is bent toward the fluorine atoms (χ_3_ = 180°), corresponds to a saddle point with relative energy ΔE = 18.1 kJ mol^−1^. The structure characterized by the location of the ethyl group in the plane of the cycle (χ_3_ = 0°) corresponds to a shallow minimum. This goes against meta-dynamical simulations at semi-empirical level (Section 2.1, Figure S2). In this regard, additional calculations CAM-B3LYP/6-31+G(d,p) (for isolated molecule and for the solvated state) were carried out. According to the different calculations (PBE/6-31G(d) and CAM-B3LYP/6-31+G(d,p) in the framework of PCM and without PCM) the low barrier height to internal rotation of the ethyl group ranges from 1 to 7 kJ mol^−1^ and energy difference between conformers with perpendicular and planar location of ethyl group ranges from 0.3 to 4 kJ mol^−1^, respectively. Apparently, calculations with 6-31G(d) overestimate the barrier of ethyl rotation, in addition, according to these calculations, the perpendicular arrangement of ethyl groups is more energetically favorable. Despite this, the subsequent calculations (PCM/CAM-B3LYP/6-31+G(d,p)) of the **B1**–**B6** molecules showed that the structure with the arrangement of ethyl groups in the plane of the molecular skeleton is energetically more favorable by ∼1 kJ mol^−1^.

### 2.3. Rotations of Two Cyclic Substituent Groups at Positions 1,7 in **D1**–**D6** Molecules

Adding a second substituent to the aza-BODIPY core predictably increases the non-planarity of the molecule and leads to a higher movement of groups from the heterocycle plane. 2-Thiophenyl, 2-furanyl, and 2-pyridinyl groups in **D2**, **D3**, and **D6** molecules are located with χ_1_ = ∼11°. The rotation value of phenyl, 3-pyridinyl, and 4-pyridinyl groups in **D1**, **D4**, and **D5** molecules increases by ~5° compared to molecules with one substituent group (Table S1).

Even when two substituent groups are inserted into the molecule **D7**, structural studies are significantly complicated by the need to study the conformational multiformity. Conformational multiformity of derivatives **D1**–**D6** is caused for two reasons: (1) different arrangement of heteroatoms with respect to the center of the molecule (Figure 2c–e), and (2) different mutual orientations of neighboring substituent groups relating to each other (cyclic substituents are located outside the plane of the molecule due to their rotation) (Figure 2a,b).

**Figure 6:**
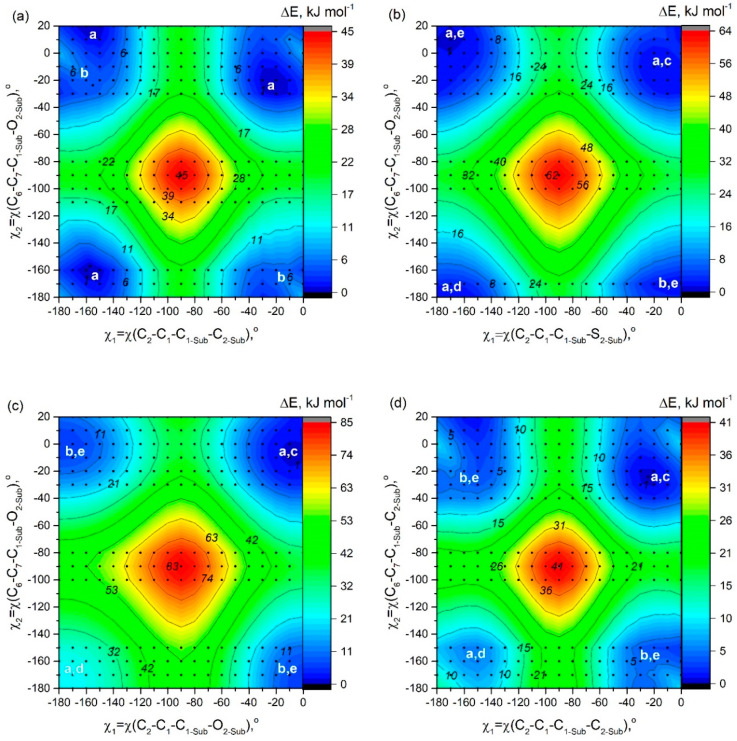

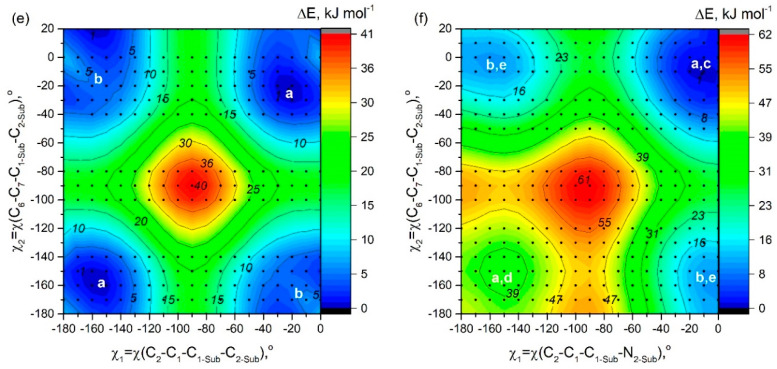
Potential energy surface (PES) obtained at the PBE/6-31G(d) level of theory showing potential energy as a function of rotations of two substituents (R_1_ and R_2_) around the bonds C_1_-C_1-Sub_ and C_7_-C_1-Sub_: (**a**) phenyl groups in molecule **D1**; (**b**) 2-thiophenyl groups in molecule **D2**; (**c**) 2-furanyl groups in molecule **D3**; (**d**) 3-pyridinyl groups in molecule **D4**; (**e**) 4-pyridinyl groups in molecule **D5**; (**f**) 2-pyridinyl groups in molecule **D6**. Black circles indicate the calculations performed for the corresponding values of the angles. White letters indicate conformations which are described in Figure 2.

In order to study possible conformers and barriers to synchronous internal rotations of two substituent groups, we performed PES scan using PBE/6-31G(d) calculations. As shown in Figure 6, in cases of **D1** and **D3**–**D6** molecules, points of lowest energy correspond to the area with χ_1_ = χ_2_ = [−25°; −7°]. It matches the structure of C_2_ symmetry according to which heteroatoms in substituents R_1_, R_2_ are oriented in an “outer” direction and neighboring cyclic groups are quasi-parallel (Figure 2). The mirror-symmetric structure of C_s_ symmetry with χ_1_ = −χ_2_ = ∼−13° is slightly higher by ∼0.8–4.5 kJ mol^−1^ (0.8 kJ mol^−1^—for **D3**; 4.4 kJ mol^−1^—for **D4**) and corresponds to a saddle point on the PES. In case of **D2** the structure with χ_1_ = −169.7° and χ_2_ = 11.0° (Figure 2a,e) possesses the lowest energy, however, the energy of conformer of C_2_ symmetry (Figure 2a,c) is only slightly higher by 0.4 kJ mol^−1^.

Barrier of internal rotation increases in the following order; **D5** (∼19 kJ mol^−1^) < **D4** (∼20 kJ mol^−1^) < **D1** (∼21 kJ mol^−1^) < **D6** (∼28 kJ mol^−1^) < **D2** (∼29 kJ mol^−1^) < **D3** (∼39 kJ mol^−1^). This order agrees with the information presented in Figure 2 and is the reverse of the dihedral angle order—**D3** has lowest χ_1_/χ_2_ and **D5** has the highest. This seems to be caused by the additional conjugation between R_1_, R_2_, and aza-BODIPY core needs to be broken during rotation, which takes a lot of energy. One of the more relevant areas of fluorophore research is viscosity measurement and molecular rotors. 8-Substituted BODIPY dyes are already a staple of such research, and maybe our aza-BODIPY dyes could also become useful tools.

### 2.4. Rotation of Two Substituent Groups at Positions 3,5 in Molecules **A7**, **B7**, and **C7**

**Figure 7:**
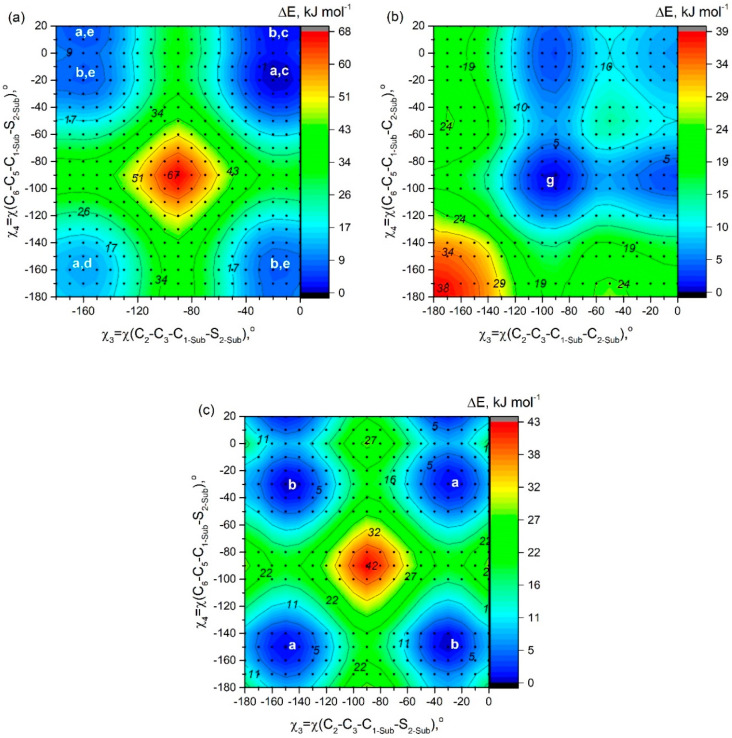
PES obtained at the PBE/6-31G* level of theory showing potential energy as a function of rotations of two substituents (R_3_, R_4_) around the bonds C_3_-C_1-Sub_ and C_5_-C_1-Sub_: (**a**) 2-thiophenyl groups in molecule A7, (**b**) ethyl groups in molecule B7, and (**c**) phenyl groups in molecule **C7**. Black circles indicate the calculations performed for the corresponding values of the angles. White letters indicate conformations which are described in the Figure 2.

The PES shown in Figure 7 has been constructed to study conformational multiformity of **A7**, **B7**, and **C7** coupled to rotations of 2-thiophenyl, ethyl, and phenyl groups. For **A7**, the minimum of potential energy corresponds to the structure characterized by quasi-parallel location of thiophene groups (Figure 2a) and an “outer” direction of sulfur atoms in substituents R_3_ and R_4_ (Figure 2c). The structure of C_s_ symmetry (Figure 2b,c) with another location of thiophene group is characterized by relative energy of 1.5 kJ mol^−1^ and a very small barrier of no more than 3 kJ mol^−1^—a fairly low-lying thermally accessible conformer.

In contradiction to **A7**, for **C7** QC PES scan calculations match the mirror-symmetric equilibrium structure of C_s_ symmetry predicted by meta-dynamical simulations: one fluorine atom forms hydrogen bonds with two phenyl rings -C_6_H_5_···F···H_5_C_6_-. The structure (C_2_ symmetry) with quasi-parallel location of phenyl groups is higher in energy only by 0.7 kJ mol^−1^. Quasi-parallel arrangement of substituent groups leads to the formation of two separate contacts F···H_5_C_6_- with the participation of two fluorine atoms and two phenyl groups. The barrier of internal rotation for phenyl groups (**C7**) is about 8 kJ mol^−1^.

In case of **B7**, the position of the minima corresponds to almost perpendicular orientation of the ethyl groups to the plane of the aza-BODIPY cycle. However, as described in Section 2.2, the use CAM-B3LYP/6-31+G(d,p) calculations led to another conclusion—a conformer with ethyl groups in the plane of the molecule is energetically more favorable.

### 2.5. Molecules of Tetra-Substituted Aza-BODIPYs: Conformational Multiformity and Molecular Structure

It is important to mention that a typical RMSD for the relative energies of conformers for many DFT functionals is well above 1 kJ mol^−1^: RMSD is equal to 0.61 kcal·mol^−1^ for PBE calculations of relative energies of alkanes [33]. This can lead to difficulties in changing from an initial calculation methodology to a final one. The results of calculations PBE/6-31G* and CAM-B3LYP/6-31+G(d,p) have several differences in the determination of the most favorable conformers (see for example, Table S6), as well as in the determination of the type of critical points on the PES. The mirror-symmetric arrangements of phenyl rings (Figure 2b) in **A1**, **B1**, **C1**, and **D1** correspond to saddle points on PES by PBE/6-31G(d), whereas such structures correspond to minimum on PES by CAM-B3LYP/6-31+G(d,p). Besides, according to CAM-B3LYP calculations of **A1** structures conformer V of C_s_ symmetry is energetically equal to conformer I. These differences are principal, but at the same time it should be considered insignificant for subsequent spectrum modeling. For instance, the wavelengths, corresponding to transitions to the first 25 lowest excited states, calculated using structures of conformer I and V of **C1** molecule, differ by no more than 4 nm (the average difference is 1 nm).

For further investigations at the level of the theory of CAM-B3LYP/6-31+G(d,p), conformers were selected and tested in accordance with the mentioned above molecular structure analysis of **A7**, **B7**, **C7**, and **D1**–**D6**. The conformer I (Figure 3) was chosen for a further study of the molecules **A1**–**A6**: all four cyclic substituents are quasi-parallel to each other. According to CAM-B3LYP/6-31+G(d,p) calculations the energy differences between conformers I–II are 5–10 kJ mol^−1^ for **A1**–**A6** (Table S6). It should be noted that for **A1**–**A6**, **B1**–**B6**, and **C1**–**C6** oxygen and nitrogen atoms in 2-furanyl, 2-pyridinyl, 3-pyridinyl groups are oriented in an “outer” direction with respect to center of the molecules (Figure 2c). Sulfur atoms are oriented in the same way in 2-thiophenyl group placed in 3, 5 positions; substitutes R_1_ and R_2_, and 2-thiophenyl groups are arranged as shown in a Figure 2e. For further calculations of molecular structure and spectra for **C1**–**C6** the conformer IV (Figure 3) was used: one fluorine atom forms two hydrogen-bonding interactions with two H_2-Sub_ atoms of C_6_H_5_-groups. For **B1**–**B6** and **C1**–**C6** molecules, substituents R_1_ and R_2_ are located quasi-parallel to each other (Figure 2a).

The different arrangement of the ethyl groups (Figure 2f,g) does not introduce large energy changes. According to the results of CAM-B3LYP/6-31+G(d,p) calculations, the energy differences between C_s_ and C_2_ conformers (Figure 2f,g) of **B7** do not exceed 0.03 kJ mol^−1^. However, both of these conformers lie on the PES higher by ∼0.3 kJ mol^−1^ than the conformer h (Figure 2h), in which the ethyl groups are located in a plane of the molecule and are oriented by “outer” direction with respect to center of molecule.

The optimized structures from QC calculations at CAM-B3LYP/6-31+G(d,p) level are given in Supplementary Materials. In Tables S1–S4, the calculated structural parameters of aza-BODIPY derivatives of the considered molecules are compared to each other.

### 2.6. Substitution Effect in Tetra-Substituted Aza-BODIPYs

The introduction of substituents R_1_ and R_2_ into the parent compound **D7** in positions C_1_/C_7_ leads to elongation of the bond length of the C_1_–C_8a_ by ∼0.013 Å (Table S4), while the substitution of hydrogen atoms by groups R_3_ and R_4_ in positions 3 and 5 leads to some shortening of these bonds (Tables S1–S3). Bond lengths of the C_2_–C_3_ are elongated with the introduction of substituents R_3_, R_4_ and shortened with the introduction of the substituents R_1_, R_2_. The most significant changes in the internuclear distance with the introduction of substituents are observed for the internuclear distances N_4a_-C_3_ and C_1_–C_8a_, on average 0.018 Å and 0.011 Å for **A1**–**A6**, **B1**–**B6**, and **C1**–**C6**. Geometry of the central 6-membered ring is only slightly affected by the introduction of substituents: the maximum change in the distances is 0.006 Å compared to analogous values for **D7**.

Compounds, which are equipped with aromatic rings in different positions of the dipyrrin backbone have extended π-delocalization. Apparently, the greatest delocalization is characteristic to the molecules containing 2-thiophenyl and 2-furanyl substituents. This is confirmed by slightly increased values of Wiberg bond indexes (WI) obtained by natural atomic orbital analysis: for molecules **A1**–**A7**, **A2**–**D2**, and **A3**–**D3**—WI(C_3_-C_thiophenyl_) ≈ WI(C_3_-C _furanyl_) ≈ 1.12, whereas for 2-methylthiophene and 2-methylfuran—WI(C(CH_3_)-C(ring)) = 1.04. Moreover, the dihedral angle for the 2-thiophenyl and 2-furanyl groups is smaller than for other groups; it can also lead to π-delocalization. It is known that the aza-BODIPY is seen to exhibit less aromatic character than the BODIPY [34].

**Figure 8:**
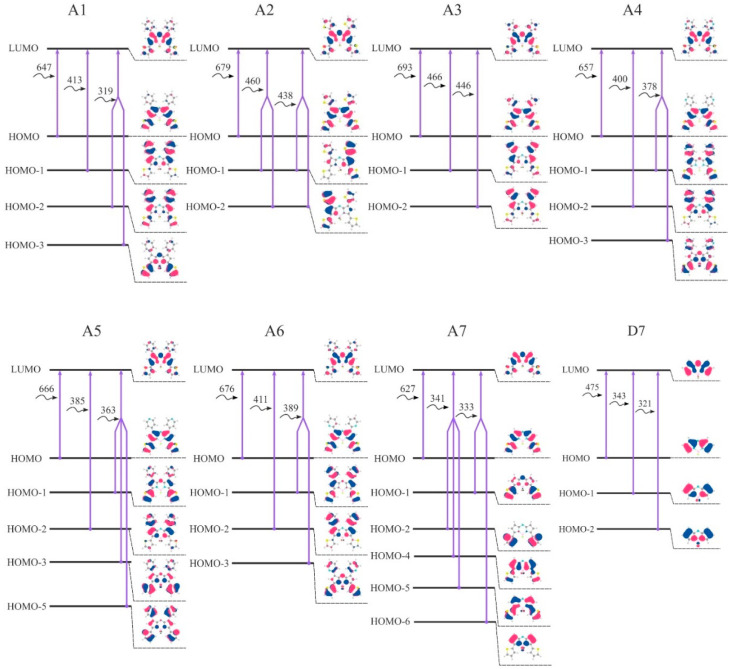
Visual representation of the first three transitions (nm) for molecules **A1**–**A7** and **D7** calculated at CAM-B3LYP/6-31+G(d,p) level. Isosurface cutoff is 0.03. Visual representation of transitions for molecules **B1**–**B7**, **C1**–**C7**, and **C1**–**D7** is presented in Figures S5–S7.

**Figure 9:**
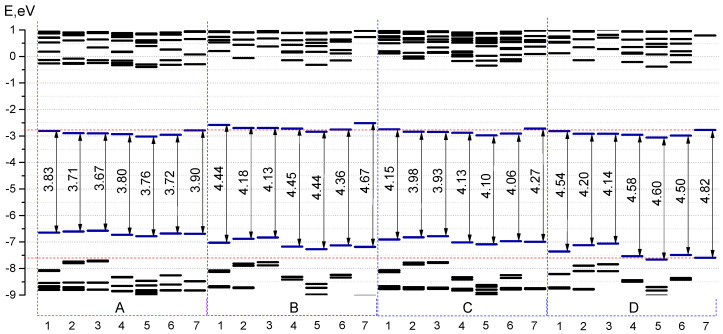
The CAM-B3LYP/6-31+G(d,p) MO energies and HOMO–LUMO energy gaps for **A1**–**A7**, **B1**–**B7**, **C1**–**C7**, and **D1**–**D7**.

A HOMO–LUMO gap is basically the energy that must be fed to the molecule to kick it from the ground state to an excited state [19]. A smaller energy gap leads to a redshift in the absorption spectrum. Thereby, studying a HOMO–LUMO gap is of great importance during the design of effective dyes. Introduction of the considered aryl substituents increases the energy of the HOMO orbitals, lowers the energy of the LUMO and reduces the HOMO-LUMO gap. The distribution patterns of frontier canonical MOs of the molecules, studied in this work, are shown in Figure 8 and Figures S5–S7. The HOMO of unsubstituted **D7** is localized on the carbon atoms. The HOMOs of aza-BODIPY derivatives display a pronounced resemblance with a strong contribution of the core (the contributions to the HOMOs exceeds 50%) and additionally on aryl moieties (Figure 8 and Table S15). Especially for ethyl-substituted, the predicted HOMOs and LUMOs are predominantly localized at the aza-BODIPY’s core. For **B1**–**B7**, HOMO-LUMO-gaps decrease slightly compared to **A1**–**A7** and **C1**–**C7**. The smallest HOMO–LUMO energy gaps are found for **A3** and **A2** molecules (Figure 9). It is noteworthy that the molecules with 2-thiophenyl and 2-furanyl groups are characterized by the biggest contribution of substituent groups to the frontier MOs: for **A3**—43% and 31% to HOMO and LUMO, respectively. Table S15 shows the calculated characteristics of contributions of different parts of molecules to HOMO and LUMO.

The HOMO–LUMO gap changes if the position of aryl groups varies from 3 and 5 positions to 1 and 7 positions. In case of 2-thiophenyl groups it is found to be 3.90 and 4.20 eV for **A7** and **D2**, respectively; in case of phenyl groups—4.27 and 4.54 eV for **C7** and **D1**, respectively. It is important to note that the pyridyl group, depending on the location of the nitrogen atom, has a different effect on the energetic properties: the HOMO-LUMO gap for 2-pyridinyl substituted aza-BODIPYs (series **A6**–**D6**) is smaller than the corresponding molecules from the series **A4**–**D4** and **A5**–**D5** (Figure 9).

### 2.7. Spectral Properties: TDDFT Electronic Absorption Spectra

**Figure 10:**
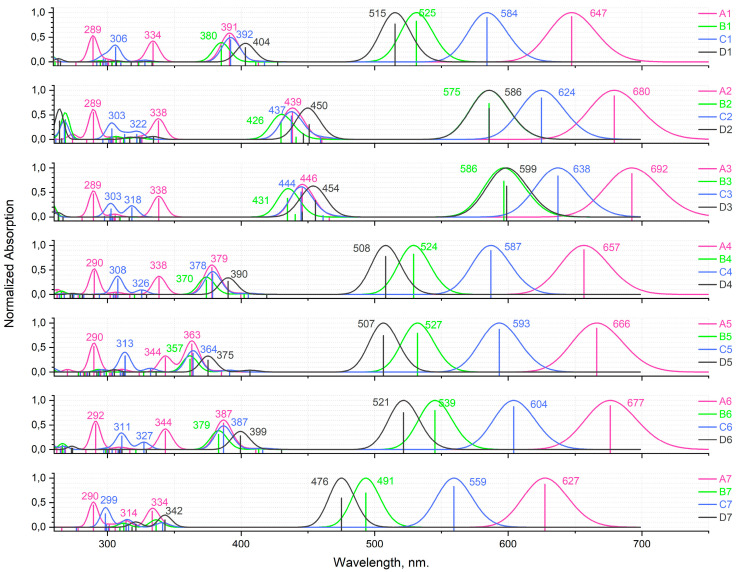
Calculated TDDFT (CAM-B3LYP/6-31+G(d,p)) electronic absorption spectra for aza-BODIPY derivatives.

Calculated (CAM-B3LYP/6-31+G(d,p)) TDDFT electronic absorption spectra of the studied substances are presented in Figure 10. The calculated oscillator strengths for the lowest allowed excited states along with their composition are given in Table S16. The comparison of the calculated spectra of aza-BODIPY dyes demonstrates a strong influence of the substituents. A strong bathochromic shift occurs with a change of hydrogen atoms by aryl groups (Figure 10). The absorption bands in the theoretical spectra are underestimated in comparison with the experimental data for **A1**–**A2**, **C1**–**C2** [35,36,37,38,39] by ∼64 nm. The observed discrepancy is due to the high multi-reference character of the dipyrrin complexes, as was previously shown by Brown and Momeni [40]. They have found that any TDDFT functional will systematically underestimate exicitation energies by ~0.2–0.5 eV.

The first electronic transition is the most interesting one, as for aza-BODIPY dyes it tends to be the strongest one. If one takes a look at Figure 8, where the first three transitions for each considered dye are summed up in terms of canonical orbitals contributing to each spectral line, it becomes apparent that the first absorption peak is always due mainly to the HOMO-LUMO transition. Nevertheless, the energy of the first and all other transitions is varying quite significantly.

Having examined the orbitals of **B1**–**B6**, it becomes apparent that 3,5-ethyl substituents have a minimal effect on the electronic transitions beyond the first one. Even for the first one, **B1**–**B6** consistently show the lowest red shift of absorption in each respective group. It is clear that the aryl substituents have the ability to conjugate their π-electron system, if one is present, with the π-electron system of the dipyrrin. As such, to get the maximum bathochromic shift it is necessary to use aromatic substituents.

Another clear trend emerges between the **A1**–**A7** and respective **C1**–**C7** compounds. **A1**–**A7** dyes consistently have a bigger red-shift of the first peak of absorption compared to the **C1**–**C7** dyes. This is in line with the previously reported experimental data on the subset of compounds considered here [41]. This is also confirmed by the higher contribution of substituent-localized orbitals to the HOMO/LUMO of **A1**–**A7** compounds compared to **C1**–**C7** compounds.

It is interesting to note that when it comes to the C_1_/C_7_ substituents, phenyl ring turns out to perform the worst compared to all considered heterocycles outperforming only the hydrogen-substituted **A7**, **B7**, or **C7** dyes. This is very promising for the aza-BODIPY chemistry and opens up a lot of opportunities for different heterocycle-substituted dyes and their more functionalized derivatives.

The biggest red shift of the first electronic transition is achieved by **A3**—2-furanyl substituted dye. In fact, 2-furanyl-substituted dyes are the best in their respective series. Their orbital compositions are very close to the closest analogues (such as **A2**), but the lower dihedral angle between the dipyrrin and furanyl fragments allows for a stronger π-electron conjugation. This correlation holds for all considered substituents.

In this regard, the case of **A4**–**A6**, **B4**–**B6**, and **C4**–**C6** dyes is of great interest. Here, a clear progression of red shift of first absorption peak is present, namely, **A6** > **A5** > **A4, B6** > **B5** > **B4**, and **C6** > **C5** > **C4.** HOMO and LUMO compositions between all of them are quite similar. However, the dihedral angle between 2-pyridinyl and dipyrrin is much less than between 3- and 4-pyridinyl and dipyrrins. The reason why **A5**–**C5** dyes possess slightly higher bathochromic shift than **A4**–**C4** dyes, however, cannot be explained so that the difference in the relevant angle is minimal. The reason for the shift is due to the fact that in 3-pyridinyl compound HOMO is localized on a nitrogen atom instead of a carbon one. This opens up a lot of opportunities for multiple-heteroatom heterocycles, such as pyrimidine, to become potentially valuable aza-BODIPY substituents.

When it comes to the second transition and beyond, the picture becomes less clear. In the CI singles determinant expansion, each determinant, and, by extension, each virtual orbital will have an associated weight; most of the time, it will be small but not zero. Here, we chose to only show contributions from determinants that make up at least 5% of all contributions. More often than not only one determinant has a significant contribution with additional ones giving 7–16%. However, this is not always the case.

Notable exceptions include the fourth and the fifth transitions of **C1**–**C7** dyes, as well as the fourth transitions of almost all of **B1**–**B7** dyes and the fifth transitions of **A1**–**A7** dyes. One interesting derivative is **B7**, with its third, fourth and fifth transitions having notable multi-determinant character. A direct correlation between the value of red-shift and the “complexity” of the relevant transition does not appear to exist. Similarly, there is no clear correlation between structural parameters, such as dihedral angles, and transitions multi-determinant character.

### 2.8. Vibrationally Resolved Absorption and Emission Spectra

To better understand the relationship between spectral properties and structures of examined compounds, we have computed the vibrationally resolved absorption and emission spectra. It should be noted that in order to minimize CPU time, the calculations were carried out with a smaller basis set (6-31G(d)) compared to TDDFT calculations described in the Section 2.7.

**Figure 11:**
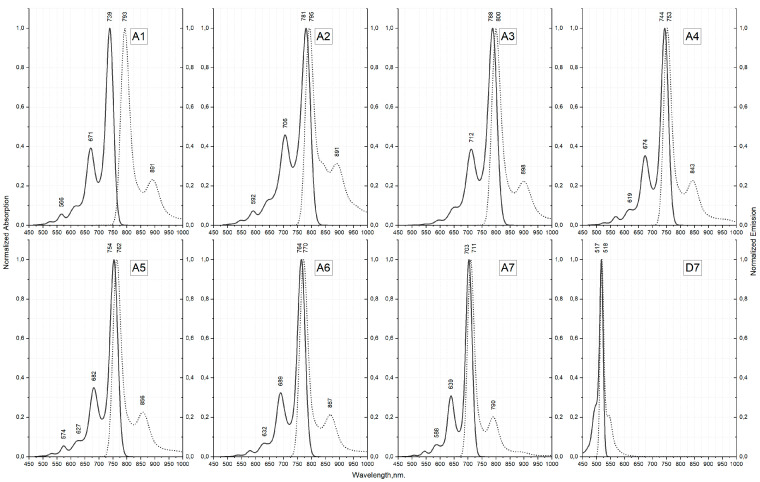
Calculated vibronic (FC, CAM-B3LYP/6-31G(d)) electronic absorption and emission spectra for **A1**–**A7** and **D7** aza-BODIPY derivatives. Solid lines correspond to absorption and dotted lines correspond to emission.

Obtained data for molecules **A1**–**A7** is illustrated in Figure 11 and Figures S8–S10 for the other ones. Herein, a notable clarification is due. Because of the non-planarity of the lowest conformers for **B2**, **D2** compounds in the ground state and their planarity in the first excited state, the FC (and even FCHT) approximation breaks down. This shortcoming of “constant dipole” or “first order change only” approximations has been previously noted [42]. New approaches, such as Nuclear Electronic Orbital TDDFT (NEO-TDDFT) [43] are being developed, but as of right now we have chosen to instead focus on other conformations for affected compounds. For those molecules, instead of a preferred position “e” for 2-thiophenyl groups (Figure 2), position “c” was taken instead.

For the four previously obtained compounds (**A1**, **A2**, and **C2**) calculated spectra are in reasonable agreement with experiment (Table S17) [41]. Although the characteristic shoulder is present for all compounds, its intensity is slightly overestimated. For substituted derivatives (all except **D7**) the separation between the main band and the shoulder is overestimated. Those issues are inevitable due to the approximate nature of exchange-correlation functionals which results in an underestimation of vibrational frequencies by ~5–10% for ground state (S0) and even more for excited state (S1).

Optimization of excited state geometries allows including some spurious orbital relaxation. It usually means that for excitation energies ΔE^adia^ < ΔE^vert^. However, all of the trends observed for vertical excitations (Figure 11) still hold for the adiabatic ones, i.e., for aryl substituted compounds, such as **A1**–**A6** and **C1**–**C6**, there is still a notable red shift of both absorption and fluorescence compared to others. It is clear then that the additional conjugation of the dipyrrin π-electronic system with aromatic substituents is necessary to achieve near-IR absorption and emission, with positions C_3_/C_5_ being more important than C_1_/C_7_. The largest bathochromic shift is observed for compound **A3** with the absorption maximum being around 788 nm and emission maximum around 800 nm. A3 is followed closely by **A2**, which has maxima of 781 and 795 for absorption and emission respectively. It is interesting that **A6** displays higher red shift than **A5** and **A4**. This correlates nicely with the dihedral angle between the substituents and the dipyrrin. Indeed, the lower angle can allow for the increased conjugation and higher red-shift.

Another nod to the importance of C_3_/C_5_ substitution of Aza-BODIPY dyes is the **D1**–**D6** series. Many of those compounds have vibronic absorption peaks at wavelengths lower than 600 nm even though they have aromatic substituents. In contrast, **A7** and **C7**, which also have only two substituents but in positions C_3_ and C_5_, show absorption maxima at 703 nm and 622 nm, respectively. Introduction of ethyl groups into the C_3_/C_5_ (**B1**–**B7**) does not change the position of the peaks significantly.

A useful feature of the vibronic spectra is the ability to analyze which normal modes are coupled to the electronic excitations. A rational examination can help to explain the observed line shapes and design a new set of molecules that better suit a given application. To that end, we have examined the primary molecular vibrations, which affect the spectra of Aza-BODIPY dyes. This task is difficult, as many of the vibrational levels give small contributions. Figures S11–S14 show the scaled displacement vectors for some of the main molecular vibrations affecting the line shapes of absorption/emission in given series of Aza-BODIPY derivatives. The main absorption and emission bands involve 1–2 high intensity transitions—rotations of C_3_/C_5_ and C_1_/C_7_ substituents around the substituent-dipyrrin bond. For **D7**, which has no substituents, the vibronic structure is completely different—main contribution comes from out-of-plane dipyrrin distortions. Quite interesting is the fact that for pyridine-substituted molecules, there is a higher number of average-to-low intensity vibronic transitions making it complicated to analyze, so much so for **A4**–**A6** that there is essentially no dominant contributions. For shoulder in spectra, the most intense transition involves in-plane stretching of R3, R4-substituent C-H bonds (C_2-sub_-H, C_3-sub_-H, etc.). In case of **D1**–**D7**, which do not have any C_3_/C_5_ substituents, the shoulder corresponds to the in-plane stretching of dipyrrin C_2_-C_3_, C_3_-C_4_ and C_2_-H, C_3_-H bonds.

Based on the calculated vibronic spectra, we have compared Stokes shifts of studied compounds. Stokes shift is the difference between the wavelengths of absorption and emission peaks. Here, to simulate our line shapes, we have used Gaussian broadening with HWHM of 250 cm^−1^. If we chose to use different half widths at half maximums (HWHM), the Stokes shift values would change too. However, by using the same HWHM for all compounds we can obtain qualitative information about the Stokes shift trends.

Introduction of ethyl substituents into the C_3_/C_5_ positions generally affects Stokes shift very little as **B7** has very close line shape to **D7**. Introduction of aromatic substituents into those positions increases the calculated shift—a little bit for the 2-thiophenyls (**A7**) and more so for the phenyls (**C7**). When it comes to the C_1_/C_7_ substituents, all molecules display similar amount of Stokes shift given the same C_3_/C_5_ group. 2-pyridine-substituted molecules (**A6**, **B6**, **C6**, and **D6**) have the smallest, if only barely, increase in their respective rows (Figure 11 and Figures S8–S10). There exists a subtle correlation between the dihedral angle between the substituent and the dipyrrin and the amount of calculated Stokes shift—the more planar the molecule overall the less shift there is. This, however, is not due to the change in the vibrational levels of compounds. If we compare 0^0^ → 1^5^ (here electronic^vibrational^ notation is used) transition contributions between **A4**, **A5**, and **A6** we obtain values of 3.5, 3.5 and 3.8 a.u. Rather than this, a significantly increased 0^0^ → 1^0^, usually referred to as the 0-0, transition contribution (6.5, 6.4, and 8.1 a.u., respectively) results in a lower shift. The higher end of the calculated shifts (~20 nm) is not enough for some specific uses in medicine and biochemistry that require cell imaging with high signal-to-noise ratio.

## 3. Materials and Methods

### Computational Details

All meta-dynamical calculations were performed using the CREST program [44] with XTB [26]. The more accurate GFN2-XTB method was chosen along with the default iMTD-GC algorithm and energy thresholds. Each run concluded with the Hessian analysis of all obtained conformers and a subsequent resorting based on the Gibbs free energy.

All DFT calculations were performed using the Gaussian 09 program package [45]. Calculations of the potential energy surface (PES) scan of aza-BODIPY derivatives were done in the framework of DFT/PBE [46] method in combination with the 6-31G(d) basis set. In order to study the internal rotations χ_1_, χ_2_, χ_3_, χ_4_, PES scan was done using changes in the dihedral angles C_2_-C_1_-C_1-Sub_-X_2-Sub_ (χ_1_), C_6_-C_7_-C_1-Sub_-X_2-Sub_ (χ_2_), C_2_-C_3_-C_1-Sub_-X_2-Sub_ (χ_3_), C_6_-C_5_-C_1-Sub_-X_2-Sub_ (χ_4_, Figure 1) with the step of 10°. All calculations accounted for the relaxation of the structure.

In the second step geometric structure, harmonic vibrations of aza-BODIPY derivatives were done in the framework of DFT/CAM-B3LYP [29] method. The basis set 6-31+G(d,p) was used. CAM-B3LYP/6-31+G(d,p) calculations used CH_2_Cl_2_ solvent parameters for easy comparison to the experimental data due to its prevalence in the aza-BODIPY synthesis procedure. The molecules in the solvated state were determined using CPCM [30,31]. Electronic absorption spectra of aza-BODIPY derivatives were simulated on the basis of TDDFT calculations with the use of Gaussian 09 program [45]. The natural bond orbital analysis was performed as it is implemented in Gaussian 09 [45]. The contributions of atomic orbitals of specific atoms to MOs are calculated based on the results of QC calculations using the GausSum program [47]. The molecular models and orbitals demonstrated in the paper were visualized by means of the Chemcraft program [48].

To study hydrogen bonds quantitative QTAIM analysis was performed using AIMAll software package [49]. For hydrogen bonds the presence of critical point (3,−1) is necessary and sufficient, values of electron density distribution function ρ(r) and ∇ρ(r) in BCPs should lie in the range from 0.002 to 0.035 a.u. and from 0.024 to 0.139 a.u., respectively [50].

All calculations of vibronic spectra were performed in Gaussian 09 suit of programs. We have followed suggestions by Fortino et al. [51]. Time-independent formalism utilized prescreening with parameters C1 and C2 both equaling 30 and maximum number of integrals of 5 × 1010. Employed gaussian spectral broadening used HWHM = 250 cm^−1^.

## 4. Conclusions

The structures of 27 aza-BODIPY derivatives were studied in comparison with the structure of parent-aza-BODIPY. Some contradictions were found in the structural parameters obtained by various methods; however, these differences do not give significant changes in the predicted spectral characteristics. It was noted that the cyclic substituents are not located in the plane of the heterocycle: the dihedral angles χ responsible for group rotations are 8–37°. This behavior of the substituents and the gently sloping PES in the range of χ ≈ [−20°; 20°] leads to the appearance of a large number of conformers associated with different mutual arrangement of groups relating to each other. Energy differences between these conformers are small, therefore it can be concluded that several conformers are energetically accessible for many compounds considered in the work. The addition of substituents leads to an elongation of the C_3_-N and C_1_-C_8a_ bonds by ∼0.2 and ∼0.1 Å. The introduction of substituents causes strong bathochromic shifts in electronic absorption spectra. The substitution of a hydrogen atom by a cyclic substituent in position C_3_ (Figure 1) leads to great changes in the energy of frontier molecular orbitals compared to the substitution in position 1 (Figure 1). The smallest HOMO–LUMO energy gaps and the biggest bathochromic shifts were observed for 2-thiophenyl and 2-furanyl substituted molecules (**A2** and **A3**).

## Supplementary Materials

The following are available online, Figure S1: Conformations of **A1**–**A7** dyes according to the GFN2-XTB/CREST meta-dynamical calculations, Figure S2: Conformations of **B1**–**B7** dyes according to the GFN2-XTB/CREST meta-dynamical calculations, Figure S3: Conformations of **C1**–**C7** dyes according to the GFN2-XTB/CREST meta-dynamical calculations, Figure S4: Conformations of **D1**–**D7** dyes according to the GFN2-XTB/CREST meta-dynamical calculations, Figure S5: Visual representation of the first three transitions for molecules **B1**–**B7** calculated at CAM-B3LYP/6-31+G(d,p) level, Figure S6: Visual representation of the first three transitions for molecules **C1**–**C7** calculated at CAM-B3LYP/6-31+G(d,p) level, Figure S7: Visual representation of the first three transitions for molecules **D1**–**D7** calculated at CAM-B3LYP/6-31+G(d,p) level, Figure S8: Calculated vibronic (FC, CAM-B3LYP/6-31G(d)) electronic absorption and emission spectra for **B1**–**B7** aza-BODIPY derivatives, Figure S9: Calculated vibronic (FC, CAM-B3LYP/6-31G(d)) electronic absorption and emission spectra for **C1**–**C7** aza-BODIPY derivatives, Figure S10: Calculated vibronic (FC, CAM-B3LYP/6-31G(d)) electronic absorption and emission spectra for **D1**–**D7**, Figure S11: Scaled displacement vectors for some of the main molecular vibrations affecting the line shapes of absorption/emission for **A1**–**A3**, Figure S12: Scaled displacement vectors for some of the main molecular vibrations affecting the line shapes of absorption/emission for **B1**–**B7**, Figure S13: Scaled displacement vectors for some of the main molecular vibrations affecting the line shapes of absorption/emission for **C1**–**C6**, Figure S14: Scaled displacement vectors for some of the main molecular vibrations affecting the line shapes of absorption/emission for **D1**–**D7**, Table S1: Molecular parameters of **A1**–**A7** optimized at CAM-B3LYP/6-31+G(d,p) level, Table S2: Molecular parameters of **B1**–**B7** optimized at CAM-B3LYP/6-31+G(d,p) level, Table S3: Molecular parameters of **C1**–**C7** optimized at CAM-B3LYP/6-31+G(d,p) level, Table S4: Molecular parameters of **D1**–**D7** optimized at CAM-B3LYP/6-31+G(d,p) level, Table S5: Rotation angles χ_1_ (°) for aza-BODIPY derivatives according to PBE/6-31G(d) calculations, Table S6: Relative energies (kJ mol^−1^) of conformers II in relation to conformer I for **A1**–**A6**, Table S7: Natural charges at atoms calculated in the framework of natural population analysis for **A1**–**A7**, Table S8: Natural charges at atoms calculated in the framework of natural population analysis for **B1**–**B7**, Table S9: Natural charges at atoms calculated in the framework of natural population analysis for **C1**–**C7**, Table S10: Natural charges at atoms calculated in the framework of natural population analysis for **D1**–**D7**, Table S11: Wiberg bond indexes calculated for A1-7, Table S12: Wiberg bond indexes calculated for **B1**–**B7**, Table S13: Wiberg bond indexes calculated for C1-7, Table S14: Wiberg bond indexes calculateda for **D1**–**D7**, Table S15: Contributions of aza-BODIPY core and substituent groups R_1_-R_4_ to frontier MOs of **A1**–**A7**, **B1**–**B7**, **C1**–**C7**, **D1**–**D7**, Table S16: Calculated (CAM-B3LYP/6-31+G(d,p)) composition of five lowest excited states and corresponding oscillator strengths, Table S17: Experimental and theoretical (CAM-B3LYP/6-31G(d)) bands of absorption and emission spectra. Additionally, the optimized structures from QC calculations at CAM-B3LYP/6-31+G(d,p) and CAM-B3LYP/6-31G(d) levels are given in Supplementary Materials.

## Abbreviations

**A1**	1,7-diphenyl-3,5-di(2-thiophenyl)-aza-BODIPY
**A2**	1,3,5,7-tetra(2-thiophenyl)-aza-BODIPY
**A3**	1,7-di(2-furanyl-3,5-di(2-thiophenyl)-aza-BODIPY
**A4**	1,7-di(3-pyridinyl)-3,5-di(2-thiophenyl)-aza-BODIPY
**A5**	1,7-di(4-pyridinyl)-3,5-di(2-thiophenyl)-aza-BODIPY
**A6**	1,7-di(2-pyridinyl)-3,5-di(2-thiophenyl)-aza-BODIPY
**A7**	3,5-di(2-thiophenyl)-aza-BODIPY
**B1**	1,7-diphenyl-3,5-diethyl-aza-BODIPY
**B2**	1,7-di(2-thiophenyl)-3,5-diethyl-aza-BODIPY
**B3**	1,7-di(2-furanyl)-3,5-diethyl-aza-BODIPY
**B4**	1,7-di(3-pyridinyl)-3,5-diethyl-aza-BODIPY
**B5**	1,7-di(4-pyridinyl)-3,5-diethyl-aza-BODIPY
**B6**	1,7-di(2-pyridinyl)-3,5-diethyl-aza-BODIPY
**B7**	3,5-diethyl-aza-BODIPY
BCP	Bond critical point
BODIPY	4,4′-difluoro-4-bora-3a,4a-diaza-s-indacene
**C1**	1,3,5,7-tetraphenyl-aza-BODIPY
**C2**	1,7-di(2-thiophenyl)-3,5-diphenyl-aza-BODIPY
**C3**	1,7-di(2-furanyl)-3,5-diphenyl-aza-BODIPY
**C4**	1,7-di(3-pyridinyl)-3,5-diphenyl-aza-BODIPY
**C5**	1,7-di(4-pyridinyl)-3,5-diphenyl-aza-BODIPY
**C6**	1,7-di(2-pyridinyl)-3,5-diphenyl-aza-BODIPY
**C7**	3,5-diphenyl-aza-BODIPY
CAM-B3LYP	The CAM-B3LYP functional (Coulomb-attenuating method, Becke, 3-parameter, Lee–Yang–Parr)
CPCM	Conductor-like polarizable continuum model
**D1**	1,7-diphenyl-aza-BODIPY
**D2**	1,7-di(2-thiophenyl)-aza-BODIPY
**D3**	1,7-di(2-furanyl)-aza-BODIPY
**D4**	1,7-di(3-pyridinyl)-aza-BODIPY
**D5**	1,7-di(4-pyridinyl)-aza-BODIPY
**D6**	1,7-di(2-pyridinyl)-aza-BODIPY
**D7**	Aza-BODIPY
DFT	Density functional theory
DSSC	Dye-sensitized solar cell
HWHM	Half width at half maximum
HOMO	Highest occupied molecular orbital
LUMO	Lowest unoccupied molecular orbital
MO	Molecular orbital
NIR	Near-infrared
PBE	The PBE functional (Perdew–Burke-Ernzerhof)
PES	Potential energy surface
TDDFT	Time-dependent density-functional theory
QC	Quantum chemical calculations
QTAIM	Quantum theory of atoms in molecules
RMSD	Root mean square deviation
XRD	X-ray diffraction
