# Supplementary material for: Quantum Chemical Study Aimed at Modeling Efficient Aza-BODIPY NIR Dyes: Molecular and Electronic Structure, Absorption, and Emission Spectra
Source: Molecules. 2020 Nov 17;25(22):5361. doi: 10.3390/molecules25225361 (PMC7698449; doi:10.3390/molecules25225361)
Supplement: Supplementary file 1 [file molecules-25-05361-s001.zip › molecules-970498-supplementary.pdf]

Supplementary materials

Quantum Chemical Study Aimed at Modeling Efficient Aza-BODIPY NIR Dyes: Molecular and Electronic Structure, Absorption, and Emission Spectra

Alexander E. Pogonin [1,*], Artyom Y. Shagurin [2], Maria A. Savenkova [1], Felix Yu. Telegin [2], Yuriy S. Marfin [2] and Arthur S. Vashurin [2]

1 Department of Nanomaterials and Ceramic Technology, Ivanovo State University of Chemistry and Technology, Sheremetevsky av., 7, 153000 Ivanovo, Russia; mariasavenkova@bk.ru

2 Department of Inorganic Chemistry, Ivanovo State University of Chemistry and Technology, Sheremetevsky av., 7, 153000 Ivanovo, Russia; Shagurin.A.Y@gmail.com (A.Y.S.); f.telegin@mail.ru (F.Y.T.); marfin@isuct.ru (Y.S.M.); vashurin@isuct.ru (A.S.V.)

* Correspondence: pogonin@isuct.ru; Tel. +7-(4932)-359-874

Figure S1. Conformations of **A1**–**A7** dyes according to the GFN2-XTB/CREST meta-dynamical calculations. Next to the molecules are Gibbs free energy values relative to the lowest conformer.

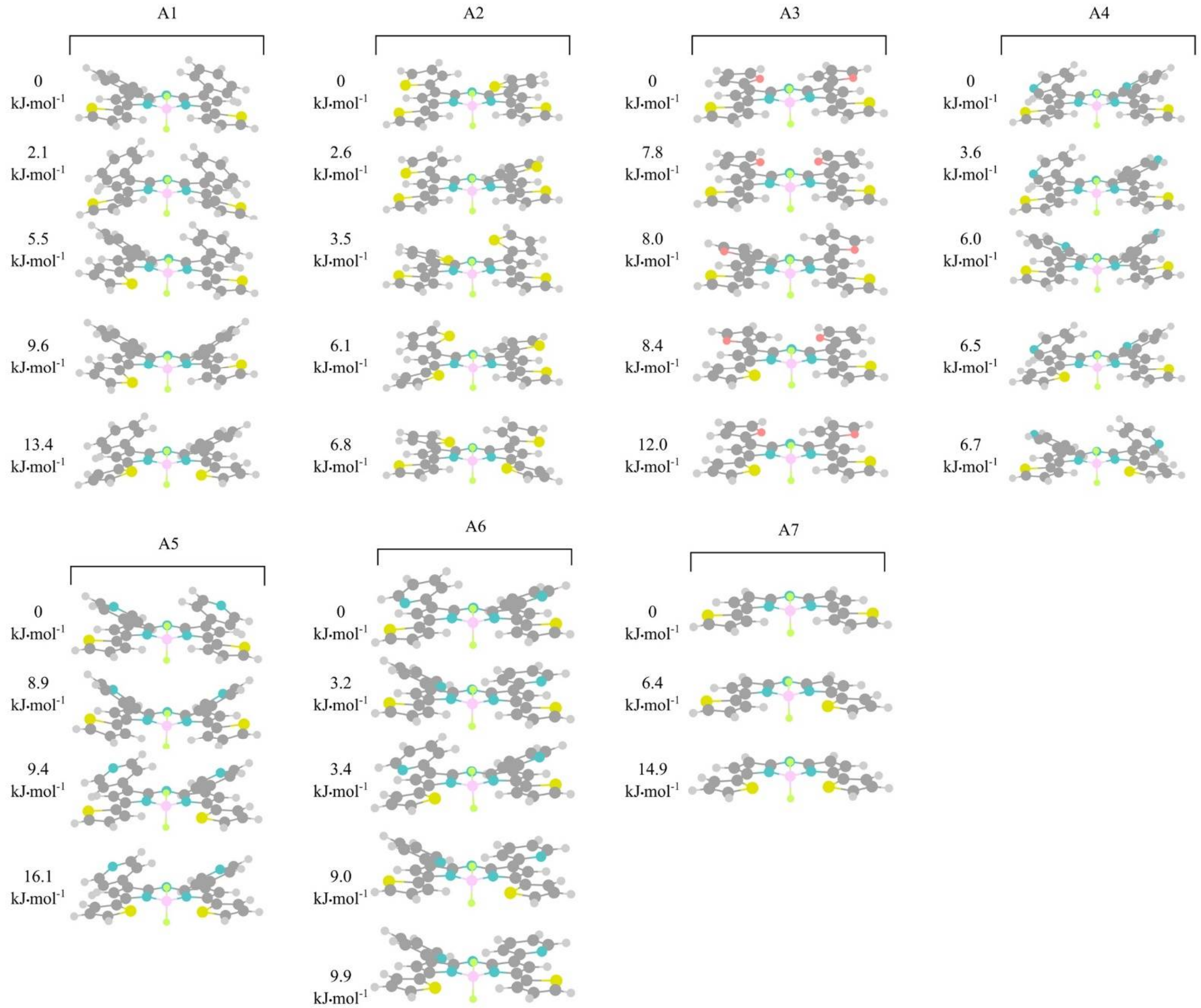

Figure S2. Conformations of **B1–B7** dyes according to the GFN2-XTB/CREST meta-dynamical calculations. Next to the molecules are Gibbs free energy values relative to the lowest conformer.

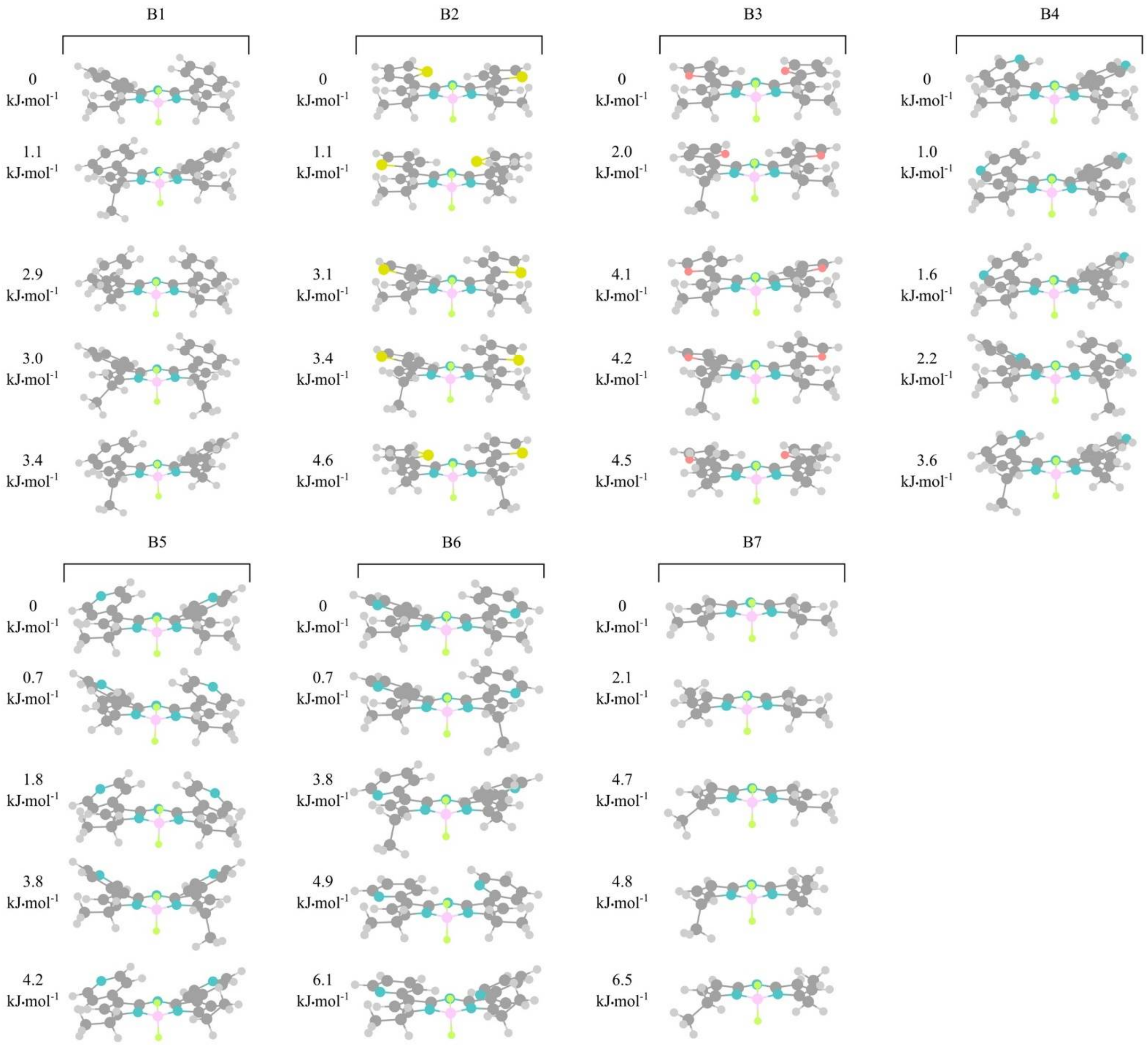

Figure S3. Conformations of **C1**–**C7** dyes according to the GFN2-XTB/CREST meta-dynamical calculations. Next to the molecules are Gibbs free energy values relative to the lowest conformer.

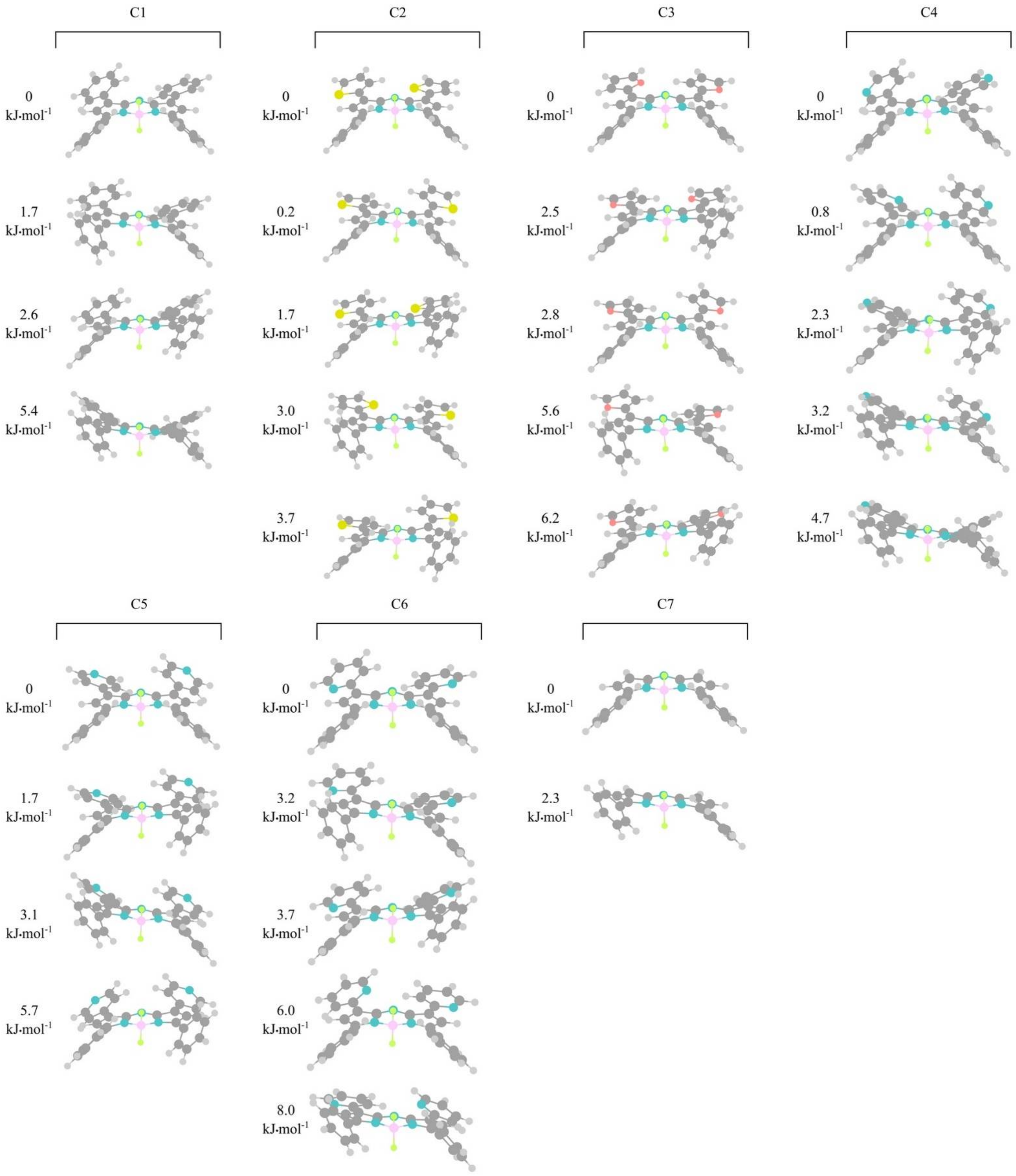

Figure S4. Conformations of **D1–D7** dyes according to the GFN2-XTB/CREST meta-dynamical calculations. Next to the molecules are Gibbs free energy values relative to the lowest conformer.

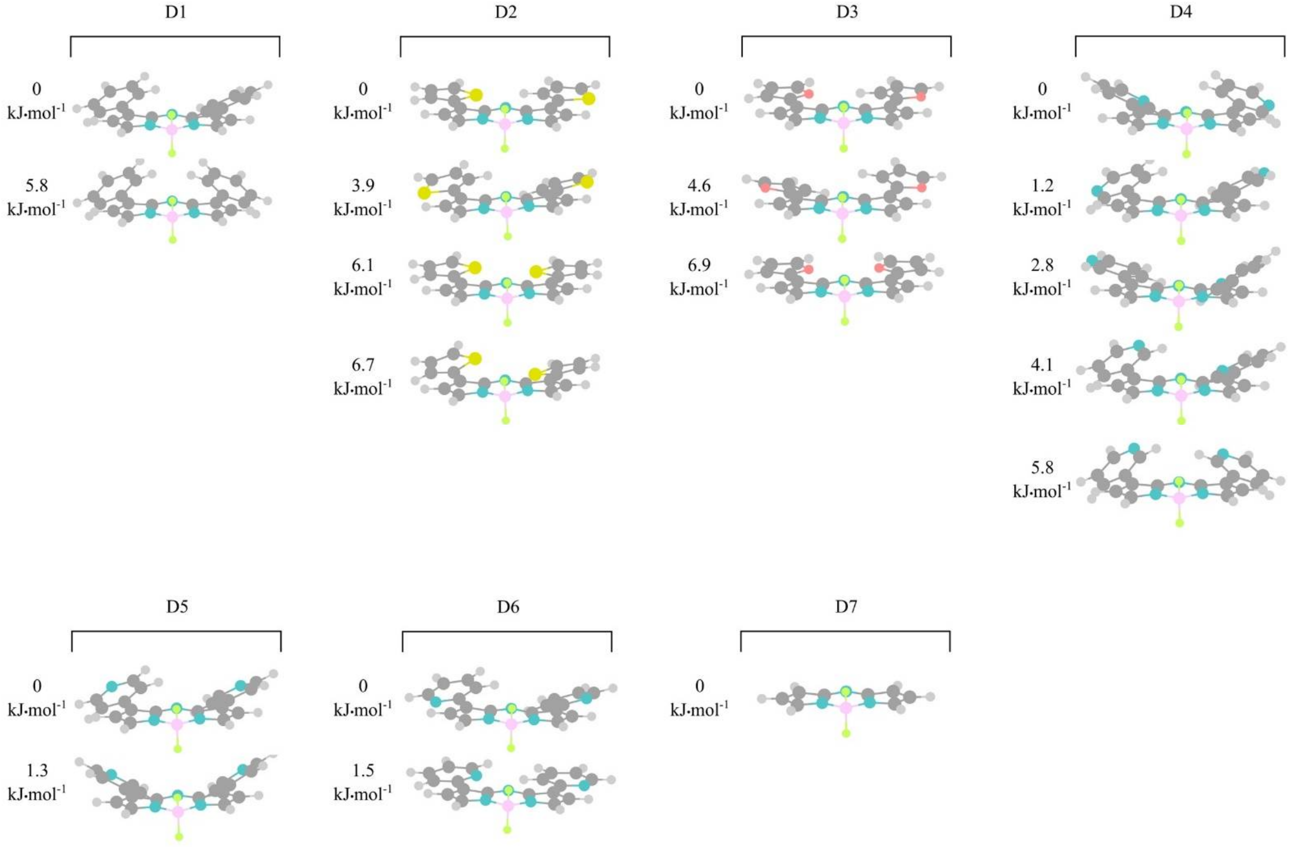

Table S1. Molecular parameters[a] of **A1-7** optimized at CAM-B3LYP/6-31+G(d,p) level.

	**A1**	**A2**	**A3**	**A4**	**A5**	**A6**	**A7**
Symmetry	C_2	C_1	C_2	C_2	C_2	C_2	C_2
$\lvert\chi(N_{4a}\text{-}B\text{-}N_{4b}\text{-}N_8)\rvert$	0.0	0.1	0.0	0.0	0.0	0.0	0.0
$\lvert\chi(C_2\text{-}C_1\text{-}C_{8a}\text{-}C_m)\rvert$	175.2	175.5	175.6	175.7	175.8	175.4	178.2
$\lvert\chi(C_3\text{-}N_{4a}\text{-}B\text{-}N_{4b})\rvert$	175.4	175.7	176.3	175.7	175.7	175.7	176.6
$\lvert\chi_1\rvert=\lvert\chi_2\rvert$ [b]	30.5	16.2 [b]	9.4	30.5	29.4	18.0	-
$\lvert\chi_3\rvert=\lvert\chi_4\rvert$	17.2	17.6	17.0	16.4	15.8	15.5	14.1
$r(C_1\text{-}X_{1\text{-}Sub})$	1.467	1.447	1.437	1.464	1.467	1.470	1.081
$r(C_3\text{-}C_{1\text{-}Sub})$	1.447	1.447	1.448	1.446	1.446	1.446	1.446
$r(C_{8a}\text{-}N_{4a})$	1.395	1.394	1.393	1.393	1.393	1.394	1.396
$r(C_3\text{-}N_{4a})$	1.357	1.359	1.361	1.357	1.357	1.358	1.356
$r(N_{4a}\text{-}B)$	1.550	1.550	1.550	1.552	1.552	1.550	1.552
$r(C_1\text{-}C_{8a})$	1.431	1.434	1.430	1.430	1.430	1.431	1.418
$r(N_8\text{-}C_{8a})$	1.316	1.315	1.316	1.316	1.316	1.316	1.318
$r(C_1\text{-}C_2)$	1.381	1.383	1.383	1.379	1.378	1.378	1.370
$r(C_2\text{-}C_3)$	1.416	1.413	1.413	1.418	1.419	1.417	1.427
$\varphi(C_{8a}\text{-}N_{4a}\text{-}B)$	122.2	122.4	122.2	122.2	122.2	122.3	122.1
$\varphi_e(N_{4a}\text{-}C_3\text{-}C_2)$	109.2	109.3	109.4	109.1	109.1	109.1	109.0
$\varphi(N_{4a}\text{-}B\text{-}N_{4b})$	107.0	106.9	106.9	107.0	107.0	107.0	107.0
$\varphi(C_1\text{-}C_2\text{-}C_3)$	108.6	108.5	108.1	108.4	108.4	108.5	107.8
$WI(C_1\text{-}C_{1\text{-}Sub})$	1.06	1.11	1.11	1.06	1.06	1.06	-
$WI(C_3\text{-}C_{1\text{-}Sub})$	1.13	1.12	1.12	1.13	1.13	1.13	1.13

[a] r - bond lengths in Å, φ – bond angles in degrees, χ - dihedral angle in degrees, WI - Wiberg bond indexes; [b] for **A2** – the average value of the deviation from the substituent's location in the plane, $\lvert\chi_1\rvert$=17.0 °, $\lvert\chi_2\rvert$=164.7 °. Natural charges and Wiberg bond indexes are given in Tables S7 and S11.

Table S2. Molecular parameters[a] of **B1-7** optimized at CAM-B3LYP/6-31+G(d,p) level.

	**B1**	**B2**	**B3**	**B4**	**B5**	**B6**	**B7**
Symmetry	C_2	C_1	C_2	C_2	C_2	C_2	C_{2v}
$\|\chi(N_{4a}\text{-}B\text{-}N_{4b}\text{-}N_8)\|$	0.0	0.1	0.0	0.0	0.0	0.0	0.0
$\|\chi(C_2\text{-}C_1\text{-}C_{8a}\text{-}C_m)\|$	177.0	178.6	177.9	177.5	177.6	177.2	180.0
$\|\chi(C_3\text{-}N_{4a}\text{-}B\text{-}N_{4b})\|$	178.9	179.8	179.7	179.1	179.2	179.1	180.0
$\|\chi_1\|=\|\chi_2\|$ [b]	29.3	13.4[b]	7.6	29.5	28.4	17.3	-
$\|\chi_3\|=\|\chi_4\|$	0.0	0.2 [c]	0.6	0.3	0.2	0.8	0.0
$r(C_1\text{-}X_{1\text{-}Sub})$	1.466	1.447	1.436	1.464	1.467	1.470	1.081
$r(C_3\text{-}C_{1\text{-}Sub})$	1.494	1.494	1.494	1.494	1.494	1.494	1.494
$r(C_{8a}\text{-}N_{4a})$	1.396	1.393	1.395	1.395	1.394	1.395	1.398
$r(C_3\text{-}N_{4a})$	1.346	1.350	1.350	1.346	1.345	1.346	1.344
$r(N_{4a}\text{-}B)$	1.545	1.542	1.544	1.547	1.548	1.546	1.548
$r(C_1\text{-}C_{8a})$	1.432	1.431	1.431	1.431	1.431	1.433	1.419
$r(N_8\text{-}C_{8a})$	1.317	1.318	1.318	1.317	1.317	1.318	1.319
$r(C_1\text{-}C_2)$	1.388	1.394	1.391	1.387	1.385	1.385	1.377
$r(C_2\text{-}C_3)$	1.410	1.405	1.406	1.412	1.413	1.411	1.421
$\varphi(C_{8a}\text{-}N_{4a}\text{-}B)$	123.5	123.5	123.5	123.5	123.5	123.5	123.5
$\varphi_e(N_{4a}\text{-}C_3\text{-}C_2)$	123.5	123.5	123.5	123.5	123.5	123.5	123.5
$\varphi(N_{4a}\text{-}B\text{-}N_{4b})$	105.6	105.5	105.5	105.5	105.5	105.6	105.5
$\varphi(C_1\text{-}C_2\text{-}C_3)$	108.3	108.1	107.8	108.1	108.1	108.2	107.5
$WI(C_1\text{-}C_{1\text{-}Sub})$	1.07	1.11	1.11	1.06	1.06	1.06	-
$WI(C_3\text{-}C_{1\text{-}Sub})$	1.03	1.03	1.03	1.03	1.03	1.03	1.03

[a] r - bond lengths in Å, φ – bond angles in degrees, χ - dihedral angle in degrees, WI - Wiberg bond indexes; [b] for **B2** – the average value of the deviation from the substituent's location in the plane, $|\chi_1|$=14.5 °, $|\chi_2|$=167.7 °; [c] average value for nonequivalent parameters $|\chi_3|$, $|\chi_4|$. Natural charges and Wiberg bond indexes are given in Tables S8 and S12.

Table S3. Molecular parameters[a] of **C1-7** optimized at CAM-B3LYP/6-31+G(d,p) level.

	**C1**	**C2**	**C3**	**C4**	**C5**	**C6**	**C7**
Symmetry	C_1	C_1	C_1	C_1	C_1	C_1	C_s
$\lvert\chi(N_{4a}\text{-}B\text{-}N_{4b}\text{-}N_8)\rvert$	9.5	9.5	9.5	9.5	9.4	9.5	9.3
$\lvert\chi(C_2\text{-}C_1\text{-}C_{8a}\text{-}C_m)\rvert$	174.2	173.5	173.9	173.7	174.1	173.7	174.3
$\lvert\chi(C_3\text{-}N_{4a}\text{-}B\text{-}N_{4b})\rvert$	169.2	168.6	168.9	168.8	169.2	168.8	169.4
$\lvert\chi_1\rvert=\lvert\chi_2\rvert$ [b]	29.9	25.4 [b]	8.7	30.0	28.7	17.6	-
$\lvert\chi_3\rvert\approx\lvert\chi_4\rvert$ [c]	36.6 [c]	37.1 [c]	37.0 [c]	36.3 [c]	36.1 [c]	36.0 [c]	35.6
$r(C_1\text{-}X_{1\text{-}Sub})$	1.466	1.446	1.437	1.464	1.466	1.469	1.081
$r(C_3\text{-}C_{1\text{-}Sub})$	1.469	1.469	1.469	1.468	1.468	1.468	1.468
$r(C_{8a}\text{-}N_{4a})$	1.396	1.396	1.395	1.395	1.394	1.395	1.398
$r(C_3\text{-}N_{4a})$	1.352	1.354	1.356	1.352	1.352	1.353	1.351
$r(N_{4a}\text{-}B)$	1.553	1.553	1.552	1.555	1.556	1.553	1.556
$r(C_1\text{-}C_{8a})$	1.432	1.434	1.431	1.431	1.430	1.431	1.418
$r(N_8\text{-}C_{8a})$	1.316	1.315	1.316	1.316	1.316	1.317	1.318
$r(C_1\text{-}C_2)$	1.383	1.386	1.385	1.382	1.380	1.380	1.372
$r(C_2\text{-}C_3)$	1.414	1.411	1.411	1.416	1.417	1.415	1.424
$\varphi(C_{8a}\text{-}N_{4a}\text{-}B)$	121.7	121.8	121.6	121.7	121.7	121.7	121.7
$\varphi_e(N_{4a}\text{-}C_3\text{-}C_2)$	109.4	109.5	109.6	109.4	109.3	109.3	109.3
$\varphi(N_{4a}\text{-}B\text{-}N_{4b})$	106.6	106.5	106.5	106.5	106.5	106.6	106.6
$\varphi(C_1\text{-}C_2\text{-}C_3)$	108.5	108.4	108.1	108.4	108.4	108.4	107.8
$WI(C_1\text{-}C_{1\text{-}Sub})$	1.06	1.11	1.11	1.06	1.06	1.06	-
$WI(C_3\text{-}C_{1\text{-}Sub})$	1.06	1.06	1.06	1.07	1.07	1.07	1.07

[a] r - bond lengths in Å, φ – bond angles in degrees, χ - dihedral angle in degrees, WI - Wiberg bond indexes; [b] for **C2** – the average value of the deviation from the substituent's location in the plane, $\lvert\chi_1\rvert$=18.9 °, $\lvert\chi_2\rvert$=149.2 °; [c] average values for nonequivalent parameters $\lvert\chi_3\rvert$, $\lvert\chi_4\rvert$, for **C7** – $\lvert\chi_3\rvert\approx\lvert\chi_4\rvert$. Natural charges and Wiberg bond indexes are given in Tables S9 and S13.

Table S4. Molecular parameters[a] of **D1-7** optimized at CAM-B3LYP/6-31+G(d,p) level.

	**D1**	**D2**	**D3**	**D4**	**D5**	**D6**	**D7**
Symmetry	C_2	C_1	C_2	C_2	C_2	C_2	C_{2v}
$\lvert\chi(N_{4a}\text{-}B\text{-}N_{4b}\text{-}N_8)\rvert$	0.0	0.4	0.0	0.0	0.0	0.0	0.0
$\lvert\chi(C_2\text{-}C_1\text{-}C_{8a}\text{-}C_m)\rvert$	176.8	178.2	178.0	177.3	177.4	177.0	180.0
$\lvert\chi(C_3\text{-}N_{4a}\text{-}B\text{-}N_{4b})\rvert$	178.7	179.4	179.6	178.9	178.9	178.9	180.0
$\lvert\chi_1\rvert=\lvert\chi_2\rvert$ [b]	28.8	12.5[b]	6.7	29.3	28.7	17.2	-
$r(C_1\text{-}X_{1\text{-}Sub})$	1.465	1.444	1.435	1.463	1.466	1.469	1.081
$r(C_3\text{-}H_{1\text{-}Sub})$	1.082	1.082	1.082	1.082	1.082	1.082	1.082
$r(C_{8a}\text{-}N_{4a})$	1.396	1.395	1.394	1.395	1.395	1.395	1.398
$r(C_3\text{-}N_{4a})$	1.337	1.338	1.340	1.336	1.335	1.336	1.334
$r(N_{4a}\text{-}B)$	1.546	1.546	1.545	1.548	1.550	1.547	1.550
$r(C_1\text{-}C_{8a})$	1.435	1.438	1.434	1.434	1.434	1.435	1.421
$r(N_8\text{-}C_{8a})$	1.318	1.317	1.318	1.317	1.317	1.318	1.319
$r(C_1\text{-}C_2)$	1.391	1.395	1.394	1.389	1.387	1.387	1.379
$r(C_2\text{-}C_3)$	1.403	1.399	1.399	1.405	1.406	1.404	1.414
$\varphi(C_{8a}\text{-}N_{4a}\text{-}B)$	124.2	124.3	124.2	124.2	124.2	124.2	124.1
$\varphi_e(N_{4a}\text{-}C_3\text{-}C_2)$	110.7	110.9	110.9	110.7	110.7	110.7	110.6
$\varphi(N_{4a}\text{-}B\text{-}N_{4b})$	104.7	104.7	104.6	104.6	104.6	104.7	104.6
$\varphi(C_1\text{-}C_2\text{-}C_3)$	107.6	107.5	107.1	107.4	107.4	107.4	106.8
$WI(C_1\text{-}C_{1\text{-}Sub})$	1.07	1.12	1.12	1.07	1.06	1.06	-
$WI(C_3\text{-}H_{1\text{-}Sub})$	0.90	0.90	0.90	0.90	0.90	0.90	0.90

[a] r - bond lengths in Å, φ – bond angles in degrees, χ - dihedral angle in degrees, WI - Wiberg bond indexes; [b] for **D2** – the average value of the deviation from the substituent's location in the plane, $\lvert\chi_1\rvert$=13.1 °, $\lvert\chi_2\rvert$=168.2 °. Natural charges and Wiberg bond indexes are given in Tables S10 and S14.

Table S5. Rotation angles χ_1 (°) for aza-BODIPY derivatives according to PBE/6-31G(d) calculations.

Substituent	phenyl	2-thiophenyl	2-furanyl	3-pyridinyl	4-pyridinyl	2-pyridinyl
once substituted at position 1	17.5	0.0	0.0	19.5	16.7	0.0
doble substituted at positions 1, 7	22.0	13.8	7.8	24.3	22.3	11.5

Table S6. Relative energies (kJ mol^{-1}) of conformers II[a] in relation to conformer I for **A1-6.**

	A1	A2	A3	A4	A5	A6
CAM-B3LYP [b]	8.52	5.93	5.25	9.06	5.69	7.80
PBE [b]	9.49	0.92	-0.79	7.06	7.38	0.74

[a] See Figure 3; [b] see Computational details.

Table S7. Natural charges at atoms calculated[a] in the framework of natural population analysis for **A1-7**.

	**A1**	**A2**	**A3**	**A4**	**A5**	**A6**	**A7**
Symmetry	C_2	C_1	C_2	C_2	C_2	C_2	C_2
N_{4a}	-0.63	-0.62	-0.62	-0.63	-0.63	-0.63	-0.62
C_{8a}	0.34	0.36	0.35	0.34	0.34	0.35	0.31
C_1	-0.02	-0.04	-0.05	-0.02	-0.04	-0.04	-0.20
C_2	-0.26	-0.29	-0.29	-0.28	-0.24	-0.26	-0.31
C_3	0.26	0.26	0.26	0.26	0.26	0.26	0.26
N_{4b}	-0.63	-0.62	-0.62	-0.63	-0.62	-0.63	-0.62
C_{8b}	0.34	0.34	0.35	0.34	0.35	0.35	0.31
C_7	-0.01	-0.03	-0.05	-0.03	-0.03	-0.05	-0.20
C_6	-0.29	-0.30	-0.29	-0.25	-0.27	-0.23	-0.31
C_5	0.26	0.26	0.26	0.26	0.27	0.25	0.26
B	1.35	1.31	1.31	1.35	1.35	1.35	1.31
N_m	-0.39	-0.40	-0.40	-0.40	-0.39	-0.40	-0.38
F	-0.56	-0.57	-0.57	-0.56	-0.56	-0.56	-0.56
F	-0.56	-0.57	-0.57	-0.56	-0.56	-0.56	-0.56

[a] CAM-B3LYP/6-31+G(d,p)

Table S8. Natural charges at atoms calculated[a] in the framework of natural population analysis for **B1-7**.

	**B1**	**B2**	**B3**	**B4**	**B5**	**B6**	**B7**
Symmetry	C_2	C_1	C_2	C_2	C_2	C_2	C_{2v}
N_{4a}	-0.64	-0.64	-0.63	-0.63	-0.63	-0.63	-0.63
C_{8a}	0.32	0.33	0.34	0.33	0.33	0.33	0.30
C_1	0.00	-0.03	-0.05	-0.01	-0.02	-0.03	-0.20
C_2	-0.31	-0.31	-0.31	-0.31	-0.30	-0.29	-0.33
C_3	0.33	0.32	0.32	0.33	0.33	0.33	0.33
N_{4b}	-0.64	-0.64	-0.63	-0.63	-0.63	-0.63	-0.63
C_{8b}	0.32	0.33	0.34	0.32	0.33	0.33	0.30
C_7	0.00	-0.03	-0.05	0.00	-0.01	-0.02	-0.20
C_6	-0.32	-0.30	-0.31	-0.31	-0.30	-0.29	-0.33
C_5	0.33	0.32	0.32	0.33	0.33	0.33	0.33
B	1.35	1.35	1.31	1.35	1.35	1.36	1.31
N_m	-0.38	-0.38	-0.38	-0.38	-0.37	-0.38	-0.35
F	-0.56	-0.56	-0.57	-0.56	-0.56	-0.56	-0.57
F	-0.56	-0.56	-0.57	-0.56	-0.56	-0.56	-0.57

[a] CAM-B3LYP/6-31+G(d,p)

Table S9. Natural charges at atoms calculated[a] in the framework of natural population analysis for **C1-7**.

	**C1**	**C2**	**C3**	**C4**	**C5**	**C6**	**C7**
Symmetry	C_1	C_1	C_1	C_1	C_1	C_1	C_s
N_{4a}	-0.62	-0.61	-0.62	-0.62	-0.61	-0.62	-0.61
C_{8a}	0.33	0.34	0.35	0.34	0.34	0.34	0.31
C_1	0.00	-0.05	-0.06	-0.04	-0.02	-0.04	-0.20
C_2	-0.30	-0.30	-0.30	-0.28	-0.28	-0.28	-0.31
C_3	0.29	0.30	0.29	0.29	0.30	0.29	0.30
N_{4b}	-0.62	-0.62	-0.62	-0.62	-0.61	-0.62	-0.61
C_{8b}	0.33	0.33	0.35	0.34	0.34	0.34	0.31
C_7	0.00	-0.05	-0.05	-0.03	-0.02	-0.03	-0.20
C_6	-0.30	-0.30	-0.30	-0.28	-0.29	-0.28	-0.31
C_5	0.30	0.29	0.29	0.30	0.30	0.30	0.30
B	1.35	1.32	1.32	1.35	1.35	1.35	1.31
N_m	-0.37	-0.38	-0.38	-0.39	-0.38	-0.39	-0.36
F	-0.55	-0.57	-0.57	-0.55	-0.55	-0.55	-0.56
F	-0.56	-0.56	-0.56	-0.54	-0.55	-0.54	-0.56

[a] CAM-B3LYP/6-31+G(d,p)

Table S10. Natural charges at atoms calculated[a] in the framework of natural population analysis for **D1-7**.

	**D1**	**D2**	**D3**	**D4**	**D5**	**D6**	**D7**
Symmetry	C_2	C_1	C_2	C_2	C_2	C_2	C_{2v}
N_{4a}	-0.63	-0.62	-0.62	-0.62	-0.62	-0.62	-0.61
C_{8a}	0.32	0.33	0.34	0.32	0.33	0.33	0.29
C_1	0.00	-0.02	-0.05	-0.01	-0.01	-0.05	-0.20
C_2	-0.32	-0.32	-0.31	-0.31	-0.30	-0.23	-0.34
C_3	0.10	0.10	0.09	0.10	0.10	0.08	0.11
N_{4b}	-0.62	-0.63	-0.62	-0.62	-0.62	-0.62	-0.61
C_{8b}	0.32	0.32	0.34	0.33	0.33	0.33	0.29
C_7	0.00	-0.02	-0.05	-0.01	-0.02	-0.05	-0.20
C_6	-0.32	-0.33	-0.31	-0.31	-0.30	-0.24	-0.34
C_5	0.10	0.10	0.09	0.10	0.10	0.08	0.11
B	1.35	1.31	1.31	1.35	1.35	1.35	1.31
N_m	-0.35	-0.34	-0.37	-0.36	-0.35	-0.36	-0.33
F	-0.56	-0.57	-0.57	-0.56	-0.56	-0.56	-0.56
F	-0.56	-0.57	-0.57	-0.56	-0.56	-0.56	-0.56

[a] CAM-B3LYP/6-31+G(d,p)

Table S11. Wiberg bond indexes calculated[a] for **A1-7**.

	**A1**	**A2**	**A3**	**A4**	**A5**	**A6**	**A7**
Symmetry	C_2	C_1	C_2	C_2	C_2	C_2	C_2
C_{8a}-N_{4a}	1.08	1.08	1.08	1.08	1.08	1.08	1.07
C_3-N_{4a}	1.25	1.24	1.24	1.25	1.26	1.25	1.27
N_{4a}-B	0.62	0.63	0.63	0.62	0.62	0.62	0.63
C_1-C_{8a}	1.18	1.16	1.17	1.18	1.18	1.18	1.22
N_m-C_{4a}	1.39	1.40	1.39	1.39	1.39	1.39	1.39
C_1-C_2	1.47	1.44	1.43	1.48	1.48	1.48	1.55
C_2-C_3	1.29	1.30	1.31	1.29	1.28	1.29	1.27
B-F	0.65	0.67	0.68	0.65	0.65	0.65	0.68

[a] CAM-B3LYP/6-31+G(d,p)

Table S12. Wiberg bond indexes calculated[a] for **B1-7**.

	**B1**	**B2**	**B3**	**B4**	**B5**	**B6**	**B7**
Symmetry	C_2	C_1	C_2	C_2	C_2	C_2	C_{2v}
C_{8a}-N_{4a}	1.06	1.07	1.07	1.07	1.07	1.07	1.05
C_3-N_{4a}	1.29	1.27	1.28	1.30	1.30	1.29	1.32
N_{4a}-B	0.62	0.62	0.63	0.62	0.62	0.61	0.63
C_1-C_{8a}	1.18	1.18	1.17	1.18	1.18	1.18	1.22
N_m-C_{4a}	1.40	1.39	1.40	1.40	1.40	1.40	1.40
C_1-C_2	1.45	1.40	1.41	1.45	1.46	1.46	1.53
C_2-C_3	1.33	1.36	1.35	1.32	1.31	1.32	1.30
B-F	0.66	0.65	0.68	0.66	0.66	0.65	0.68

[a] CAM-B3LYP/6-31+G(d,p)

Table S13. Wiberg bond indexes calculated[a] for **C1-7**.

	**C1**	**C2**	**C3**	**C4**	**C5**	**C6**	**C7**
Symmetry	C_1	C_1	C_1	C_1	C_1	C_1	C_s
C_{8a}-N_{4a}	1.07	1.07	1.08	1.08	1.08	1.08	1.06
C_3-N_{4a}	1.28	1.27	1.27	1.28	1.29	1.28	1.30
N_{4a}-B	0.62	0.62	0.62	0.62	0.61	0.62	0.63
C_1-C_{8a}	1.18	1.16	1.17	1.18	1.18	1.18	1.22
N_m-C_{4a}	1.40	1.40	1.40	1.39	1.40	1.39	1.40
C_1-C_2	1.46	1.44	1.43	1.47	1.48	1.47	1.55
C_2-C_3	1.31	1.32	1.33	1.30	1.30	1.30	1.28
B-F	0.66	0.65	0.65	0.64	0.64	0.64	0.66

[a] CAM-B3LYP/6-31+G(d,p)

Table S14. Wiberg bond indexes calculated[a] for **D1-7**.

	**D1**	**D2**	**D3**	**D4**	**D5**	**D6**	**D7**
Symmetry	C_2	C_1	C_2	C_2	C_2	C_2	C_{2v}
C_{8a}-N_{4a}	1.07	1.07	1.08	1.07	1.07	1.07	1.06
C_3-N_{4a}	1.32	1.31	1.30	1.33	1.34	1.33	1.35
N_{4a}-B	0.61	0.63	0.63	0.61	0.61	0.61	0.62
C_1-C_{8a}	1.18	1.17	1.17	1.18	1.18	1.18	1.22
N_m-C_{4a}	1.40	1.41	1.40	1.40	1.40	1.40	1.40
C_1-C_2	1.44	1.42	1.40	1.45	1.46	1.45	1.54
C_2-C_3	1.36	1.38	1.39	1.35	1.35	1.35	1.33
B-F	0.67	0.69	0.69	0.67	0.67	0.67	0.69

[a] CAM-B3LYP/6-31+G(d,p)

Table S15. Contributions of aza-BODIPY core and substituent groups R_1-R_4 to frontier MOs of **A1-7**, **B1-B7**, **C1-C7**, **D1-7**.

Molecule	HOMO contributors, %					LUMO contributors, %				
	Core	R_1	R_2	R_3	R_4	Core	R_1	R_2	R_3	R_4
**A1**	61.5	3.2	3.2	16.1	16.1	72.8	4.1	4.1	9.5	9.5
**A2**	57.9	6.8	7.1	13.9	14.2	68.8	6.9	7.0	8.9	8.4
**A3**	56.8	8.2	8.2	13.4	13.4	69.1	6.7	6.7	8.8	8.8
**A4**	61.4	2.3	2.3	17.0	17.0	72.7	4.1	4.1	9.6	9.6
**A5**	61.3	1.6	1.6	17.7	17.7	72.5	4.2	4.2	9.5	9.5
**A6**	61.3	2.3	2.3	17.1	17.1	71.9	4.8	4.8	9.3	9.3
**A7**	64.5	0.0	0.0	17.7	17.7	78.1	0.1	0.1	10.9	10.9
**B1**	81.5	7.2	7.2	2.1	2.1	85.8	5.6	5.6	1.5	1.5
**B2**	66.8	15.4	15.4	1.6	1.6	79.0	9.2	9.2	1.3	1.3
**B3**	63.5	16.8	16.8	1.5	1.5	80.3	8.5	8.5	1.4	1.4
**B4**	84.9	5.3	5.3	2.3	2.3	85.8	5.6	5.6	1.5	1.5
**B5**	84.9	5.3	5.3	2.3	2.3	85.8	5.6	5.6	1.5	1.5
**B6**	84.9	5.3	5.3	2.3	2.3	84.1	6.5	6.5	1.4	1.4
**B7**	95.1	0.0	0.0	2.4	2.4	96.3	0.1	0.1	1.8	1.8
**C1**	70.9	4.4	4.8	9.8	10.1	78.4	4.7	4.9	6.0	6.0
**C2**	63.0	11.5	10.2	7.5	7.9	73.5	8.1	7.5	5.3	5.6
**C3**	60.7	12.9	12.0	7.0	7.3	74.7	7.2	7.4	5.3	5.4
**C4**	71.5	3.1	3.4	10.7	11.2	75.7	4.7	4.9	5.8	6.0
**C5**	72.4	2.0	2.2	11.5	11.9	78.3	4.9	5.1	5.9	5.8
**C6**	71.8	3.3	3.1	10.6	11.1	77.6	5.6	5.4	5.6	5.8
**C7**	76.9	0.0	0.0	11.5	11.5	84.2	0.1	0.1	7.4	7.4
**D1**	77.4	11.2	11.2	0.0	0.0	88.6	5.6	5.6	0.1	0.1
**D2**	59.6	20.2	20.2	0.0	0.0	82.5	8.7	8.7	0.1	0.1
**D3**	55.5	22.2	22.2	0.0	0.0	82.8	8.5	8.5	0.1	0.1
**D4**	84.0	7.9	7.9	0.1	0.1	88.6	5.5	5.5	0.1	0.1
**D5**	90.9	4.5	4.5	0.1	0.1	88.8	5.6	5.6	0.1	0.1
**D6**	84.1	7.9	7.9	0.1	0.1	87.6	6.1	6.1	0.1	0.1
**D7**	99.8	0.0	0.0	0.1	0.1	99.7	0.1	0.1	0.1	0.1

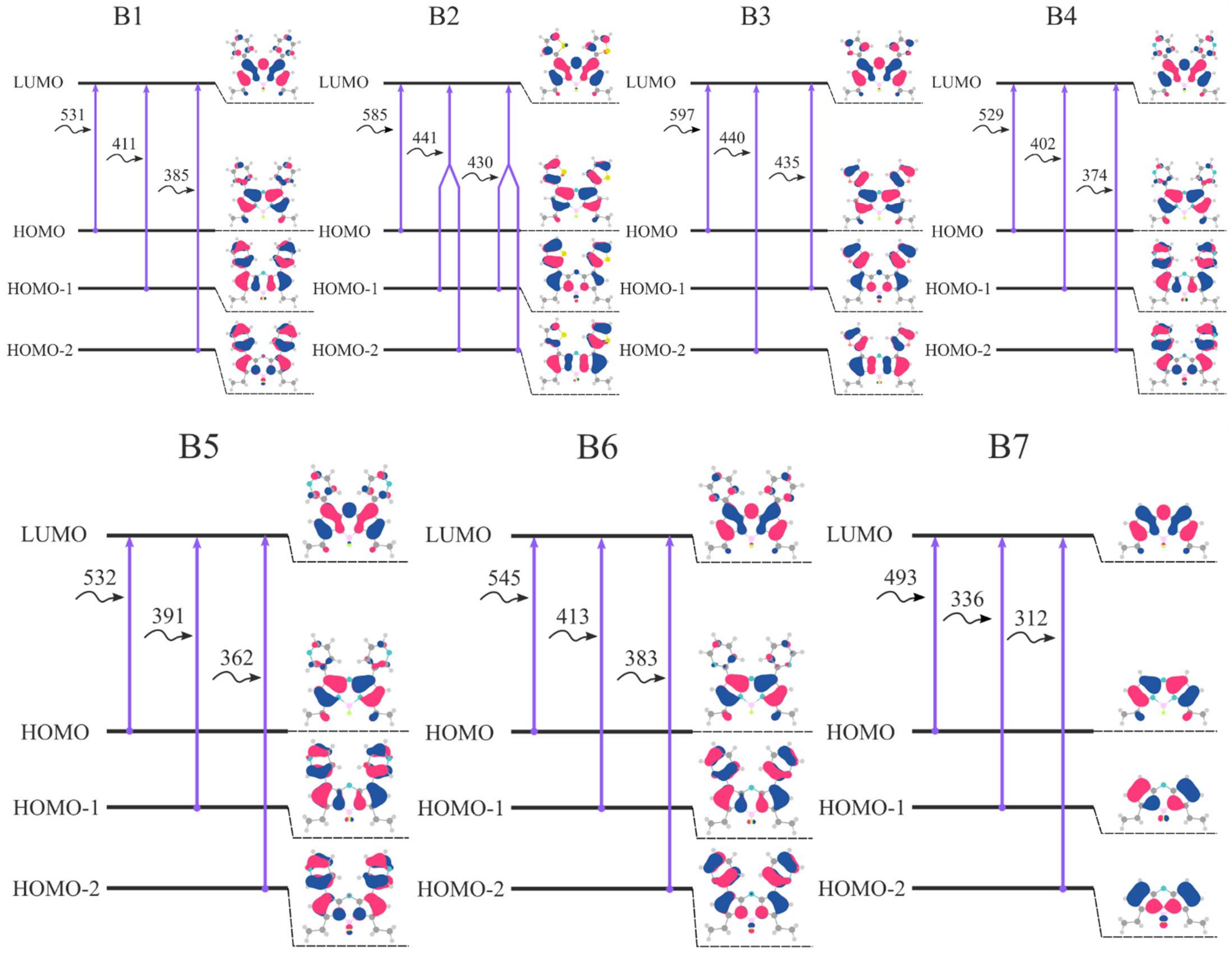

Figure S5. Visual representation of the first three transitions for molecules **B1-7** calculated at CAM-B3LYP/6-31+G(d,p) level. Isosurface cutoff is 0.03.

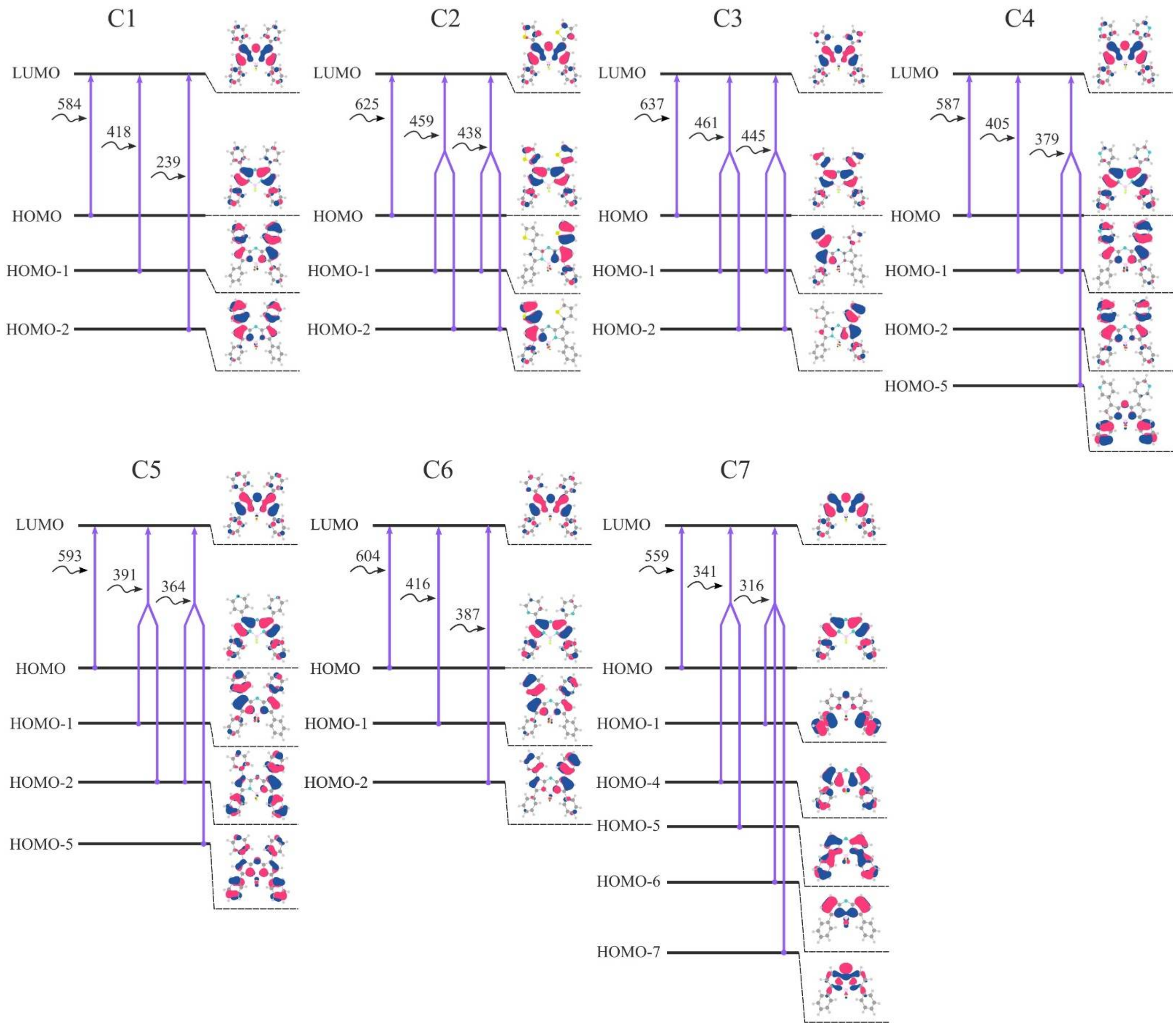

Figure S6. Visual representation of the first three transitions for molecules **C1-7** calculated at CAM-B3LYP/6-31+G(d,p) level. Isosurface cutoff is 0.03.

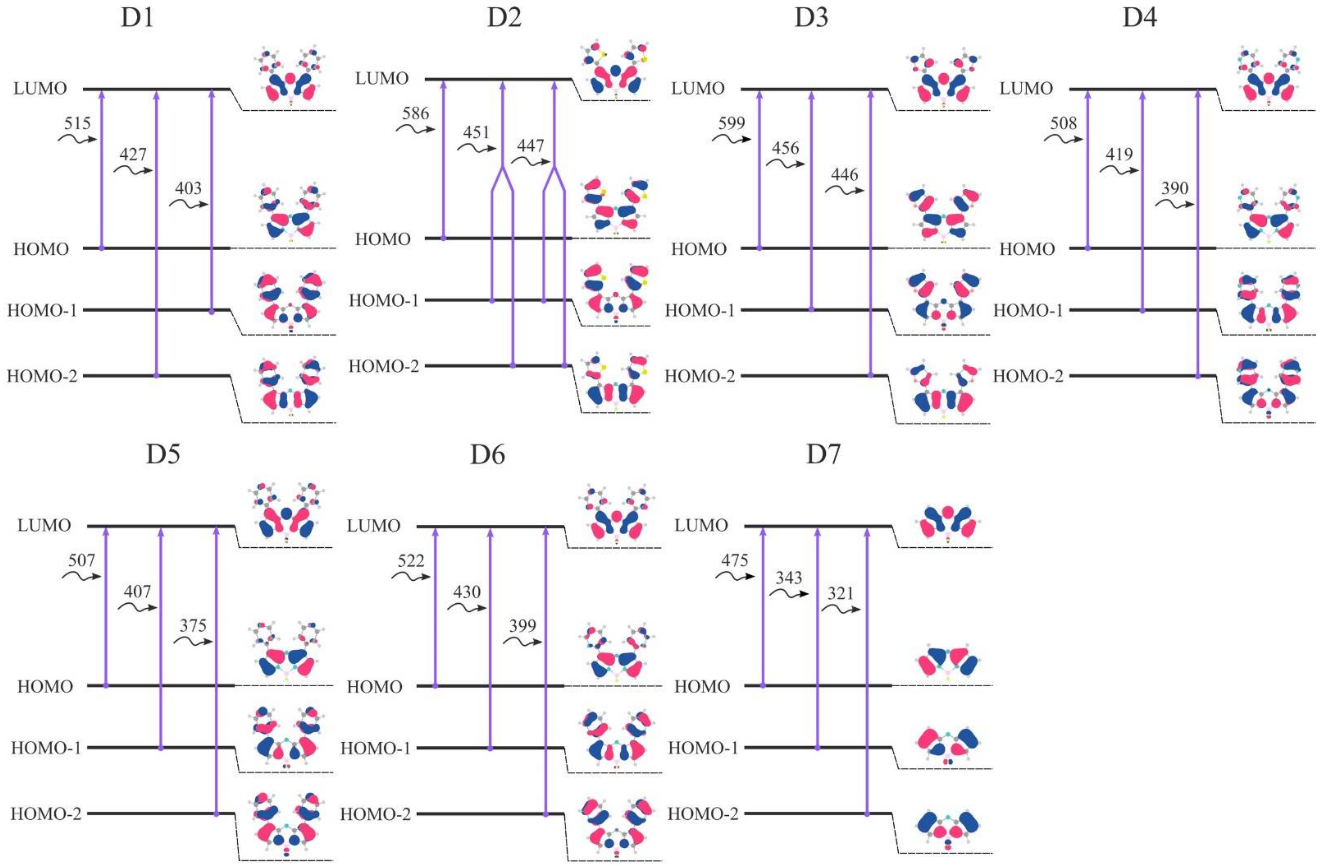

Figure S7. Visual representation of the first three transitions for molecules **D1-7** calculated at CAM-B3LYP/6-31+G(d,p) level. Isosurface cutoff is 0.03.

Table S16. Calculated (CAM-B3LYP/6-31+G(d,p)) composition of five lowest excited states and corresponding oscillator strengths.

Compound	Transition and state symmetry	Main determinant contributions (CI expansion coefficient squared), %	Energy, eV	Energy, cm^{-1}	Wavelength, nm	Oscillator Strength
A1	1 (B)	HOMO -> LUMO, 98.4	1.92	15486	647	0.918
	2 (B)	HOMO-1 -> LUMO, 91.4	3.00	24197	413	0.013
	3 (A)	HOMO-2 -> LUMO, 87.5<br>HOMO-3 -> LUMO, 7.2	3.17	25568	391	0.536
	4 (A)	HOMO-2 -> LUMO, 8.3<br>HOMO-3 -> LUMO, 85.9	3.71	29923	334	0.388
	5 (B)	HOMO-5 -> LUMO, 43.5<br>HOMO-11 -> LUMO, 50.7	3.84	30972	323	0.150
A2	1 (A)	HOMO -> LUMO, 98.4	1.83	14760	679	0.886
	2 (A)	HOMO-1 -> LUMO, 83.9<br>HOMO-2 -> LUMO, 10.0	2.69	21697	460	0.005
	3 (A)	HOMO-1 -> LUMO, 10.1<br>HOMO-2 -> LUMO, 83.7	2.83	22826	438	0.570
	4 (A)	HOMO-3 -> LUMO, 90.7	3.67	29601	338	0.376
	5 (A)	HOMO-4 -> LUMO, 48.8<br>HOMO-5 -> LUMO, 35.5<br>HOMO-11 -> LUMO, 11.5	3.81	30730	326	0.051
A3	1 (B)	HOMO -> LUMO, 98.6	1.79	14437	693	0.879
	2 (B)	HOMO-1 -> LUMO, 94.3	2.66	21455	466	0.006
	3 (A)	HOMO-2 -> LUMO, 94.6	2.78	22422	446	0.582
	4 (A)	HOMO-3 -> LUMO, 91.6	3.67	29601	338	0.373
	5 (B)	HOMO-4 -> LUMO, 28.1<br>HOMO-6 -> LUMO, 46.1<br>HOMO-11 -> LUMO, 14.7	4.01	32343	309	0.002
A4	1 (B)	HOMO -> LUMO, 98.4	1.89	15244	657	0.913
	2 (B)	HOMO-2 -> LUMO, 90.1	3.10	25003	400	0.024
	3 (A)	HOMO-1 -> LUMO, 74.8<br>HOMO-3 -> LUMO, 18.9	3.28	26455	378	0.551
	4 (A)	HOMO-1 -> LUMO, 21.0<br>HOMO-3 -> LUMO,73.1	3.66	29520	339	0.341
	5 (B)	HOMO-6 -> LUMO, 30.6<br>HOMO-11 -> LUMO, 45.3<br>HOMO-13 -> LUMO, 11.9	3.92	31617	317	0.006
A5	1 (B)	HOMO -> LUMO, 98.4	1.86	15002	666	0.896
	2 (B)	HOMO-2 -> LUMO, 88.9	3.22	25971	385	0.029
	3 (A)	HOMO-1 -> LUMO, 49.0<br>HOMO-3 -> LUMO, 28.9<br>HOMO-5 -> LUMO, 15.2	3.42	27584	363	0.569
	4 (A)	HOMO-1 -> LUMO, 46.7<br>HOMO-3 -> LUMO, 38.7<br>HOMO-5 -> LUMO, 7.9	3.61	29117	343	0.294
	5 (B)	HOMO-7 -> LUMO, 28.2<br>HOMO-8 -> LUMO, 11.5<br>HOMO-11 -> LUMO, 50.5	3.83	30891	324	0.003
A6	1 (B)	HOMO -> LUMO, 98.5	1.83	14760	676	0.892
	2 (B)	HOMO-2 -> LUMO, 91.4	3.02	24358	411	0.020
	3 (A)	HOMO-1 -> LUMO, 77.5<br>HOMO-3 -> LUMO, 17.1	3.21	25891	387	0.539
	4 (A)	HOMO-1 -> LUMO, 18.5<br>HOMO-3 -> LUMO, 76.2	3.61	29117	343	0.377
	5 (B)	HOMO-4 -> LUMO, 8.4<br>HOMO-10 -> LUMO, 7.9<br>HOMO-11 -> LUMO, 55.3<br>HOMO-12 -> LUMO, 18.4	3.92	31617	317	0.009
A7	1 (B)	HOMO -> LUMO, 98.5	1.98	15970	627	0.875

	2 (B)	HOMO-2 -> LUMO, 7.2<br>HOMO-4 -> LUMO, 80.2<br>HOMO-5 -> LUMO, 9.8	3.63	29278	341	0.043
	3 (A)	HOMO-1 -> LUMO, 88.0<br>HOMO-6 -> LUMO, 5.8	3.72	30004	333	0.325
	4 (A)	HOMO-1 -> LUMO, 8.6<br>HOMO-3 -> LUMO, 35.1<br>HOMO-6 -> LUMO, 53.8	3.95	31859	314	0.114
	5 (B)	HOMO-2 -> LUMO, 14.1<br>HOMO-7 -> LUMO, 80.2	4.06	32746	306	0.019
B1	1 (B)	HOMO -> LUMO, 99.0	2.33	18793	531	0.824
	2 (B)	HOMO-1 -> LUMO, 93.5	3.02	24358	411	0.002
	3 (A)	HOMO-2 -> LUMO, 94.4	3.22	25971	385	0.322
	4 (B)	HOMO-4 -> LUMO, 28.2<br>HOMO-7 -> LUMO, 66.9	3.84	30972	323	0.005
	5 (A)	HOMO-3 -> LUMO, 95.3	4.09	32988	303	0.000
B2	1 (A)	HOMO -> LUMO, 98.8	2.12	17099	585	0.731
	2 (A)	HOMO-1 -> LUMO, 10.7<br>HOMO-2 -> LUMO, 84.1	2.81	22664	441	0.044
	3 (A)	HOMO-1 -> LUMO, 85.3<br>HOMO-2 -> LUMO, 10.6	2.88	23229	430	0.357
	4 (A)	HOMO-3 -> LUMO, 45.9<br>HOMO-4 -> LUMO, 21.5<br>HOMO-7 -> LUMO, 28.6	3.85	31053	322	0.030
	5 (A)	HOMO-3 -> LUMO, 51.4<br>HOMO-4 -> LUMO, 22.8<br>HOMO-7 -> LUMO, 22.2	3.97	32020	312	0.001
B3	1 (B)	HOMO -> LUMO, 99.2	2.08	16776	597	0.723
	2 (B)	HOMO-2 -> LUMO, 94.9	2.81	22664	440	0.049
	3 (A)	HOMO-1 -> LUMO, 96.4	2.85	22987	435	0.377
	4 (B)	HOMO-5 -> LUMO, 5.2<br>HOMO-7 -> LUMO, 90.4	4.01	32343	309	0.002
	5 (B)	HOMO-3 -> LUMO, 58.1<br>HOMO-5 -> LUMO, 34.5	4.70	37908	264	0.001
B4	1 (B)	HOMO -> LUMO, 98.9	2.34	18873	529	0.818
	2 (B)	HOMO-1 -> LUMO, 93.7	3.08	24842	402	0.012
	3 (A)	HOMO-2 -> LUMO, 93.7	3.32	26778	374	0.293
	4 (B)	HOMO-3 -> LUMO, 28.3<br>HOMO-7 -> LUMO, 34.7<br>HOMO-9 -> LUMO, 29.9	3.87	31214	320	0.001
	5 (A)	HOMO-3 -> LUMO+1, 5.1<br>HOMO-4 -> LUMO, 85.2	4.44	35811	279	0.000
B5	1 (B)	HOMO -> LUMO, 98.8	2.33	18793	532	0.790
	2 (B)	HOMO-1 -> LUMO, 95.6	3.17	25568	391	0.027
	3 (A)	HOMO-2 -> LUMO, 95.8	3.43	27665	362	0.261
	4 (B)	HOMO-4 -> LUMO, 27.0<br>HOMO-5 -> LUMO, 12.7<br>HOMO-7 -> LUMO, 60.5	3.79	30569	327	0.005
	5 (A)	HOMO-3 -> LUMO, 94.7	4.14	33392	299	0.000
B6	1 (B)	HOMO -> LUMO, 99.0	2.27	18309	545	0.794
	2 (B)	HOMO-1 -> LUMO, 94.3	3.00	24197	413	0.009
	3 (A)	HOMO-2 -> LUMO, 94.4	3.24	26132	383	0.308
	4 (B)	HOMO-5 -> LUMO, 8.3<br>HOMO-7 -> LUMO, 21.1<br>HOMO-8 -> LUMO, 64.8	3.86	31133	321	0.002
	5 (A)	HOMO-3 -> LUMO+2, 2.4<br>HOMO-3 -> LUMO, 87.3<br>HOMO-4 -> LUMO+1, 8.1	4.13	33311	301	0.002
B7	1 (B2)	HOMO -> LUMO, 97.8	2.51	20245	493	0.696
	2 (B2)	HOMO-1 -> LUMO, 97.1	3.69	29763	336	0.105
	3 (A1)	HOMO-2 -> LUMO, 98.3	3.97	32021	312	0.055

	4 (B1)	HOMO-3 -> LUMO, 97.4	4.01	32344	309	0.002
	5 (A2)	HOMO -> LUMO+3, 9.5<br>HOMO -> LUMO+1, 80.9	5.58	45007	222	0.000
C1	1 (A)	HOMO -> LUMO, 98.3	2.12	17099	584	0.902
	2 (A)	HOMO-1 -> LUMO, 89.4	2.97	23955	418	0.011
	3 (A)	HOMO-2 -> LUMO, 90.4	3.16	25487	392	0.446
	4 (A)	HOMO-4 -> LUMO, 38.0<br>HOMO-11 -> LUMO, 51.3	3.78	30488	328	0.033
	5 (A)	HOMO-3 -> LUMO, 95.2	4.00	32262	310	0.004
C2	1 (A)	HOMO -> LUMO, 98.4	1.98	15970	625	0.842
	2 (A)	HOMO-1 -> LUMO, 75.7<br>HOMO-2 -> LUMO, 18.1	2.70	21777	459	0.036
	3 (A)	HOMO-1 -> LUMO, 18.8<br>HOMO-2 -> LUMO, 76.5	2.83	22826	438	0.476
	4 (A)	HOMO-3 -> LUMO, 51.4<br>HOMO-6 -> LUMO, 19.0<br>HOMO-7 -> LUMO, 13.7<br>HOMO-11 -> LUMO, 6.1	3.82	30811	325	0.040
	5 (A)	HOMO-3 -> LUMO, 37.3<br>HOMO-6 -> LUMO, 16.1<br>HOMO-7 -> LUMO, 6.7<br>HOMO-11 -> LUMO, 29.4	3.85	31053	322	0.099
C3	1 (A)	HOMO -> LUMO, 98.7	1.95	15728	637	0.832
	2 (A)	HOMO-1 -> LUMO, 35.5<br>HOMO-2 -> LUMO, 58.4	2.69	21697	461	0.029
	3 (A)	HOMO-1 -> LUMO, 59.8<br>HOMO-2 -> LUMO, 36.0	2.79	22503	445	0.505
	4 (A)	HOMO-4 -> LUMO, 20.4<br>HOMO-5 -> LUMO, 33.9<br>HOMO-10 -> LUMO, 7.1<br>HOMO-11 -> LUMO, 26.8	3.90	31456	318	0.190
	5 (A)	HOMO-3 -> LUMO, 71.5<br>HOMO-4 -> LUMO, 17.8	4.08	32908	304	0.006
C4	1 (A)	HOMO -> LUMO, 98.2	2.11	17018	587	0.895
	2 (A)	HOMO-1 -> LUMO, 89.6	3.06	24681	405	0.022
	3 (A)	HOMO-2 -> LUMO, 86.8<br>HOMO-5 -> LUMO, 5.0	3.27	26375	379	0.420
	4 (A)	HOMO-4 -> LUMO, 9.4<br>HOMO-5 -> LUMO, 20.2<br>HOMO-6 -> LUMO, 15.4<br>HOMO-11 -> LUMO, 26.7<br>HOMO-13 -> LUMO, 14.6	3.81	30730	325	0.083
	5 (A)	HOMO-4 -> LUMO, 9.0<br>HOMO-5 -> LUMO, 43.9<br>HOMO-6 -> LUMO, 12.2<br>HOMO-11 -> LUMO, 10.7	4.03	32504	308	0.321
C5	1 (A)	HOMO -> LUMO, 98.2	2.09	16857	593	0.871
	2 (A)	HOMO-1 -> LUMO, 87.6<br>HOMO-2 -> LUMO, 5.1	3.17	25568	391	0.034
	3 (A)	HOMO-2 -> LUMO, 57.7<br>HOMO-5 -> LUMO, 33.3	3.41	27504	364	0.380
	4 (A)	HOMO-2 -> LUMO, 8.9<br>HOMO-5 -> LUMO, 17.8<br>HOMO-7 -> LUMO, 12.5<br>HOMO-8 -> LUMO, 5.7<br>HOMO-11 -> LUMO, 41.2	3.73	30085	332	0.068
	5 (A)	HOMO-2 -> LUMO, 22.2<br>HOMO-4 -> LUMO, 9.1<br>HOMO-5 -> LUMO, 27.4<br>HOMO-7 -> LUMO, 17.0<br>HOMO-11 -> LUMO, 12.6	3.96	31940	313	0.329
C6	1 (A)	HOMO -> LUMO, 98.3	2.05	16535	604	0.871

	2 (A)	HOMO-1 -> LUMO, 89.9	2.98	24036	416	0.023
	3 (A)	HOMO-2 -> LUMO, 87.7	3.21	25891	387	0.422
	4 (A)	HOMO-4 -> LUMO, 12.9<br>HOMO-5 -> LUMO, 24.4<br>HOMO-11 -> LUMO, 8.2<br>HOMO-12 -> LUMO, 30.3<br>HOMO-13 -> LUMO, 11.1	3.79	30569	327	0.130
	5 (A)	HOMO-4 -> LUMO, 8.0<br>HOMO-5 -> LUMO, 42.5<br>HOMO-12 -> LUMO, 22.2	3.99	32182	311	0.274
C7	1 (A′′)	HOMO -> LUMO, 98.0	2.22	17906	559	0.829
	2 (A′′)	HOMO-4 -> LUMO, 81.4<br>HOMO-5 -> LUMO, 15.9	3.64	29359	341	0.077
	3 (A′)	HOMO-1 -> LUMO, 38.7<br>HOMO-6 -> LUMO, 13.2<br>HOMO-7 -> LUMO, 42.7	3.93	31698	316	0.067
	4 (A′)	HOMO-6 -> LUMO, 80.3<br>HOMO-7 -> LUMO, 16.2	3.95	31859	314	0.065
	5 (A′′)	HOMO-2 -> LUMO, 93.8	4.12	33230	301	0.015
D1	1 (B)	HOMO -> LUMO, 99.1	2.41	19438	515	0.768
	2 (B)	HOMO-2 -> LUMO, 93.7	2.90	23390	427	0.003
	3 (A)	HOMO-1 -> LUMO, 94.7	3.08	24842	403	0.290
	4 (B)	HOMO-4 -> LUMO, 36.4<br>HOMO-7 -> LUMO, 58.4	3.72	30004	333	0.007
	5 (A)	HOMO-3 -> LUMO, 96.4	3.90	31456	318	0.000
D2	1 (A)	HOMO -> LUMO, 98.5	2.12	17099	586	0.628
	2 (A)	HOMO-1 -> LUMO, 89.2<br>HOMO-2 -> LUMO, 7.0	2.75	22180	451	0.300
	3 (A)	HOMO-1 -> LUMO, 7.1<br>HOMO-2 -> LUMO, 87.5	2.78	22422	447	0.113
	4 (A)	HOMO-3 -> LUMO, 64.3<br>HOMO-4 -> LUMO, 19.4<br>HOMO-7 -> LUMO, 13.3	3.71	29923	334	0.036
	5 (A)	HOMO-3 -> LUMO, 31.8<br>HOMO-4 -> LUMO, 54.5<br>HOMO-7 -> LUMO, 10.6	3.83	30891	324	0.010
D3	1 (B)	HOMO -> LUMO, 98.9	2.07	16696	599	0.624
	2 (A)	HOMO-1 -> LUMO, 96.6	2.72	21938	456	0.334
	3 (B)	HOMO-2 -> LUMO, 94.9	2.78	22422	446	0.102
	4 (B)	HOMO-7 -> LUMO, 94.5	3.92	31617	316	0.002
	5 (B)	HOMO-3 -> LUMO, 71.7<br>HOMO-5 -> LUMO, 22.5	4.65	37505	267	0.001

A7	1 (B)	HOMO -> LUMO, 98.5	1.98	15970	627	0.875
	2 (B)	HOMO-2 -> LUMO, 7.2<br>HOMO-4 -> LUMO, 80.2<br>HOMO-5 -> LUMO, 9.8	3.63	29278	341	0.043
	3 (A)	HOMO-1 -> LUMO, 88.0<br>HOMO-6 -> LUMO, 5.8	3.72	30004	333	0.325
	4 (A)	HOMO-1 -> LUMO, 8.6<br>HOMO-3 -> LUMO, 35.1<br>HOMO-6 -> LUMO, 53.8	3.95	31859	314	0.114
	5 (B)	HOMO-2 -> LUMO, 14.1<br>HOMO-7 -> LUMO, 80.2	4.06	32746	306	0.019
B1	1 (B)	HOMO -> LUMO, 98.9	2.36	19035	525	0.820
	2 (B)	HOMO-1 -> LUMO, 93.5	3.06	24681	405	0.005
	3 (A)	HOMO-2 -> LUMO, 94.5	3.26	26294	381	0.330
	4 (B)	HOMO-4 -> LUMO, 27.0<br>HOMO-7 -> LUMO, 67.8	3.86	31133	321	0.006
	5 (A)	HOMO-3 -> LUMO, 95.0	4.12	33230	301	0.000
B2	1 (A)	HOMO -> LUMO, 98.8	2.15	17341	576	0.736
	2 (A)	HOMO-1 -> LUMO, 11.1<br>HOMO-2 -> LUMO, 83.7	2.83	22826	438	0.035
	3 (A)	HOMO-1 -> LUMO, 85.0<br>HOMO-2 -> LUMO, 11.1	2.91	23471	426	0.366
	4 (A)	HOMO-3 -> LUMO, 43.5<br>HOMO-4 -> LUMO, 20.0<br>HOMO-7 -> LUMO, 31.8	3.87	31214	320	0.027
	5 (A)	HOMO-3 -> LUMO, 53.5<br>HOMO-4 -> LUMO, 21.5<br>HOMO-7 -> LUMO, 21.0	4.00	32262	310	0.001
B3	1 (B)	HOMO -> LUMO, 99.1	2.12	17099	586	0.045
	2 (B)	HOMO-2 -> LUMO, 95.1	2.83	22826	438	0.382
	3 (A)	HOMO-1 -> LUMO, 96.5	2.88	23229	431	0.003
	4 (B)	HOMO-5 -> LUMO, 9.1<br>HOMO-6 -> LUMO, 94.6	4.03	32504	308	0.002
	5 (B)	HOMO-3 -> LUMO, 56.3<br>HOMO-2 -> LUMO, 35.3	4.75	38312	261	0.202
B4	1 (B)	HOMO -> LUMO, 98.8	2.37	19116	524	0.813
	2 (B)	HOMO-1 -> LUMO, 93.7	3.13	25245	396	0.018
	3 (A)	HOMO-2 -> LUMO, 93.7	3.36	27020	369	0.303
	4 (B)	HOMO-3 -> LUMO, 27.6<br>HOMO-7 -> LUMO, 41.8<br>HOMO-8 -> LUMO, 23.0	3.90	31456	318	0.000
	5 (A)	HOMO-3 -> LUMO+3, 2.6<br>HOMO-3 -> LUMO+1, 5.4<br>HOMO-4 -> LUMO, 84.6	4.48	36134	277	0.000
B5	1 (B)	HOMO -> LUMO, 98.8	2.35	18954	527	0.785
	2 (B)	HOMO-1 -> LUMO, 95.6	3.23	26052	384	0.034
	3 (A)	HOMO-2 -> LUMO, 95.8	3.48	28068	357	0.272
	4 (B)	HOMO-4 -> LUMO, 20.5<br>HOMO-5 -> LUMO, 12.3<br>HOMO-7 -> LUMO, 61.1	3.82	30811	325	0.005
	5 (A)	HOMO-3 -> LUMO, 94.4	4.19	33795	296	0.000
B6	1 (B)	HOMO -> LUMO, 98.9	2.30	18551	539	0.789
	2 (B)	HOMO-1 -> LUMO, 94.3	3.05	24600	407	0.014
	3 (A)	HOMO-2 -> LUMO, 94.6	3.28	26455	378	0.319
	4 (B)	HOMO-5 -> LUMO, 8.3<br>HOMO-7 -> LUMO, 37.9<br>HOMO-8 -> LUMO, 47.8	3.89	31375	319	0.002
	5 (A)	HOMO-3 -> LUMO, 86.6<br>HOMO-4 -> LUMO+1, 8.5	4.16	33553	298	0.002

B7	1 (A′′)	HOMO -> LUMO, 98.0	2.53	20406	491	0.695
	2 (A′′)	HOMO-1 -> LUMO, 97.4	3.76	30327	330	0.108
	3 (A′)	HOMO-2 -> LUMO, 63.4<br>HOMO-3 -> LUMO, 34.4	4.03	32504	307	0.039
	4 (A′)	HOMO-2 -> LUMO, 34.6<br>HOMO-3 -> LUMO, 62.6	4.03	32504	307	0.025
	5 (A′′)	HOMO -> LUMO+3, 13.9<br>HOMO -> LUMO+1, 76.6	5.59	45087	222	0.000
C1	1 (A)	HOMO -> LUMO, 98.3	2.12	17099	584	0.902
	2 (A)	HOMO-1 -> LUMO, 89.4	2.97	23955	418	0.011
	3 (A)	HOMO-2 -> LUMO, 90.4	3.16	25487	392	0.446
	4 (A)	HOMO-4 -> LUMO, 38.0<br>HOMO-11 -> LUMO, 51.3	3.78	30488	328	0.033
	5 (A)	HOMO-3 -> LUMO, 95.2	4.00	32262	310	0.004
C2	1 (A)	HOMO -> LUMO, 98.4	1.98	15970	625	0.842
	2 (A)	HOMO-1 -> LUMO, 75.7<br>HOMO-2 -> LUMO, 18.1	2.70	21777	459	0.036
	3 (A)	HOMO-1 -> LUMO, 18.8<br>HOMO-2 -> LUMO, 76.5	2.83	22826	438	0.476
	4 (A)	HOMO-3 -> LUMO, 51.4<br>HOMO-6 -> LUMO, 19.0<br>HOMO-7 -> LUMO, 13.7<br>HOMO-11 -> LUMO, 6.1	3.82	30811	325	0.040
	5 (A)	HOMO-3 -> LUMO, 37.3<br>HOMO-6 -> LUMO, 16.1<br>HOMO-7 -> LUMO, 6.7<br>HOMO-11 -> LUMO, 29.4	3.85	31053	322	0.099
C3	1 (A)	HOMO -> LUMO, 98.7	1.95	15728	637	0.832
	2 (A)	HOMO-1 -> LUMO, 35.5<br>HOMO-2 -> LUMO, 58.4	2.69	21697	461	0.029
	3 (A)	HOMO-1 -> LUMO, 59.8<br>HOMO-2 -> LUMO, 36.0	2.79	22503	445	0.505
	4 (A)	HOMO-4 -> LUMO, 20.4<br>HOMO-5 -> LUMO, 33.9<br>HOMO-10 -> LUMO, 7.1<br>HOMO-11 -> LUMO, 26.8	3.90	31456	318	0.190
	5 (A)	HOMO-3 -> LUMO, 71.5<br>HOMO-4 -> LUMO, 17.8	4.08	32908	304	0.006
C4	1 (A)	HOMO -> LUMO, 98.2	2.11	17018	587	0.895
	2 (A)	HOMO-1 -> LUMO, 89.6	3.06	24681	405	0.022
	3 (A)	HOMO-2 -> LUMO, 86.8<br>HOMO-5 -> LUMO, 5.0	3.27	26375	379	0.420
	4 (A)	HOMO-4 -> LUMO, 9.4<br>HOMO-5 -> LUMO, 20.2<br>HOMO-6 -> LUMO, 15.4<br>HOMO-11 -> LUMO, 26.7<br>HOMO-13 -> LUMO, 14.6	3.81	30730	325	0.083
	5 (A)	HOMO-4 -> LUMO, 9.0<br>HOMO-5 -> LUMO, 43.9<br>HOMO-6 -> LUMO, 12.2<br>HOMO-11 -> LUMO, 10.7	4.03	32504	308	0.321

C5	1 (A)	HOMO -> LUMO, 98.2	2.09	16857	593	0.871
	2 (A)	HOMO-1 -> LUMO, 87.6<br>HOMO-2 -> LUMO, 5.1	3.17	25568	391	0.034
	3 (A)	HOMO-2 -> LUMO, 57.7<br>HOMO-5 -> LUMO, 33.3	3.41	27504	364	0.380
	4 (A)	HOMO-2 -> LUMO, 8.9<br>HOMO-5 -> LUMO, 17.8<br>HOMO-7 -> LUMO, 12.5<br>HOMO-8 -> LUMO, 5.7<br>HOMO-11 -> LUMO, 41.2	3.73	30085	332	0.068
	5 (A)	HOMO-2 -> LUMO, 22.2<br>HOMO-4 -> LUMO, 9.1<br>HOMO-5 -> LUMO, 27.4<br>HOMO-7 -> LUMO, 17.0<br>HOMO-11 -> LUMO, 12.6	3.96	31940	313	0.329
C6	1 (A)	HOMO -> LUMO, 98.3	2.05	16535	604	0.871
	2 (A)	HOMO-1 -> LUMO, 89.9	2.98	24036	416	0.023
	3 (A)	HOMO-2 -> LUMO, 87.7	3.21	25891	387	0.422
	4 (A)	HOMO-4 -> LUMO, 12.9<br>HOMO-5 -> LUMO, 24.4<br>HOMO-11 -> LUMO, 8.2<br>HOMO-12 -> LUMO, 30.3<br>HOMO-13 -> LUMO, 11.1	3.79	30569	327	0.130
	5 (A)	HOMO-4 -> LUMO, 8.0<br>HOMO-5 -> LUMO, 42.5<br>HOMO-12 -> LUMO, 22.2	3.99	32182	311	0.274
C7	1 (A′′)	HOMO -> LUMO, 98.0	2.22	17906	559	0.829
	2 (A′′)	HOMO-4 -> LUMO, 81.4<br>HOMO-5 -> LUMO, 15.9	3.64	29359	341	0.077
	3 (A′)	HOMO-1 -> LUMO, 38.7<br>HOMO-6 -> LUMO, 13.2<br>HOMO-7 -> LUMO, 42.7	3.93	31698	316	0.067
	4 (A′)	HOMO-6 -> LUMO, 80.3<br>HOMO-7 -> LUMO, 16.2	3.95	31859	314	0.065
	5 (A′′)	HOMO-2 -> LUMO, 93.8	4.12	33230	301	0.015
D1	1 (B)	HOMO -> LUMO, 99.1	2.41	19438	515	0.768
	2 (B)	HOMO-2 -> LUMO, 93.7	2.90	23390	427	0.003
	3 (A)	HOMO-1 -> LUMO, 94.7	3.08	24842	403	0.290
	4 (B)	HOMO-4 -> LUMO, 36.4<br>HOMO-7 -> LUMO, 58.4	3.72	30004	333	0.007
	5 (A)	HOMO-3 -> LUMO, 96.4	3.90	31456	318	0.000
D2	1 (A)	HOMO -> LUMO, 98.5	2.12	17099	586	0.628
	2 (A)	HOMO-1 -> LUMO, 89.2<br>HOMO-2 -> LUMO, 7.0	2.75	22180	451	0.300
	3 (A)	HOMO-1 -> LUMO, 7.1<br>HOMO-2 -> LUMO, 87.5	2.78	22422	447	0.113
	4 (A)	HOMO-3 -> LUMO, 64.3<br>HOMO-4 -> LUMO, 19.4<br>HOMO-7 -> LUMO, 13.3	3.71	29923	334	0.036
	5 (A)	HOMO-3 -> LUMO, 31.8<br>HOMO-4 -> LUMO, 54.5<br>HOMO-7 -> LUMO, 10.6	3.83	30891	324	0.010
D3	1 (B)	HOMO -> LUMO, 98.9	2.07	16696	599	0.624
	2 (A)	HOMO-1 -> LUMO, 96.6	2.72	21938	456	0.334
	3 (B)	HOMO-2 -> LUMO, 94.9	2.78	22422	446	0.102
	4 (B)	HOMO-7 -> LUMO, 94.5	3.92	31617	316	0.002
	5 (B)	HOMO-3 -> LUMO, 71.7<br>HOMO-5 -> LUMO, 22.5	4.65	37505	267	0.001

D4	1 (B)	HOMO -> LUMO, 98.9	2.44	19680	508	0.775
	2 (B)	HOMO-1 -> LUMO, 93.6	2.96	23874	419	0.003
	3 (A)	HOMO-2 -> LUMO, 93.9	3.18	25649	390	0.266
	4 (B)	HOMO-3 -> LUMO, 29.0<br>HOMO-7 -> LUMO, 16.1<br>HOMO-9 -> LUMO, 48.3	3.77	30407	329	0.002
	5 (A)	HOMO-4 -> LUMO, 87.8	4.28	34521	290	0.001
D5	1 (B)	HOMO -> LUMO, 98.6	2.45	19761	507	0.739
	2 (B)	HOMO-1 -> LUMO, 95.3	3.05	24600	407	0.027
	3 (A)	HOMO-2 -> LUMO, 96.0	3.30	26617	375	0.241
	4 (B)	HOMO-4 -> LUMO, 25.0<br>HOMO-5 -> LUMO, 11.8<br>HOMO-7 -> LUMO, 53.8<br>HOMO-8 -> LUMO, 5.5	3.69	29762	336	0.006
	5 (A)	HOMO-3 -> LUMO, 96.1	3.97	32020	312	0.000
D6	1 (B)	HOMO -> LUMO, 99.0	2.38	19196	522	0.751
	2 (B)	HOMO-1 -> LUMO, 94.3	2.88	23229	430	0.002
	3 (A)	HOMO-2 -> LUMO, 94.7	3.10	25003	399	0.279
	4 (B)	HOMO-5 -> LUMO, 6.3<br>HOMO-9 -> LUMO, 84.2	3.77	30407	329	0.002
	5 (A)	HOMO-3 -> LUMO+2, 2.0<br>HOMO-3 -> LUMO+1, 89.6<br>HOMO-4 -> LUMO, 6.6	3.99	32182	311	0.002
D7	1 (B2)	HOMO -> LUMO, 96.4	2.61	21051	475	0.592
	2 (B2)	HOMO-1 -> LUMO, 96.0	3.62	29198	343	0.146
	3 (A1)	HOMO-2 -> LUMO, 98.6	3.86	31133	321	0.062
	4 (B1)	HOMO-3 -> LUMO, 98.1	3.90	31456	318	0.003
	5 (A2)	HOMO-5 -> LUMO, 94.8	5.84	47103	212	0.000

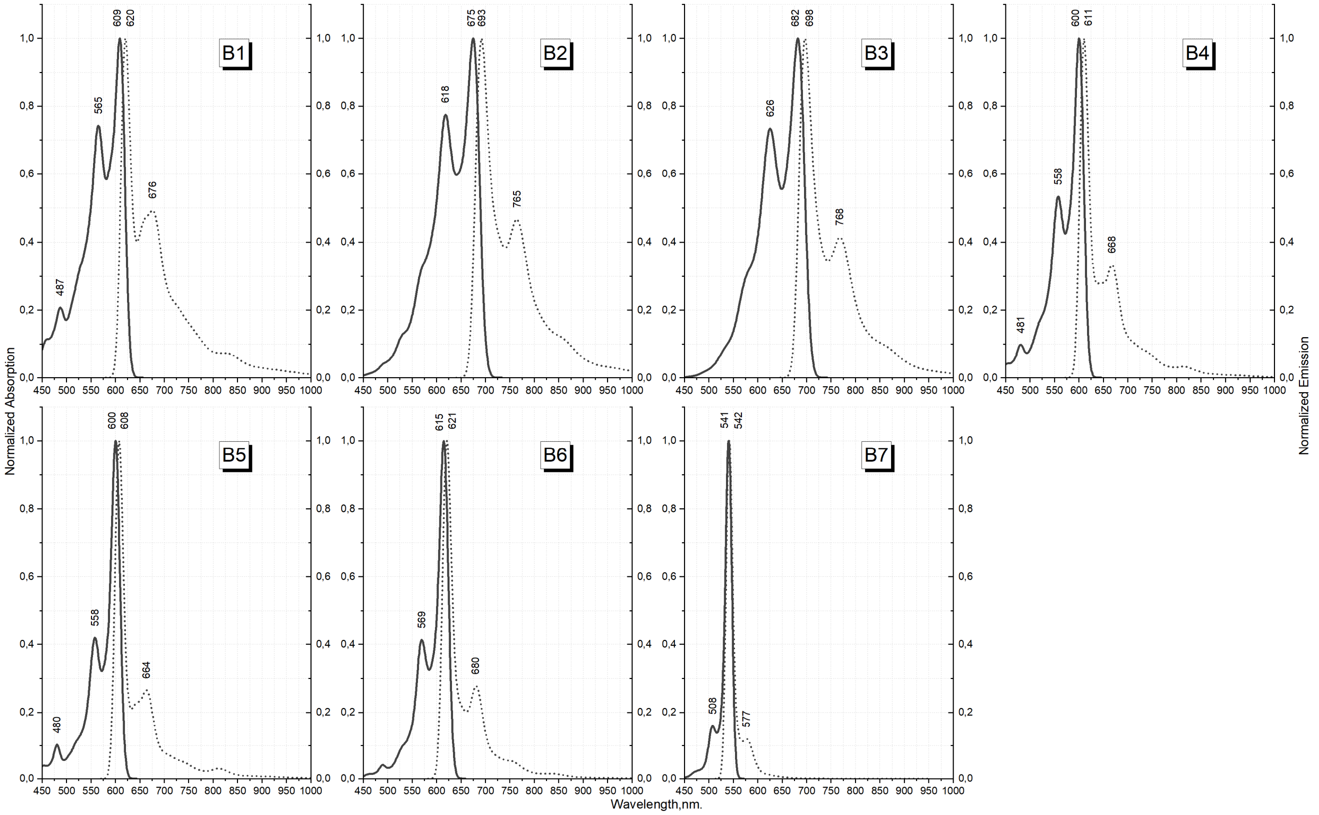

Figure S8. Calculated vibronic (FC, CAM-B3LYP/6-31G(d)) electronic absorption and emission spectra for **B1**-**B7** aza-BODIPY derivatives. Solid lines correspond to absorption and dotted lines correspomd to emission.

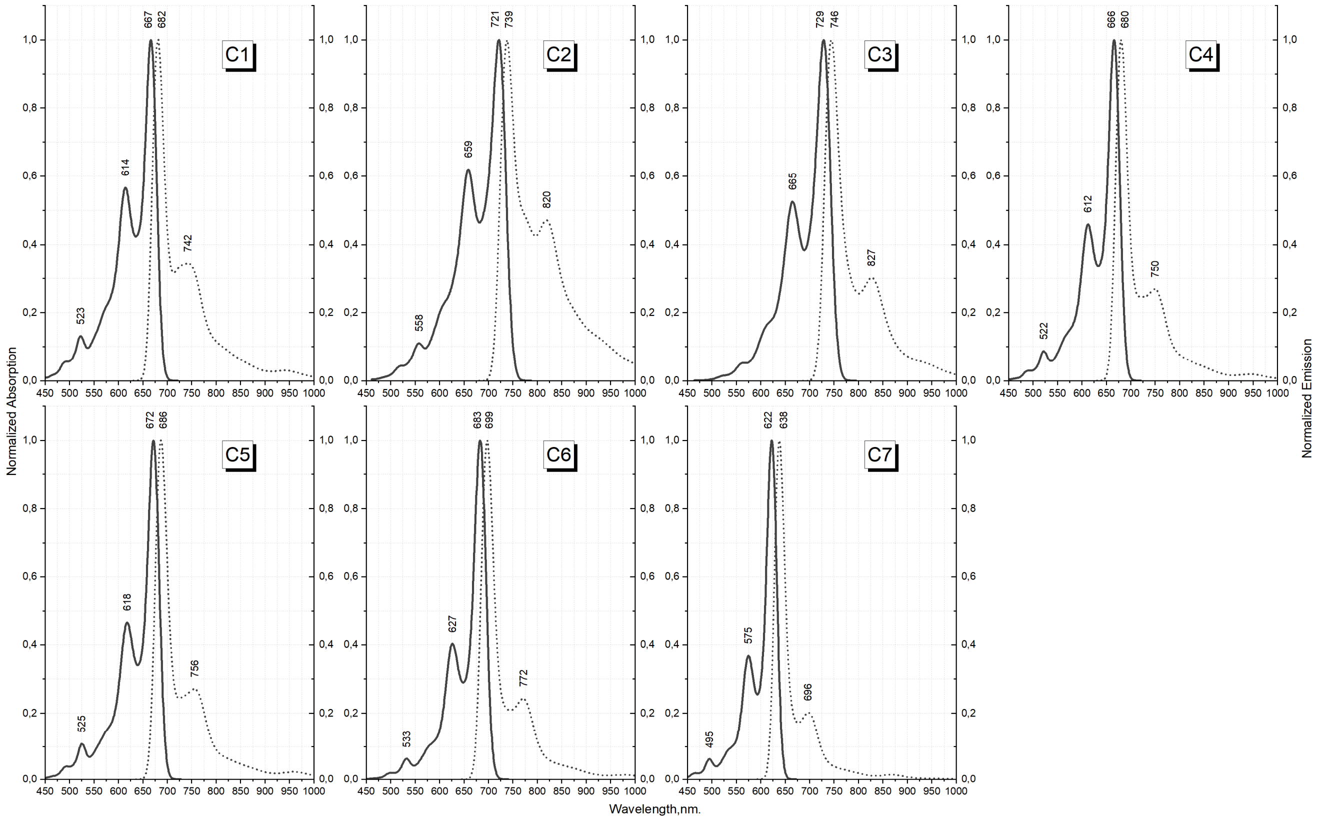

Figure S9. Calculated vibronic (FC, CAM-B3LYP/6-31G(d)) electronic absorption and emission spectra for **C1**-**C7** aza-BODIPY derivatives. Solid lines correspond to absorption and dotted lines correspomd to emission.

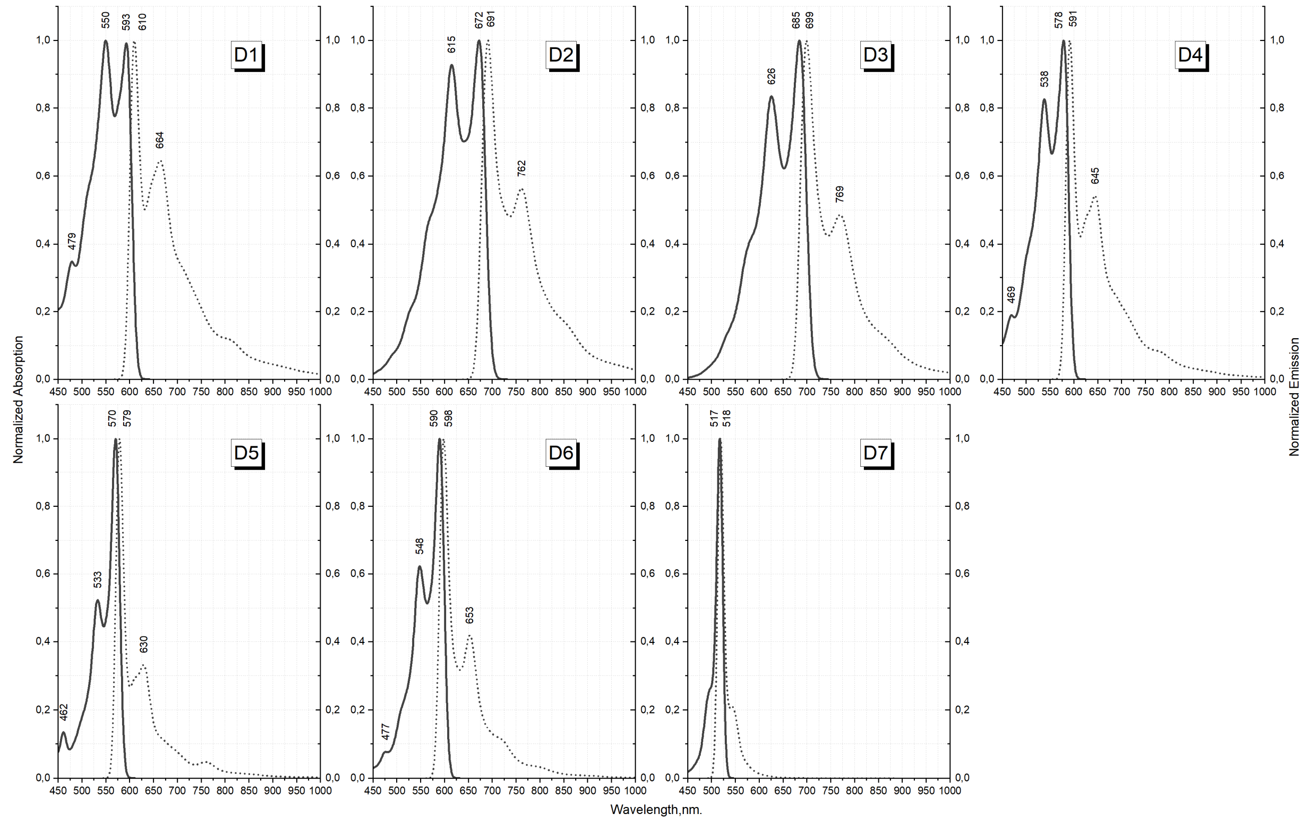

Figure S10. Calculated vibronic (FC, CAM-B3LYP/6-31G(d)) electronic absorption and emission spectra for **D1**-**D7**. Solid lines correspond to absorption and dotted lines correspomd to emission.

Table S17. Experimental [42] and theoretical (CAM-B3LYP/6-31G(d)) bands of absorption and emission spectra.

		**A1**	**A2**	**C1**
Abs main	Exp	710	733	643
	Theor	739	781	672
Abs shoulder	Exp	~645	~660	-
	Theor	671	706	618
Em main	Exp	732	757	673
	Theor	793	795	682
Em shoulder	Exp	-	-	-
	Theor	891	891	742

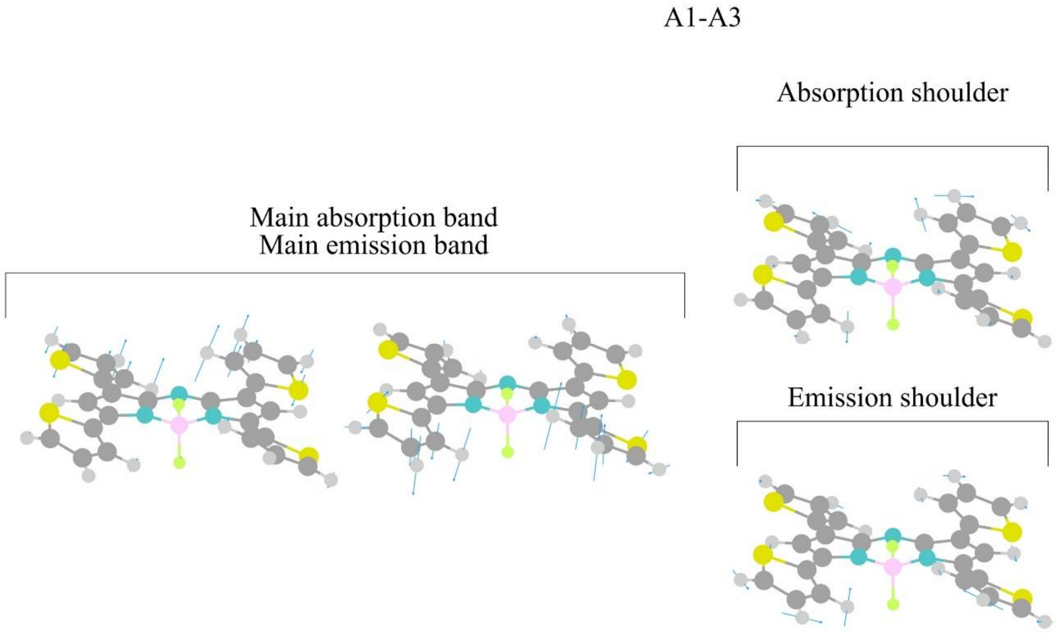

Figure S11. Scaled displacement vectors for some of the main molecular vibrations affecting the line shapes of absorption/emission for **A1**-**A3** (this is shown by the example of **A2**).

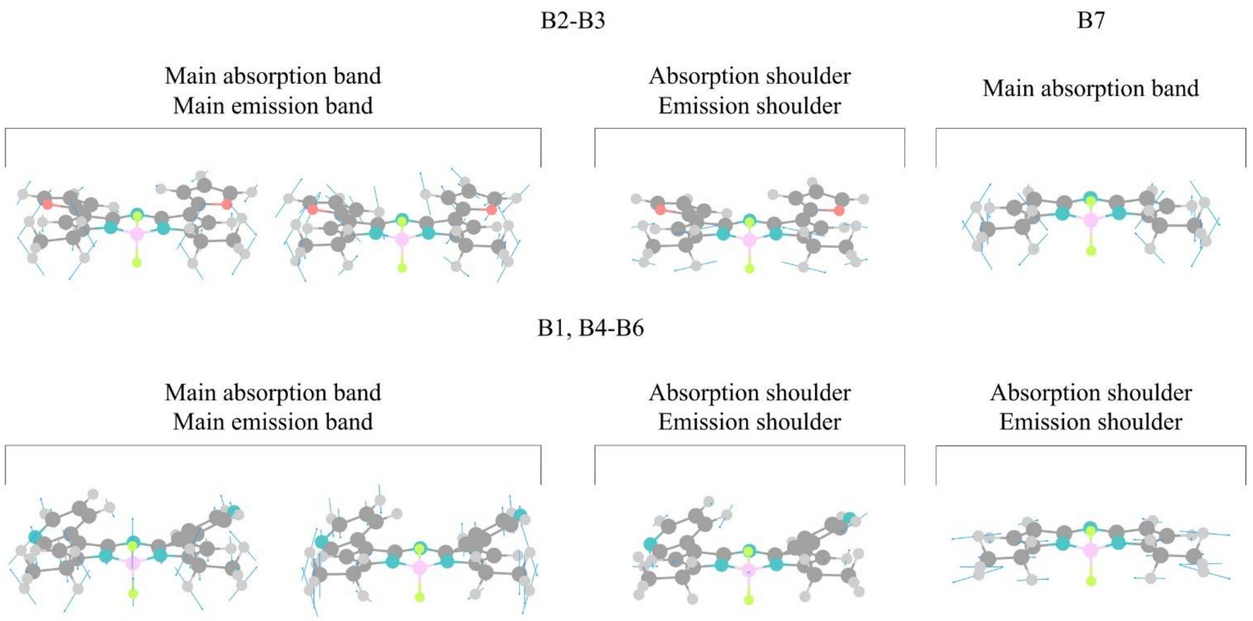

Figure S12. Scaled displacement vectors for some of the main molecular vibrations affecting the lineshapes of absorption/emission for **B1**-**B7** (for **B2**-**B3** – is shown by the example of **B3**, for **B1, B4-B6** – is shown by the example of **B4**).

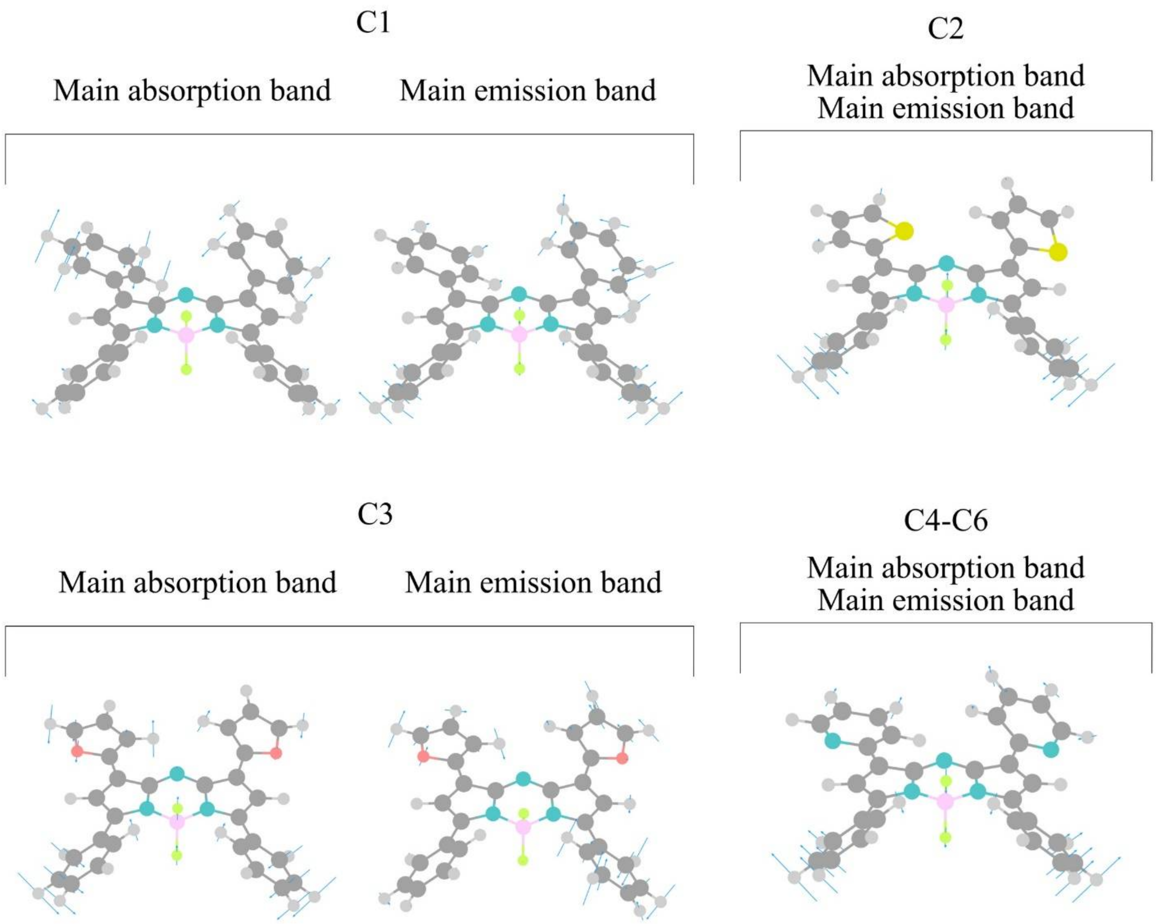

Figure S13. Scaled displacement vectors for some of the main molecular vibrations affecting the lineshapes of absorption/emission for **C1**-**C6** (for **C4**-**D6** – is shown by the example of **D6**).

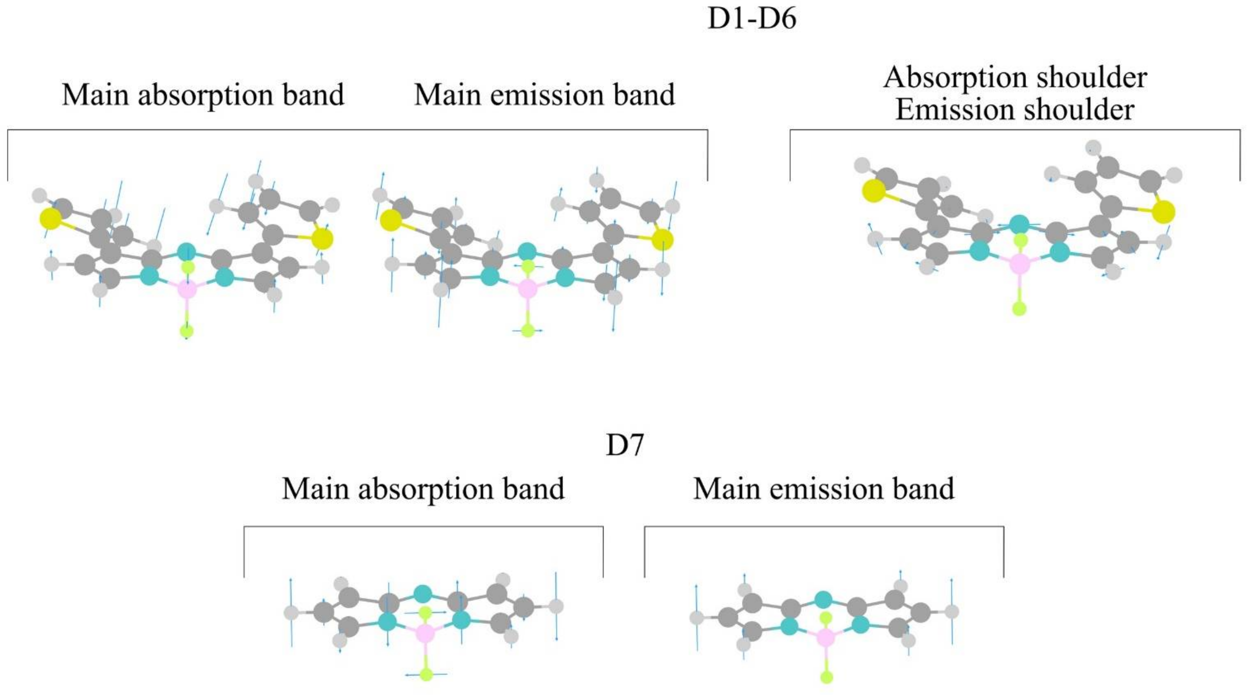

Figure S14. Scaled displacement vectors for some of the main molecular vibrations affecting the lineshapes of absorption/emission for **D1**-**D7** (for **D1**-**D6** – is shown by the example of **D2**).
