# Supplementary material for: Quantum Chemical Study Aimed at Modeling Efficient Aza-BODIPY NIR Dyes: Molecular and Electronic Structure, Absorption, and Emission Spectra
Source: Molecules. 2020 Nov 17;25(22):5361. doi: 10.3390/molecules25225361 (PMC7698449; doi:10.3390/molecules25225361)
Supplement: Supplementary file 1 [file molecules-25-05361-s001.zip › Supplementary Materials 2.pdf]

54

A1 GS CAM-B3LYP/6-31+G(d,p)

N	0.042956000	1.245241000	-0.745274000
C	0.051579000	1.141292000	0.645464000
C	0.043581000	2.454532000	1.213919000
C	0.000000000	3.324307000	0.142262000
C	0.005333000	2.566498000	-1.054274000
N	-0.042956000	-1.245241000	-0.745274000
C	-0.051579000	-1.141292000	0.645464000
C	-0.043581000	-2.454532000	1.213919000
C	0.000000000	-3.324307000	0.142262000
C	-0.005333000	-2.566498000	-1.054274000
B	0.000000000	0.000000000	-1.667456000
N	0.000000000	0.000000000	1.298677000
F	1.144817000	-0.063016000	-2.475746000
F	-1.144817000	0.063016000	-2.475746000
C	0.066462000	2.803583000	2.638261000
H	0.017450000	4.403565000	0.187220000
C	-0.022221000	3.137619000	-2.383960000
C	0.022221000	-3.137619000	-2.383960000
H	-0.017450000	-4.403565000	0.187220000
C	-0.066462000	-2.803583000	2.638261000
C	0.760799000	2.374729000	4.920635000
C	0.733355000	2.007023000	3.579513000
C	-0.541256000	4.338590000	4.416690000
C	-0.760799000	-2.374729000	4.920635000
C	0.124343000	3.539318000	5.344566000

C	-0.569338000	3.974483000	3.075717000
C	-0.733355000	-2.007023000	3.579513000
C	-0.124343000	-3.539318000	5.344566000
H	0.147017000	3.823225000	6.391909000
H	-1.235828000	-1.104025000	3.255150000
H	-0.147017000	-3.823225000	6.391909000
H	-1.044392000	5.244741000	4.738681000
H	-1.286204000	-1.750494000	5.636469000
H	-1.104683000	4.592956000	2.362485000
S	0.516381000	-4.803452000	-2.567738000
C	0.270221000	-4.748083000	-4.263697000
C	-0.168862000	-3.521425000	-4.677223000
C	-0.310029000	-2.600280000	-3.607686000
C	0.310029000	2.600280000	-3.607686000
C	0.168862000	3.521425000	-4.677223000
C	-0.270221000	4.748083000	-4.263697000
S	-0.516381000	4.803452000	-2.567738000
H	0.468653000	-5.623819000	-4.866353000
H	-0.387783000	-3.282009000	-5.710488000
H	-0.662980000	-1.587125000	-3.730498000
H	0.662980000	1.587125000	-3.730498000
H	0.387783000	3.282009000	-5.710488000
H	-0.468653000	5.623819000	-4.866353000
C	0.569338000	-3.974483000	3.075717000
C	0.541256000	-4.338590000	4.416690000
H	1.286204000	1.750494000	5.636469000
H	1.235828000	1.104025000	3.255150000
H	1.104683000	-4.592956000	2.362485000
H	1.044392000	-5.244741000	4.738681000

48

A2 GS CAM-B3LYP/6-31+G(d,p)

N	-0.847570000	-1.067989000	-0.094364000
C	-1.203391000	0.280105000	-0.111842000
C	-2.633211000	0.382914000	-0.153294000
C	-3.103383000	-0.917722000	-0.121841000
C	-1.995516000	-1.794585000	-0.088782000
N	1.502292000	-0.259192000	0.044416000
C	0.950120000	1.018429000	0.039387000
C	2.002944000	1.985030000	0.079828000
C	3.181093000	1.255199000	0.082122000
C	2.855546000	-0.117613000	0.059504000
B	0.628811000	-1.535461000	-0.020138000
N	-0.340289000	1.269576000	-0.039865000
F	0.974891000	-2.278810000	-1.158792000
F	0.806139000	-2.323629000	1.126971000
H	-4.136311000	-1.231512000	-0.165905000
H	4.184617000	1.649941000	0.138542000
C	1.878691000	3.426963000	0.112038000
S	0.395276000	4.255669000	0.490352000
C	2.891484000	4.332915000	-0.109056000
C	1.154874000	5.791597000	0.350123000
C	2.477627000	5.683997000	0.023893000
H	3.900132000	4.036933000	-0.371027000
H	0.579777000	6.693415000	0.510336000
H	3.132291000	6.534839000	-0.119137000
C	-3.410265000	1.601484000	-0.222261000
S	-5.109777000	1.590741000	0.162461000
C	-3.009377000	2.860985000	-0.600974000

C	-5.255702000	3.267895000	-0.187709000
C	-4.065483000	3.811923000	-0.579733000
H	-1.991645000	3.086965000	-0.887606000
H	-6.212160000	3.760658000	-0.079987000
H	-3.945195000	4.854483000	-0.847748000
C	-2.098843000	-3.237942000	-0.054841000
S	-3.622425000	-3.954753000	0.411512000
C	-1.183448000	-4.219406000	-0.363495000
C	-3.009690000	-5.539917000	0.185265000
C	-1.706396000	-5.530905000	-0.227088000
H	-0.179029000	-4.005059000	-0.697026000
H	-3.643498000	-6.395303000	0.375169000
H	-1.137444000	-6.429746000	-0.430096000
C	3.829656000	-1.189534000	0.052317000
S	5.477844000	-0.816848000	-0.391392000
C	3.711179000	-2.523300000	0.372398000
C	5.972235000	-2.439711000	-0.139560000
C	4.935014000	-3.233179000	0.263311000
H	2.783585000	-2.972110000	0.695188000
H	7.002231000	-2.722294000	-0.308927000
H	5.039066000	-4.289415000	0.478813000

48

A3 GS CAM-B3LYP/6-31+G(d,p)

N	0.028888000	1.244725000	-0.455156000
C	0.043277000	1.138761000	0.933870000
C	0.049391000	2.453996000	1.494861000
C	0.000000000	3.329517000	0.425143000
C	-0.008072000	2.568865000	-0.765607000
N	-0.028888000	-1.244725000	-0.455156000

C	-0.043277000	-1.138761000	0.933870000
C	-0.049391000	-2.453996000	1.494861000
C	0.000000000	-3.329517000	0.425143000
C	0.008072000	-2.568865000	-0.765607000
B	0.000000000	0.000000000	-1.377577000
N	0.000000000	0.000000000	1.592666000
F	1.145262000	-0.047943000	-2.186445000
F	-1.145262000	0.047943000	-2.186445000
H	0.006162000	4.407739000	0.479249000
H	-0.006162000	-4.407739000	0.479249000
C	-0.111260000	-2.791749000	2.890419000
O	0.077786000	-4.109733000	3.204011000
C	-0.357420000	-2.083986000	4.034500000
C	-0.043471000	-4.228076000	4.548217000
C	-0.310484000	-3.018922000	5.109819000
H	-0.562806000	-1.026581000	4.086287000
H	0.086113000	-5.220990000	4.949939000
H	-0.459689000	-2.818233000	6.160484000
C	0.111260000	2.791749000	2.890419000
O	-0.077786000	4.109733000	3.204011000
C	0.357420000	2.083986000	4.034500000
C	0.043471000	4.228076000	4.548217000
C	0.310484000	3.018922000	5.109819000
H	0.562806000	1.026581000	4.086287000
H	-0.086113000	5.220990000	4.949939000
H	0.459689000	2.818233000	6.160484000
C	-0.052419000	3.137017000	-2.096472000
C	0.263627000	2.597444000	-3.323418000
C	0.110908000	3.517445000	-4.392435000

C	-0.320842000	4.745390000	-3.975200000
S	-0.546172000	4.803369000	-2.276397000
H	0.613635000	1.583804000	-3.449688000
H	0.316546000	3.276086000	-5.427971000
H	-0.525511000	5.620618000	-4.576503000
C	0.052419000	-3.137017000	-2.096472000
C	-0.263627000	-2.597444000	-3.323418000
C	-0.110908000	-3.517445000	-4.392435000
C	0.320842000	-4.745390000	-3.975200000
S	0.546172000	-4.803369000	-2.276397000
H	-0.613635000	-1.583804000	-3.449688000
H	-0.316546000	-3.276086000	-5.427971000
H	0.525511000	-5.620618000	-4.576503000

52

A4 GS CAM-B3LYP/6-31+G(d,p)

N	0.040939000	1.246371000	-0.739270000
C	0.046425000	1.140538000	0.650003000
C	0.037498000	2.452193000	1.219024000
C	0.000000000	3.325208000	0.151611000
C	0.006508000	2.567786000	-1.047515000
N	-0.040939000	-1.246371000	-0.739270000
C	-0.046425000	-1.140538000	0.650003000
C	-0.037498000	-2.452193000	1.219024000
C	0.000000000	-3.325208000	0.151611000
C	-0.006508000	-2.567786000	-1.047515000
B	0.000000000	0.000000000	-1.662844000
N	0.000000000	0.000000000	1.304710000
F	1.144933000	-0.061118000	-2.468644000
F	-1.144933000	0.061118000	-2.468644000

H	0.019354000	4.404358000	0.197965000
H	-0.019354000	-4.404358000	0.197965000
C	-0.050686000	-2.786321000	2.644791000
C	0.588325000	-3.948677000	3.095930000
C	-0.695465000	-1.995572000	3.601673000
N	0.608642000	-4.341884000	4.368386000
C	-0.675450000	-2.395625000	4.930357000
C	-0.017178000	-3.573942000	5.266370000
H	-1.207787000	-1.088908000	3.303596000
H	-1.169787000	-1.809185000	5.696787000
H	0.009028000	-3.917403000	6.296945000
H	1.122136000	-4.580431000	2.390571000
C	0.050686000	2.786321000	2.644791000
C	-0.588325000	3.948677000	3.095930000
C	0.695465000	1.995572000	3.601673000
N	-0.608642000	4.341884000	4.368386000
C	0.675450000	2.395625000	4.930357000
C	0.017178000	3.573942000	5.266370000
H	-1.122136000	4.580431000	2.390571000
H	1.207787000	1.088908000	3.303596000
H	1.169787000	1.809185000	5.696787000
H	-0.009028000	3.917403000	6.296945000
C	0.017494000	-3.140883000	-2.375147000
C	-0.302796000	-2.599916000	-3.601116000
C	-0.169751000	-3.523506000	-4.668727000
C	0.251281000	-4.755723000	-4.251901000
S	0.489759000	-4.813539000	-2.555640000
H	-0.642250000	-1.582609000	-3.726958000
H	-0.381245000	-3.282410000	-5.703091000

H	0.440375000	-5.634551000	-4.853081000
C	-0.017494000	3.140883000	-2.375147000
C	0.302796000	2.599916000	-3.601116000
C	0.169751000	3.523506000	-4.668727000
C	-0.251281000	4.755723000	-4.251901000
S	-0.489759000	4.813539000	-2.555640000
H	0.642250000	1.582609000	-3.726958000
H	0.381245000	3.282410000	-5.703091000
H	-0.440375000	5.634551000	-4.853081000

52

A5 GS CAM-B3LYP/6-31+G(d,p)

N	0.043511000	1.246820000	-0.742045000
C	0.046037000	1.140824000	0.646842000
C	0.033807000	2.453288000	1.213936000
C	0.000000000	3.325356000	0.147558000
C	0.011652000	2.567433000	-1.052442000
N	-0.043511000	-1.246820000	-0.742045000
C	-0.046037000	-1.140824000	0.646842000
C	-0.033807000	-2.453288000	1.213936000
C	0.000000000	-3.325356000	0.147558000
C	-0.011652000	-2.567433000	-1.052442000
B	0.000000000	0.000000000	-1.665997000
N	0.000000000	0.000000000	1.300850000
F	1.144911000	-0.063442000	-2.470181000
F	-1.144911000	0.063442000	-2.470181000
H	0.016183000	4.404547000	0.194725000
H	-0.016183000	-4.404547000	0.194725000
C	-0.046382000	-2.798229000	2.639851000
C	0.564740000	-3.973770000	3.088173000

C	-0.676232000	-1.997619000	3.597605000
C	0.518829000	-4.289181000	4.439626000
C	-0.665461000	-2.402994000	4.926749000
N	-0.083056000	-3.526099000	5.357505000
H	-1.171182000	-1.077668000	3.314810000
H	0.991952000	-5.198016000	4.801751000
H	-1.153550000	-1.792940000	5.682187000
H	1.085880000	-4.630070000	2.400221000
C	0.046382000	2.798229000	2.639851000
C	-0.564740000	3.973770000	3.088173000
C	0.676232000	1.997619000	3.597605000
C	-0.518829000	4.289181000	4.439626000
C	0.665461000	2.402994000	4.926749000
N	0.083056000	3.526099000	5.357505000
H	-1.085880000	4.630070000	2.400221000
H	1.171182000	1.077668000	3.314810000
H	-0.991952000	5.198016000	4.801751000
H	1.153550000	1.792940000	5.682187000
C	0.007403000	-3.140403000	-2.379435000
C	-0.306291000	-2.595985000	-3.605980000
C	-0.180903000	-3.520325000	-4.673335000
C	0.227671000	-4.756785000	-4.256059000
S	0.463361000	-4.817764000	-2.559949000
H	-0.635925000	-1.575592000	-3.732451000
H	-0.388821000	-3.277095000	-5.707889000
H	0.408824000	-5.637268000	-4.857279000
C	-0.007403000	3.140403000	-2.379435000
C	0.306291000	2.595985000	-3.605980000
C	0.180903000	3.520325000	-4.673335000

C	-0.227671000	4.756785000	-4.256059000
S	-0.463361000	4.817764000	-2.559949000
H	0.635925000	1.575592000	-3.732451000
H	0.388821000	3.277095000	-5.707889000
H	-0.408824000	5.637268000	-4.857279000

52

A6 GS CAM-B3LYP/6-31+G(d,p)

N	0.040226000	1.246077000	-0.721705000
C	0.048366000	1.143066000	0.668143000
C	0.043718000	2.459273000	1.230200000
C	0.000000000	3.327185000	0.161338000
C	0.005394000	2.567071000	-1.034778000
N	-0.040226000	-1.246077000	-0.721705000
C	-0.048366000	-1.143066000	0.668143000
C	-0.043718000	-2.459273000	1.230200000
C	0.000000000	-3.327185000	0.161338000
C	-0.005394000	-2.567071000	-1.034778000
B	0.000000000	0.000000000	-1.643468000
N	0.000000000	0.000000000	1.319356000
F	1.145069000	-0.058567000	-2.450192000
F	-1.145069000	0.058567000	-2.450192000
H	-0.002524000	4.404787000	0.221497000
H	0.002524000	-4.404787000	0.221497000
C	-0.079788000	-2.845502000	2.648515000
N	0.305407000	-4.105671000	2.914902000
C	-0.518199000	-1.975524000	3.651529000
C	0.274539000	-4.523506000	4.178276000
C	-0.543860000	-2.425516000	4.965976000
C	-0.138320000	-3.724826000	5.242572000

H	-0.833211000	-0.972369000	3.397297000
H	0.595854000	-5.547293000	4.352417000
H	-0.882026000	-1.768725000	5.760858000
H	-0.142475000	-4.118423000	6.252789000
C	0.079788000	2.845502000	2.648515000
N	-0.305407000	4.105671000	2.914902000
C	0.518199000	1.975524000	3.651529000
C	-0.274539000	4.523506000	4.178276000
C	0.543860000	2.425516000	4.965976000
C	0.138320000	3.724826000	5.242572000
H	0.833211000	0.972369000	3.397297000
H	-0.595854000	5.547293000	4.352417000
H	0.882026000	1.768725000	5.760858000
H	0.142475000	4.118423000	6.252789000
C	-0.023210000	3.136876000	-2.363575000
C	0.279279000	2.590823000	-3.592021000
C	0.147413000	3.514477000	-4.659569000
C	-0.254986000	4.752236000	-4.240427000
S	-0.476525000	4.815235000	-2.542189000
H	0.605237000	1.569492000	-3.720019000
H	0.346423000	3.269480000	-5.695506000
H	-0.438802000	5.632524000	-4.841118000
C	0.023210000	-3.136876000	-2.363575000
C	-0.279279000	-2.590823000	-3.592021000
C	-0.147413000	-3.514477000	-4.659569000
C	0.254986000	-4.752236000	-4.240427000
S	0.476525000	-4.815235000	-2.542189000
H	-0.605237000	-1.569492000	-3.720019000
H	-0.346423000	-3.269480000	-5.695506000

H	0.438802000	-5.632524000	-4.841118000

34

A7 GS CAM-B3LYP/6-31+G(d,p)

N	0.034541000	1.247174000	1.049691000
C	0.018424000	1.140437000	2.441928000
C	-0.002344000	2.443245000	3.000532000
C	-0.011954000	3.325652000	1.952561000
C	0.010442000	2.568377000	0.743774000
N	-0.034541000	-1.247174000	1.049691000
C	-0.018424000	-1.140437000	2.441928000
C	0.002344000	-2.443245000	3.000532000
C	0.011954000	-3.325652000	1.952561000
C	-0.010442000	-2.568377000	0.743774000
B	0.000000000	0.000000000	0.126016000
N	0.000000000	0.000000000	3.101882000
F	1.145357000	-0.055143000	-0.679826000
F	-1.145357000	0.055143000	-0.679826000
H	-0.015787000	4.404520000	2.007330000
C	0.000000000	3.146213000	-0.581380000
C	0.000000000	-3.146213000	-0.581380000
H	0.015787000	-4.404520000	2.007330000
S	0.415512000	-4.835472000	-0.754030000
C	0.192291000	-4.774997000	-2.452117000
C	-0.182808000	-3.530364000	-2.875902000
C	-0.292399000	-2.599453000	-1.812248000
C	0.292399000	2.599453000	-1.812248000
C	0.182808000	3.530364000	-2.875902000
C	-0.192291000	4.774997000	-2.452117000
S	-0.415512000	4.835472000	-0.754030000

H	0.354965000	-5.661952000	-3.049085000
H	-0.377838000	-3.285367000	-3.912584000
H	-0.595686000	-1.571833000	-1.944955000
H	0.595686000	1.571833000	-1.944955000
H	0.377838000	3.285367000	-3.912584000
H	-0.354965000	5.661952000	-3.049085000
H	-0.008656000	2.656264000	4.059853000
H	0.008656000	-2.656264000	4.059853000

52

B1 GS CAM-B3LYP/6-31+G(d,p)

N	-0.016703000	1.230185000	-1.670809000
C	0.011636000	1.140592000	-0.277960000
C	0.000000000	2.466907000	0.261841000
C	-0.049552000	3.318717000	-0.833405000
C	-0.053814000	2.533881000	-2.004752000
N	0.016703000	-1.230185000	-1.670809000
C	-0.011636000	-1.140592000	-0.277960000
C	0.000000000	-2.466907000	0.261841000
C	0.049552000	-3.318717000	-0.833405000
C	0.053814000	-2.533881000	-2.004752000
B	0.000000000	0.000000000	-2.605023000
N	0.000000000	0.000000000	0.380834000
F	1.141465000	0.015646000	-3.422489000
F	-1.141465000	-0.015646000	-3.422489000
C	0.023886000	2.846612000	1.677763000
H	-0.051269000	4.398145000	-0.812848000
H	0.051269000	-4.398145000	-0.812848000
C	-0.023886000	-2.846612000	1.677763000
C	0.700382000	2.455534000	3.973177000

C	0.674364000	2.060552000	2.639855000
C	-0.568609000	4.427857000	3.421462000
C	-0.700382000	-2.455534000	3.973177000
C	0.079775000	3.638176000	4.369640000
C	-0.595733000	4.036312000	2.088347000
C	-0.674364000	-2.060552000	2.639855000
C	-0.079775000	-3.638176000	4.369640000
H	0.101765000	3.943692000	5.410897000
H	-1.165879000	-1.144205000	2.336989000
H	-0.101765000	-3.943692000	5.410897000
H	-1.059144000	5.348270000	3.721812000
H	-1.213046000	-1.838611000	4.704467000
H	-1.117782000	4.648042000	1.359730000
C	0.595733000	-4.036312000	2.088347000
C	0.568609000	-4.427857000	3.421462000
H	1.213046000	1.838611000	4.704467000
H	1.165879000	1.144205000	2.336989000
H	1.117782000	-4.648042000	1.359730000
H	1.059144000	-5.348270000	3.721812000
C	0.081703000	-2.991976000	-3.426802000
H	-0.789421000	-2.567905000	-3.938758000
H	0.953637000	-2.539595000	-3.912404000
C	0.109428000	-4.507909000	-3.596589000
H	-0.776309000	-4.977691000	-3.159715000
H	0.994658000	-4.948345000	-3.129160000
H	0.131957000	-4.761990000	-4.658778000
C	-0.081703000	2.991976000	-3.426802000
H	0.789421000	2.567905000	-3.938758000
H	-0.953637000	2.539595000	-3.912404000

C	-0.109428000	4.507909000	-3.596589000
H	0.776309000	4.977691000	-3.159715000
H	-0.994658000	4.948345000	-3.129160000
H	-0.131957000	4.761990000	-4.658778000

46

B2 GS CAM-B3LYP/6-31+G(d,p)

N	-1.569374000	-1.429183000	0.023965000
C	-1.279024000	-0.066963000	0.012903000
C	-2.511494000	0.659803000	0.001282000
C	-3.518754000	-0.303467000	0.015556000
C	-2.912086000	-1.570148000	0.028185000
N	0.862638000	-1.780209000	0.006532000
C	0.975374000	-0.389623000	0.041666000
C	2.370391000	-0.052563000	0.055937000
C	3.053051000	-1.263767000	0.009443000
C	2.104877000	-2.302414000	-0.017022000
B	-0.488601000	-2.529173000	0.010293000
N	-0.057003000	0.426922000	0.031219000
F	-0.611139000	-3.332739000	-1.135195000
F	-0.597050000	-3.347254000	1.147060000
H	-4.583346000	-0.126937000	0.000517000
H	4.123878000	-1.402914000	0.014916000
C	2.937637000	1.276274000	0.113575000
S	4.623933000	1.535034000	-0.243769000
C	2.330923000	2.462286000	0.456685000
C	4.488789000	3.220402000	0.068552000
C	3.218540000	3.571559000	0.429112000
H	1.284860000	2.525296000	0.723065000
H	5.354388000	3.860116000	-0.035273000

H	2.925562000	4.585994000	0.670664000
C	-2.688842000	2.095501000	-0.018245000
S	-1.399627000	3.219914000	-0.344930000
C	-3.871667000	2.770399000	0.186878000
C	-2.462474000	4.563952000	-0.202464000
C	-3.742250000	4.179965000	0.084374000
H	-4.803981000	2.268398000	0.415402000
H	-2.082412000	5.567787000	-0.334931000
H	-4.561876000	4.874860000	0.219706000
C	2.348032000	-3.775949000	-0.056351000
H	1.834657000	-4.228481000	0.799348000
H	1.846050000	-4.178614000	-0.943252000
C	3.822209000	-4.168541000	-0.058464000
H	4.333799000	-3.814406000	0.840947000
H	4.344585000	-3.763991000	-0.929966000
H	3.916144000	-5.256413000	-0.088993000
C	-3.568511000	-2.912534000	0.036030000
H	-3.202490000	-3.475728000	-0.829727000
H	-3.209233000	-3.461412000	0.913802000
C	-5.093313000	-2.864910000	0.030130000
H	-5.476155000	-2.360786000	-0.861730000
H	-5.482468000	-2.344268000	0.909687000
H	-5.496425000	-3.880158000	0.038154000

46

B3 GS CAM-B3LYP/6-31+G(d,p)

N	-0.007749000	1.229041000	-1.361053000
C	0.017703000	1.137616000	0.030237000
C	0.035040000	2.466519000	0.561356000
C	0.000000000	3.324074000	-0.533245000

C	-0.021754000	2.535835000	-1.697693000
N	0.007749000	-1.229041000	-1.361053000
C	-0.017703000	-1.137616000	0.030237000
C	-0.035040000	-2.466519000	0.561356000
C	0.000000000	-3.324074000	-0.533245000
C	0.021754000	-2.535835000	-1.697693000
B	0.000000000	0.000000000	-2.295470000
N	0.000000000	0.000000000	0.694939000
F	1.141237000	0.007788000	-3.113915000
F	-1.141237000	-0.007788000	-3.113915000
H	0.002000000	4.402576000	-0.503833000
H	-0.002000000	-4.402576000	-0.503833000
C	-0.090085000	-2.836144000	1.948152000
O	0.051873000	-4.168005000	2.227979000
C	-0.286179000	-2.147569000	3.114143000
C	-0.049233000	-4.313835000	3.570805000
C	-0.256988000	-3.108864000	4.165972000
H	-0.446149000	-1.084270000	3.195024000
H	0.047294000	-5.320531000	3.946766000
H	-0.378415000	-2.927957000	5.223784000
C	0.090085000	2.836144000	1.948152000
O	-0.051873000	4.168005000	2.227979000
C	0.286179000	2.147569000	3.114143000
C	0.049233000	4.313835000	3.570805000
C	0.256988000	3.108864000	4.165972000
H	0.446149000	1.084270000	3.195024000
H	-0.047294000	5.320531000	3.946766000
H	0.378415000	2.927957000	5.223784000
C	-0.051513000	2.989149000	-3.121074000

H	0.804216000	2.541926000	-3.639106000
H	-0.938783000	2.556668000	-3.597043000
C	-0.043581000	4.504711000	-3.295688000
H	0.856348000	4.953869000	-2.866209000
H	-0.914455000	4.968036000	-2.823494000
H	-0.067378000	4.755923000	-4.358526000
C	0.051513000	-2.989149000	-3.121074000
H	-0.804216000	-2.541926000	-3.639106000
H	0.938783000	-2.556668000	-3.597043000
C	0.043581000	-4.504711000	-3.295688000
H	-0.856348000	-4.953869000	-2.866209000
H	0.914455000	-4.968036000	-2.823494000
H	0.067378000	-4.755923000	-4.358526000

50

B4 GS CAM-B3LYP/6-31+G(d,p)

N	0.015003000	1.231198000	-1.662112000
C	-0.009708000	1.139689000	-0.270541000
C	0.000000000	2.464622000	0.269778000
C	0.042326000	3.319521000	-0.821445000
C	0.046761000	2.534628000	-1.995034000
N	-0.015003000	-1.231198000	-1.662112000
C	0.009708000	-1.139689000	-0.270541000
C	0.000000000	-2.464622000	0.269778000
C	-0.042326000	-3.319521000	-0.821445000
C	-0.046761000	-2.534628000	-1.995034000
B	0.000000000	0.000000000	-2.598420000
N	0.000000000	0.000000000	0.389477000
F	1.141653000	-0.013803000	-3.412462000
F	-1.141653000	0.013803000	-3.412462000

H	0.039380000	4.398929000	-0.800481000
H	-0.039380000	-4.398929000	-0.800481000
C	0.015777000	-2.829969000	1.687274000
C	-0.606160000	-4.012025000	2.111740000
C	0.644974000	-2.050505000	2.664199000
N	-0.624874000	-4.432689000	3.375300000
C	0.626181000	-2.479095000	3.983970000
C	-0.014581000	-3.674495000	4.292197000
H	1.145227000	-1.130398000	2.387573000
H	1.108739000	-1.901762000	4.764707000
H	-0.038835000	-4.040600000	5.314989000
H	-1.127395000	-4.636845000	1.391097000
C	-0.015777000	2.829969000	1.687274000
C	0.606160000	4.012025000	2.111740000
C	-0.644974000	2.050505000	2.664199000
N	0.624874000	4.432689000	3.375300000
C	-0.626181000	2.479095000	3.983970000
C	0.014581000	3.674495000	4.292197000
H	1.127395000	4.636845000	1.391097000
H	-1.145227000	1.130398000	2.387573000
H	-1.108739000	1.901762000	4.764707000
H	0.038835000	4.040600000	5.314989000
C	-0.071237000	-2.994849000	-3.416014000
H	-0.946923000	-2.549460000	-3.901444000
H	0.796454000	-2.564283000	-3.928320000
C	-0.087455000	-4.511148000	-3.583862000
H	-0.969708000	-4.957790000	-3.116797000
H	0.801733000	-4.973691000	-3.146358000
H	-0.107513000	-4.766091000	-4.645811000

C	0.071237000	2.994849000	-3.416014000
H	0.946923000	2.549460000	-3.901444000
H	-0.796454000	2.564283000	-3.928320000
C	0.087455000	4.511148000	-3.583862000
H	0.969708000	4.957790000	-3.116797000
H	-0.801733000	4.973691000	-3.146358000
H	0.107513000	4.766091000	-4.645811000

50

B5 GS CAM-B3LYP/6-31+G(d,p)

N	0.011568000	1.231898000	-1.666230000
C	-0.010684000	1.140047000	-0.275029000
C	0.000000000	2.465735000	0.263555000
C	0.037808000	3.319728000	-0.826416000
C	0.039086000	2.534174000	-2.000976000
N	-0.011568000	-1.231898000	-1.666230000
C	0.010684000	-1.140047000	-0.275029000
C	0.000000000	-2.465735000	0.263555000
C	-0.037808000	-3.319728000	-0.826416000
C	-0.039086000	-2.534174000	-2.000976000
B	0.000000000	0.000000000	-2.603476000
N	0.000000000	0.000000000	0.384014000
F	1.141875000	-0.011255000	-3.415125000
F	-1.141875000	0.011255000	-3.415125000
H	0.036031000	4.399124000	-0.804903000
H	-0.036031000	-4.399124000	-0.804903000
C	0.016690000	-2.841281000	1.681096000
C	-0.576920000	-4.035584000	2.103855000
C	0.632436000	-2.051961000	2.657847000
C	-0.529518000	-4.378341000	3.448436000

C	0.624368000	-2.484999000	3.978208000
N	0.058237000	-3.625579000	4.383992000
H	1.115576000	-1.119674000	2.395700000
H	-0.989271000	-5.301844000	3.790140000
H	1.101452000	-1.883578000	4.747502000
H	-1.085827000	-4.685515000	1.400947000
C	-0.016690000	2.841281000	1.681096000
C	0.576920000	4.035584000	2.103855000
C	-0.632436000	2.051961000	2.657847000
C	0.529518000	4.378341000	3.448436000
C	-0.624368000	2.484999000	3.978208000
N	-0.058237000	3.625579000	4.383992000
H	1.085827000	4.685515000	1.400947000
H	-1.115576000	1.119674000	2.395700000
H	0.989271000	5.301844000	3.790140000
H	-1.101452000	1.883578000	4.747502000
C	0.058309000	2.994121000	-3.421880000
H	0.927658000	2.542376000	-3.912652000
H	-0.815799000	2.569589000	-3.928398000
C	0.083754000	4.510299000	-3.589586000
H	0.971685000	4.950904000	-3.127589000
H	-0.799941000	4.978788000	-3.147352000
H	0.099552000	4.764952000	-4.651629000
C	-0.058309000	-2.994121000	-3.421880000
H	-0.927658000	-2.542376000	-3.912652000
H	0.815799000	-2.569589000	-3.928398000
C	-0.083754000	-4.510299000	-3.589586000
H	-0.971685000	-4.950904000	-3.127589000
H	0.799941000	-4.978788000	-3.147352000

H	-0.099552000	-4.764952000	-4.651629000

50

B6 GS CAM-B3LYP/6-31+G(d,p)

N	0.000000000	1.231189000	-1.641508000
C	-0.025313000	1.142140000	-0.249424000
C	-0.034896000	2.471716000	0.283883000
C	0.002212000	3.321894000	-0.808597000
C	0.016917000	2.534100000	-1.979250000
N	0.000000000	-1.231189000	-1.641508000
C	0.025313000	-1.142140000	-0.249424000
C	0.034896000	-2.471716000	0.283883000
C	-0.002212000	-3.321894000	-0.808597000
C	-0.016917000	-2.534100000	-1.979250000
B	0.000000000	0.000000000	-2.576367000
N	0.000000000	0.000000000	0.406859000
F	1.141780000	-0.000615000	-3.390988000
F	-1.141780000	0.000615000	-3.390988000
H	0.006082000	4.399798000	-0.774154000
H	-0.006082000	-4.399798000	-0.774154000
C	0.076552000	-2.885666000	1.693636000
N	-0.281854000	-4.158863000	1.936199000
C	0.492040000	-2.025175000	2.715146000
C	-0.246418000	-4.598665000	3.191906000
C	0.522574000	-2.498145000	4.021244000
C	0.144920000	-3.810996000	4.272464000
H	0.786725000	-1.011040000	2.480678000
H	-0.545819000	-5.632265000	3.346244000
H	0.843143000	-1.848687000	4.829345000
H	0.154195000	-4.222996000	5.275286000

C	-0.076552000	2.885666000	1.693636000
N	0.281854000	4.158863000	1.936199000
C	-0.492040000	2.025175000	2.715146000
C	0.246418000	4.598665000	3.191906000
C	-0.522574000	2.498145000	4.021244000
C	-0.144920000	3.810996000	4.272464000
H	-0.786725000	1.011040000	2.480678000
H	0.545819000	5.632265000	3.346244000
H	-0.843143000	1.848687000	4.829345000
H	-0.154195000	4.222996000	5.275286000
C	-0.043667000	-2.989504000	-3.401715000
H	-0.929978000	-2.557478000	-3.879963000
H	0.813107000	-2.543015000	-3.918737000
C	-0.035670000	-4.505424000	-3.573594000
H	-0.907793000	-4.967645000	-3.102773000
H	0.863008000	-4.954001000	-3.141046000
H	-0.056894000	-4.758135000	-4.636104000
C	0.043667000	2.989504000	-3.401715000
H	0.929978000	2.557478000	-3.879963000
H	-0.813107000	2.543015000	-3.918737000
C	0.035670000	4.505424000	-3.573594000
H	0.907793000	4.967645000	-3.102773000
H	-0.863008000	4.954001000	-3.141046000
H	0.056894000	4.758135000	-4.636104000

32

B7 GS CAM-B3LYP/6-31+G(d,p)

N	0.000000000	1.231838000	0.251175000
C	0.000000000	1.139069000	1.646559000
C	0.000000000	2.454835000	2.176601000

C	0.000000000	3.319632000	1.104976000
C	0.000000000	2.534337000	-0.078893000
N	0.000000000	-1.231838000	0.251175000
C	0.000000000	-1.139069000	1.646559000
C	0.000000000	-2.454835000	2.176601000
C	0.000000000	-3.319632000	1.104976000
C	0.000000000	-2.534337000	-0.078893000
B	0.000000000	0.000000000	-0.685947000
N	0.000000000	0.000000000	2.310980000
F	1.142029000	0.000000000	-1.499257000
F	-1.142029000	0.000000000	-1.499257000
H	0.000000000	4.398816000	1.133708000
H	0.000000000	-4.398816000	1.133708000
C	0.000000000	-2.998940000	-1.498823000
H	-0.871883000	-2.563162000	-1.999646000
H	0.871883000	-2.563162000	-1.999646000
C	0.000000000	-4.515998000	-1.661532000
H	-0.885797000	-4.968896000	-1.207379000
H	0.885797000	-4.968896000	-1.207379000
H	0.000000000	-4.775088000	-2.722711000
C	0.000000000	2.998940000	-1.498823000
H	0.871883000	2.563162000	-1.999646000
H	-0.871883000	2.563162000	-1.999646000
C	0.000000000	4.515998000	-1.661532000
H	0.885797000	4.968896000	-1.207379000
H	-0.885797000	4.968896000	-1.207379000
H	0.000000000	4.775088000	-2.722711000
H	0.000000000	-2.691636000	3.231250000
H	0.000000000	2.691636000	3.231250000

60

C1 GS CAM-B3LYP/6-31+G(d,p)

N	-0.678532000	-1.252136000	0.147489000
C	0.712686000	-1.140964000	0.180105000
C	1.287658000	-2.448959000	0.084973000
C	0.217871000	-3.315737000	-0.045170000
C	-0.978433000	-2.563626000	0.009717000
N	-0.695442000	1.237801000	0.113872000
C	0.697586000	1.143782000	0.096360000
C	1.252018000	2.455232000	-0.049389000
C	0.166983000	3.309374000	-0.128905000
C	-1.016956000	2.544454000	-0.024934000
B	-1.599969000	-0.011261000	0.297623000
N	1.356853000	0.005954000	0.156389000
F	-2.144033000	-0.000847000	1.599431000
F	-2.618534000	-0.029368000	-0.649672000
H	0.258978000	-4.392974000	-0.110774000
H	0.192957000	4.382172000	-0.250320000
C	-2.372391000	3.110145000	-0.080453000
C	-3.422517000	2.655640000	0.727487000
C	-2.602730000	4.188735000	-0.947916000
C	-4.670631000	3.264850000	0.661988000
C	-3.854642000	4.787489000	-1.017005000
C	-4.893536000	4.327697000	-0.210435000
H	-3.259011000	1.836710000	1.415583000
H	-1.802252000	4.545489000	-1.587376000
H	-5.472802000	2.907450000	1.299664000
H	-4.018783000	5.612140000	-1.702991000
H	-5.871006000	4.796744000	-0.260211000

C	-2.324434000	-3.147631000	-0.062397000
C	-2.523534000	-4.245364000	-0.913233000
C	-3.395762000	-2.690766000	0.715961000
C	-3.766566000	-4.860551000	-0.996267000
C	-4.634757000	-3.316468000	0.637091000
C	-4.826891000	-4.398146000	-0.219567000
H	-1.705106000	-4.604204000	-1.528443000
H	-3.255725000	-1.857303000	1.391897000
H	-3.907069000	-5.700143000	-1.669222000
H	-5.454039000	-2.957493000	1.251673000
H	-5.797495000	-4.880019000	-0.280009000
C	2.713202000	-2.789212000	0.103208000
C	3.168802000	-3.915371000	-0.597702000
C	3.639282000	-2.027537000	0.829806000
C	4.512481000	-4.269088000	-0.575320000
C	4.983293000	-2.384585000	0.851001000
C	5.425111000	-3.504224000	0.149430000
H	2.467391000	-4.505912000	-1.178210000
H	3.300695000	-1.160696000	1.384147000
H	4.848887000	-5.140048000	-1.128650000
H	5.687183000	-1.788072000	1.422689000
H	6.474602000	-3.780382000	0.167775000
C	2.671569000	2.823145000	-0.101374000
C	3.638088000	1.968101000	-0.649784000
C	3.078313000	4.074052000	0.385779000
C	4.972293000	2.357315000	-0.706810000
C	4.412436000	4.459124000	0.328602000
C	5.365220000	3.601407000	-0.218369000
H	3.338785000	1.002384000	-1.037559000

H	2.345937000	4.741052000	0.828987000
H	5.707207000	1.686314000	-1.140245000
H	4.709517000	5.428233000	0.716608000
H	6.407264000	3.901579000	-0.264069000

54

C2 GS CAM-B3LYP/6-31+G(d,p)

N	0.297125000	1.299549000	0.157720000
C	-0.998759000	0.782387000	0.194331000
C	-1.932094000	1.867558000	0.100020000
C	-1.162578000	3.010853000	-0.043693000
C	0.198095000	2.641307000	0.007162000
N	1.052428000	-1.069330000	0.151359000
C	-0.303232000	-1.390928000	0.140531000
C	-0.446763000	-2.806112000	-0.010886000
C	0.846383000	-3.303045000	-0.106316000
C	1.745689000	-2.225148000	0.001175000
B	1.546393000	0.390595000	0.319370000
N	-1.275238000	-0.503517000	0.184594000
F	2.069827000	0.559440000	1.619255000
F	2.514446000	0.700447000	-0.630814000
H	-1.518063000	4.028114000	-0.118758000
H	1.140335000	-4.335028000	-0.224508000
C	-1.674079000	-3.571065000	-0.063863000
S	-3.256385000	-2.853581000	-0.182385000
C	-1.770915000	-4.944689000	-0.045048000
C	-4.012717000	-4.397598000	-0.204865000
C	-3.106682000	-5.417270000	-0.123359000
H	-0.908282000	-5.595247000	0.033113000
H	-5.089634000	-4.468403000	-0.275733000

H	-3.379719000	-6.465296000	-0.118101000
C	-3.373899000	1.768116000	0.148332000
S	-4.375156000	3.060329000	-0.453949000
C	-4.149425000	0.756499000	0.663908000
C	-5.812162000	2.226918000	-0.009683000
C	-5.542839000	1.020128000	0.571594000
H	-3.726435000	-0.138551000	1.099007000
H	-6.777605000	2.672526000	-0.206265000
H	-6.310915000	0.343549000	0.925863000
C	1.313482000	3.593425000	-0.081680000
C	2.470744000	3.485473000	0.699966000
C	1.181231000	4.685879000	-0.951915000
C	3.470565000	4.446886000	0.605676000
C	2.188203000	5.638199000	-1.050305000
C	3.336674000	5.522078000	-0.270026000
H	2.581479000	2.659235000	1.390056000
H	0.294090000	4.776924000	-1.569824000
H	4.358510000	4.355805000	1.222981000
H	2.076115000	6.470059000	-1.738024000
H	4.122410000	6.267270000	-0.342452000
C	3.208313000	-2.360387000	-0.054368000
C	4.071881000	-1.621490000	0.764121000
C	3.752477000	-3.311236000	-0.930616000
C	5.445203000	-1.829602000	0.700880000
C	5.126312000	-3.508704000	-0.997036000
C	5.977917000	-2.768520000	-0.179622000
H	3.668623000	-0.895204000	1.457706000
H	3.096248000	-3.883084000	-1.578211000
H	6.102218000	-1.255691000	1.346499000

H	5.531519000	-4.239369000	-1.689506000
H	7.050989000	-2.923916000	-0.227522000

54

C3 GS CAM-B3LYP/6-31+G(d,p)

N	0.000000000	1.247306000	-0.396909000
C	0.007116000	1.140058000	0.993994000
C	-0.073306000	2.452384000	1.556356000
C	-0.186768000	3.322187000	0.483773000
C	-0.136609000	2.562193000	-0.702566000
N	0.000000000	-1.247306000	-0.396909000
C	-0.007116000	-1.140058000	0.993994000
C	0.073306000	-2.452384000	1.556356000
C	0.186768000	-3.322187000	0.483773000
C	0.136609000	-2.562193000	-0.702566000
B	0.000000000	0.000000000	-1.325403000
N	0.000000000	0.000000000	1.650853000
F	1.146708000	-0.046697000	-2.128224000
F	-1.146708000	0.046697000	-2.128224000
H	-0.251689000	4.398633000	0.528511000
H	0.251689000	-4.398633000	0.528511000
C	0.032400000	-2.790768000	2.951874000
O	0.311633000	-4.091742000	3.269742000
C	-0.265309000	-2.098839000	4.093888000
C	0.195185000	-4.214648000	4.613690000
C	-0.156559000	-3.025424000	5.171590000
H	-0.543016000	-1.057777000	4.142649000
H	0.391896000	-5.195180000	5.018604000
H	-0.322155000	-2.833148000	6.221373000
C	-0.032400000	2.790768000	2.951874000

O	-0.311633000	4.091742000	3.269742000
C	0.265309000	2.098839000	4.093888000
C	-0.195185000	4.214648000	4.613690000
C	0.156559000	3.025424000	5.171590000
H	0.543016000	1.057777000	4.142649000
H	-0.391896000	5.195180000	5.018604000
H	0.322155000	2.833148000	6.221373000
C	-0.235273000	3.142402000	-2.049780000
C	0.549612000	2.716435000	-3.128603000
C	-1.124255000	4.211639000	-2.238764000
C	0.439208000	3.343102000	-4.365217000
C	-1.237838000	4.827947000	-3.478733000
C	-0.454612000	4.395754000	-4.547054000
H	1.250831000	1.903318000	-2.997557000
H	-1.744368000	4.546496000	-1.413937000
H	1.059150000	3.007511000	-5.190370000
H	-1.939884000	5.644841000	-3.610574000
H	-0.539142000	4.878480000	-5.515436000
C	0.235273000	-3.142402000	-2.049780000
C	-0.549612000	-2.716435000	-3.128603000
C	1.124255000	-4.211639000	-2.238764000
C	-0.439208000	-3.343102000	-4.365217000
C	1.237838000	-4.827947000	-3.478733000
C	0.454612000	-4.395754000	-4.547054000
H	-1.250831000	-1.903318000	-2.997557000
H	1.744368000	-4.546496000	-1.413937000
H	-1.059150000	-3.007511000	-5.190370000
H	1.939884000	-5.644841000	-3.610574000
H	0.539142000	-4.878480000	-5.515436000

58

C4 GS CAM-B3LYP/6-31+G(d,p)

N	0.083443000	1.246668000	-0.670847000
C	0.077395000	1.141891000	0.721053000
C	0.058739000	2.457192000	1.284682000
C	0.000000000	3.324552000	0.213442000
C	0.022146000	2.563566000	-0.978591000
N	-0.083443000	-1.246668000	-0.670847000
C	-0.077395000	-1.141891000	0.721053000
C	-0.058739000	-2.457192000	1.284682000
C	0.000000000	-3.324552000	0.213442000
C	-0.022146000	-2.563566000	-0.978591000
B	0.000000000	0.000000000	-1.599375000
N	0.000000000	0.000000000	1.370117000
F	1.141437000	-0.124211000	-2.398992000
F	-1.141437000	0.124211000	-2.398992000
H	-0.011568000	4.402258000	0.263517000
H	0.011568000	-4.402258000	0.263517000
C	-0.092435000	-2.838583000	2.703881000
N	0.316315000	-4.089743000	2.977867000
C	-0.552635000	-1.972657000	3.700877000
C	0.287446000	-4.502142000	4.243074000
C	-0.575802000	-2.416900000	5.017274000
C	-0.146291000	-3.706814000	5.301626000
H	-0.885413000	-0.976540000	3.441239000
H	0.627993000	-5.518551000	4.423750000
H	-0.930474000	-1.763260000	5.807534000
H	-0.147756000	-4.095899000	6.313602000
C	0.092435000	2.838583000	2.703881000

N	-0.316315000	4.089743000	2.977867000
C	0.552635000	1.972657000	3.700877000
C	-0.287446000	4.502142000	4.243074000
C	0.575802000	2.416900000	5.017274000
C	0.146291000	3.706814000	5.301626000
H	0.885413000	0.976540000	3.441239000
H	-0.627993000	5.518551000	4.423750000
H	0.930474000	1.763260000	5.807534000
H	0.147756000	4.095899000	6.313602000
C	-0.026874000	3.149843000	-2.324613000
C	0.734490000	2.672034000	-3.398872000
C	-0.839885000	4.277515000	-2.518042000
C	0.674398000	3.305031000	-4.635563000
C	-0.904492000	4.899537000	-3.758440000
C	-0.145659000	4.415332000	-4.822149000
H	1.379225000	1.814109000	-3.263948000
H	-1.440278000	4.654396000	-1.696803000
H	1.275747000	2.928931000	-5.456961000
H	-1.548724000	5.762100000	-3.894235000
H	-0.191393000	4.902893000	-5.790713000
C	0.026874000	-3.149843000	-2.324613000
C	-0.734490000	-2.672034000	-3.398872000
C	0.839885000	-4.277515000	-2.518042000
C	-0.674398000	-3.305031000	-4.635563000
C	0.904492000	-4.899537000	-3.758440000
C	0.145659000	-4.415332000	-4.822149000
H	-1.379225000	-1.814109000	-3.263948000
H	1.440278000	-4.654396000	-1.696803000
H	-1.275747000	-2.928931000	-5.456961000

H	1.548724000	-5.762100000	-3.894235000
H	0.191393000	-4.902893000	-5.790713000

58

C5 GS CAM-B3LYP/6-31+G(d,p)

N	-0.683215000	1.250404000	-0.149982000
C	0.706899000	1.145142000	-0.176445000
C	1.272722000	2.455058000	-0.075538000
C	0.202892000	3.318310000	0.045838000
C	-0.992236000	2.559321000	-0.016119000
N	-0.684774000	-1.243288000	-0.114123000
C	0.706072000	-1.138986000	-0.103512000
C	1.267689000	-2.446971000	0.038240000
C	0.193407000	-3.308662000	0.127993000
C	-0.998820000	-2.550002000	0.029750000
B	-1.599567000	0.001947000	-0.298309000
N	1.358485000	0.002299000	-0.158281000
F	-2.139592000	-0.013926000	-1.598577000
F	-2.612607000	0.014778000	0.651477000
H	0.239489000	4.395587000	0.112918000
H	0.227174000	-4.381120000	0.249955000
C	2.690874000	-2.803112000	0.080234000
C	3.669131000	-1.939056000	0.582474000
C	3.115473000	-4.056977000	-0.372321000
C	4.992598000	-2.362227000	0.608239000
C	4.463223000	-4.383231000	-0.303116000
N	5.400240000	-3.559769000	0.177465000
H	2.411582000	-4.767932000	-0.790126000
H	5.763125000	-1.702963000	0.999118000
H	4.805978000	-5.352966000	-0.654022000

H	3.406451000	-0.956437000	0.951658000
C	2.698179000	2.799231000	-0.082526000
C	3.645446000	2.040905000	-0.777373000
C	3.157648000	3.930053000	0.600559000
C	4.976082000	2.440919000	-0.755563000
C	4.509432000	4.244344000	0.559206000
N	5.417347000	3.521060000	-0.104005000
H	3.353682000	1.158199000	-1.331689000
H	2.477944000	4.551446000	1.172758000
H	5.723509000	1.863384000	-1.293024000
H	4.880485000	5.118444000	1.087590000
C	-2.339925000	3.137272000	0.049505000
C	-3.408909000	2.667854000	-0.724910000
C	-2.543961000	4.241536000	0.891120000
C	-4.650813000	3.287881000	-0.650928000
C	-3.790164000	4.850268000	0.969590000
C	-4.848066000	4.375478000	0.197003000
H	-3.264894000	1.830664000	-1.395212000
H	-1.727910000	4.610243000	1.503579000
H	-5.468242000	2.919981000	-1.262559000
H	-3.935086000	5.694621000	1.635488000
H	-5.821093000	4.852818000	0.253618000
C	-2.348560000	-3.125170000	0.091209000
C	-3.407736000	-2.672743000	-0.706356000
C	-2.564222000	-4.210179000	0.954801000
C	-4.651032000	-3.290367000	-0.633894000
C	-3.811794000	-4.816391000	1.031341000
C	-4.859804000	-4.358671000	0.235398000
H	-3.255032000	-1.850623000	-1.393031000

H	-1.756731000	-4.565562000	1.586104000
H	-5.460487000	-2.935529000	-1.263599000
H	-3.965427000	-5.645408000	1.714355000
H	-5.833814000	-4.834159000	0.290645000

58

C6 GS CAM-B3LYP/6-31+G(d,p)

N	-0.663419000	1.253599000	-0.139885000
C	0.727290000	1.152338000	-0.172305000
C	1.287416000	2.464747000	-0.061379000
C	0.215944000	3.321775000	0.081815000
C	-0.975436000	2.560845000	0.014352000
N	-0.661916000	-1.238352000	-0.133492000
C	0.730370000	-1.136958000	-0.116564000
C	1.286275000	-2.449991000	0.023420000
C	0.208254000	-3.307666000	0.101053000
C	-0.979512000	-2.546264000	0.000423000
B	-1.575596000	0.007144000	-0.304980000
N	1.378255000	0.008018000	-0.161586000
F	-2.115606000	0.007776000	-1.607201000
F	-2.593109000	0.006055000	0.641943000
H	0.265080000	4.395176000	0.179750000
H	0.254847000	-4.380790000	0.199676000
C	2.700969000	-2.849670000	0.086206000
N	2.933163000	-4.165840000	-0.063157000
C	3.735502000	-1.936054000	0.310694000
C	4.189707000	-4.599399000	-0.001766000
C	5.042708000	-2.403637000	0.372138000
C	5.282703000	-3.762047000	0.211746000
H	3.509992000	-0.885949000	0.437159000

H	4.334379000	-5.669190000	-0.129776000
H	5.860791000	-1.712581000	0.547604000
H	6.286012000	-4.170920000	0.252580000
C	2.706223000	2.846078000	-0.084485000
N	3.000463000	4.033235000	0.473267000
C	3.682454000	2.043273000	-0.682634000
C	4.267921000	4.440235000	0.461150000
C	5.001405000	2.480379000	-0.685917000
C	5.307574000	3.702429000	-0.100940000
H	3.404774000	1.102560000	-1.139527000
H	4.466297000	5.403664000	0.923870000
H	5.776074000	1.875407000	-1.145713000
H	6.322191000	4.084041000	-0.081029000
C	-2.325287000	3.132860000	0.093987000
C	-3.395796000	2.672068000	-0.683482000
C	-2.529768000	4.224248000	0.952048000
C	-4.639258000	3.287470000	-0.596444000
C	-3.777382000	4.828465000	1.043477000
C	-4.836796000	4.362148000	0.267771000
H	-3.251731000	1.844733000	-1.365977000
H	-1.712000000	4.586589000	1.565986000
H	-5.457763000	2.926045000	-1.210576000
H	-3.922122000	5.662883000	1.721893000
H	-5.810943000	4.835945000	0.334554000
C	-2.331042000	-3.119517000	0.046372000
C	-3.385332000	-2.657848000	-0.752355000
C	-2.553025000	-4.214362000	0.895866000
C	-4.629441000	-3.275576000	-0.694719000
C	-3.801251000	-4.821036000	0.957724000

C	-4.844313000	-4.353747000	0.160807000
H	-3.228155000	-1.827715000	-1.428233000
H	-1.748703000	-4.577778000	1.526575000
H	-5.434795000	-2.912969000	-1.325343000
H	-3.959212000	-5.658141000	1.629868000
H	-5.818889000	-4.829356000	0.204489000

40

C7 GS CAM-B3LYP/6-31+G(d,p)

N	1.173558000	0.107117000	1.247060000
C	2.567742000	0.099044000	1.141476000
C	3.121818000	-0.027242000	2.440756000
C	2.067432000	-0.118845000	3.313911000
C	0.865431000	-0.025108000	2.555590000
N	1.173558000	0.107117000	-1.247060000
C	2.567742000	0.099044000	-1.141476000
C	3.121818000	-0.027242000	-2.440756000
C	2.067432000	-0.118845000	-3.313911000
C	0.865431000	-0.025108000	-2.555590000
B	0.259749000	0.281585000	0.000000000
N	3.225999000	0.109295000	0.000000000
F	-0.269677000	1.587362000	0.000000000
F	-0.763604000	-0.658543000	0.000000000
H	2.109225000	-0.214170000	4.388744000
H	2.109225000	-0.214170000	-4.388744000
C	-0.481897000	-0.071259000	-3.137046000
C	-1.542164000	0.717871000	-2.671942000
C	-0.695424000	-0.911012000	-4.241236000
C	-2.783591000	0.659824000	-3.294883000
C	-1.940935000	-0.973015000	-4.853440000

C	-2.989838000	-0.185989000	-4.382393000
H	-1.391411000	1.387050000	-1.835158000
H	0.112860000	-1.534926000	-4.607662000
H	-3.593545000	1.282770000	-2.929296000
H	-2.092350000	-1.637613000	-5.697738000
H	-3.962269000	-0.229830000	-4.862325000
C	-0.481897000	-0.071259000	3.137046000
C	-0.695424000	-0.911012000	4.241236000
C	-1.542164000	0.717871000	2.671942000
C	-1.940935000	-0.973015000	4.853440000
C	-2.783591000	0.659824000	3.294883000
C	-2.989838000	-0.185989000	4.382393000
H	0.112860000	-1.534926000	4.607662000
H	-1.391411000	1.387050000	1.835158000
H	-2.092350000	-1.637613000	5.697738000
H	-3.593545000	1.282770000	2.929296000
H	-3.962269000	-0.229830000	4.862325000
H	4.180532000	-0.054906000	2.655816000
H	4.180532000	-0.054906000	-2.655816000

40

D1 GS CAM-B3LYP/6-31+G(d,p)

N	0.014242000	1.224168000	2.316483000
C	-0.013086000	1.140936000	0.923446000
C	0.000000000	2.472393000	0.388294000
C	0.051251000	3.321057000	1.489347000
C	0.053867000	2.520050000	2.641226000
N	-0.014242000	-1.224168000	2.316483000
C	0.013086000	-1.140936000	0.923446000
C	0.000000000	-2.472393000	0.388294000

C	-0.051251000	-3.321057000	1.489347000
C	-0.053867000	-2.520050000	2.641226000
B	0.000000000	0.000000000	3.260678000
N	0.000000000	0.000000000	0.264740000
F	-1.140011000	0.010863000	4.071893000
F	1.140011000	-0.010863000	4.071893000
C	-0.023086000	2.856006000	-1.025655000
H	0.055504000	4.400989000	1.483005000
H	-0.055504000	-4.400989000	1.483005000
C	0.023086000	-2.856006000	-1.025655000
C	-0.689437000	2.466020000	-3.323721000
C	-0.665231000	2.067584000	-1.991569000
C	0.564817000	4.445746000	-2.762431000
C	0.689437000	-2.466020000	-3.323721000
C	-0.075311000	3.653908000	-3.714512000
C	0.589865000	4.051302000	-1.430367000
C	0.665231000	-2.067584000	-1.991569000
C	0.075311000	-3.653908000	-3.714512000
H	-0.095933000	3.962042000	-4.754992000
H	1.152729000	-1.147569000	-1.693383000
H	0.095933000	-3.962042000	-4.754992000
H	1.050082000	5.370079000	-3.058987000
H	1.195718000	-1.847941000	-4.058354000
H	1.105549000	4.664667000	-0.698677000
C	-0.589865000	-4.051302000	-1.430367000
C	-0.564817000	-4.445746000	-2.762431000
H	-1.195718000	1.847941000	-4.058354000
H	-1.152729000	1.147569000	-1.693383000
H	-1.105549000	-4.664667000	-0.698677000

H	-1.050082000	-5.370079000	-3.058987000
H	0.073607000	2.834484000	3.676340000
H	-0.073607000	-2.834484000	3.676340000

34

D2 GS CAM-B3LYP/6-31+G(d,p)

N	0.868530000	2.352886000	0.005119000
C	0.915339000	0.958852000	-0.032612000
C	2.295963000	0.555046000	-0.038733000
C	3.036580000	1.735400000	0.015213000
C	2.129216000	2.800161000	0.037478000
N	-1.566022000	2.131321000	-0.020212000
C	-1.353614000	0.754896000	-0.013823000
C	-2.628138000	0.097685000	-0.008160000
C	-3.581188000	1.118964000	-0.021169000
C	-2.891169000	2.334048000	-0.027359000
B	-0.435918000	3.181903000	-0.004607000
N	-0.158833000	0.197502000	-0.029354000
F	-0.497154000	3.996402000	-1.141960000
F	-0.517996000	3.987963000	1.137060000
H	4.112293000	1.833359000	0.019032000
H	-4.655424000	1.012524000	-0.010259000
C	-2.885083000	-1.324510000	0.005977000
S	-1.659646000	-2.521551000	0.319475000
C	-4.105618000	-1.930355000	-0.197869000
C	-2.797104000	-3.801692000	0.173281000
C	-4.055045000	-3.344827000	-0.104838000
H	-5.009406000	-1.375545000	-0.418606000
H	-2.473805000	-4.826176000	0.298289000
H	-4.912785000	-3.991966000	-0.240055000

C	2.798721000	-0.797966000	-0.094281000
S	4.475936000	-1.134303000	0.240471000
C	2.130031000	-1.956821000	-0.417465000
C	4.254418000	-2.812848000	-0.053684000
C	2.963253000	-3.106419000	-0.392463000
H	1.078365000	-1.972495000	-0.668260000
H	5.089628000	-3.492798000	0.043218000
H	2.618803000	-4.107726000	-0.620074000
H	2.342647000	3.860330000	0.068427000
H	-3.300120000	3.335700000	-0.029531000

34

D3 GS CAM-B3LYP/6-31+G(d,p)

N	-0.020866000	1.222386000	-1.993564000
C	0.002232000	1.138036000	-0.601946000
C	0.000000000	2.472734000	-0.076619000
C	-0.042699000	3.326309000	-1.178182000
C	-0.050633000	2.521010000	-2.322089000
N	0.020866000	-1.222386000	-1.993564000
C	-0.002232000	-1.138036000	-0.601946000
C	0.000000000	-2.472734000	-0.076619000
C	0.042699000	-3.326309000	-1.178182000
C	0.050633000	-2.521010000	-2.322089000
B	0.000000000	0.000000000	-2.937654000
N	0.000000000	0.000000000	0.063055000
F	1.139471000	0.018598000	-3.750674000
F	-1.139471000	-0.018598000	-3.750674000
H	-0.055673000	4.405354000	-1.163173000
H	0.055673000	-4.405354000	-1.163173000
C	-0.044658000	-2.850339000	1.306729000

O	0.097414000	-4.184002000	1.576962000
C	-0.228638000	-2.168228000	2.479153000
C	0.007940000	-4.337436000	2.918918000
C	-0.192082000	-3.135494000	3.523953000
H	-0.384716000	-1.105098000	2.568819000
H	0.105597000	-5.346535000	3.288144000
H	-0.303553000	-2.961111000	4.583894000
C	0.044658000	2.850339000	1.306729000
O	-0.097414000	4.184002000	1.576962000
C	0.228638000	2.168228000	2.479153000
C	-0.007940000	4.337436000	2.918918000
C	0.192082000	3.135494000	3.523953000
H	0.384716000	1.105098000	2.568819000
H	-0.105597000	5.346535000	3.288144000
H	0.303553000	2.961111000	4.583894000
H	-0.072371000	2.830804000	-3.358429000
H	0.072371000	-2.830804000	-3.358429000

38

D4 GS CAM-B3LYP/6-31+G(d,p)

N	0.013569000	1.225445000	2.306121000
C	-0.010618000	1.140093000	0.914190000
C	0.000000000	2.470029000	0.378373000
C	0.043824000	3.321908000	1.475131000
C	0.047240000	2.520982000	2.629373000
N	-0.013569000	-1.225445000	2.306121000
C	0.010618000	-1.140093000	0.914190000
C	0.000000000	-2.470029000	0.378373000
C	-0.043824000	-3.321908000	1.475131000
C	-0.047240000	-2.520982000	2.629373000

B	0.000000000	0.000000000	3.252617000
N	0.000000000	0.000000000	0.254477000
F	-1.140356000	0.010601000	4.059868000
F	1.140356000	-0.010601000	4.059868000
H	0.043021000	4.401850000	1.468254000
H	-0.043021000	-4.401850000	1.468254000
C	0.015415000	-2.839062000	-1.037491000
C	-0.605288000	-4.023446000	-1.457596000
C	0.641675000	-2.060137000	-2.016831000
N	-0.625278000	-4.446547000	-2.719981000
C	0.621530000	-2.491865000	-3.335372000
C	-0.017719000	-3.689206000	-3.639444000
H	1.141978000	-1.138959000	-1.743756000
H	1.101997000	-1.915790000	-4.118235000
H	-0.042736000	-4.057874000	-4.661252000
H	-1.124542000	-4.647415000	-0.734829000
C	-0.015415000	2.839062000	-1.037491000
C	0.605288000	4.023446000	-1.457596000
C	-0.641675000	2.060137000	-2.016831000
N	0.625278000	4.446547000	-2.719981000
C	-0.621530000	2.491865000	-3.335372000
C	0.017719000	3.689206000	-3.639444000
H	1.124542000	4.647415000	-0.734829000
H	-1.141978000	1.138959000	-1.743756000
H	-1.101997000	1.915790000	-4.118235000
H	0.042736000	4.057874000	-4.661252000
H	0.063910000	2.837545000	3.663817000
H	-0.063910000	-2.837545000	3.663817000

38

D5 GS CAM-B3LYP/6-31+G(d,p)

N	0.009663000	1.226276000	2.311716000
C	-0.011728000	1.140481000	0.919996000
C	0.000000000	2.471013000	0.385996000
C	0.039068000	3.322080000	1.481115000
C	0.039237000	2.520525000	2.636543000
N	-0.009663000	-1.226276000	2.311716000
C	0.011728000	-1.140481000	0.919996000
C	0.000000000	-2.471013000	0.385996000
C	-0.039068000	-3.322080000	1.481115000
C	-0.039237000	-2.520525000	2.636543000
B	0.000000000	0.000000000	3.259317000
N	0.000000000	0.000000000	0.261362000
F	-1.140635000	0.007543000	4.063882000
F	1.140635000	-0.007543000	4.063882000
H	0.039306000	4.402029000	1.473432000
H	-0.039306000	-4.402029000	1.473432000
C	0.016425000	-2.849591000	-1.030492000
C	-0.583655000	-4.041289000	-1.450840000
C	0.637118000	-2.064356000	-2.007171000
C	-0.537530000	-4.385800000	-2.795075000
C	0.627214000	-2.499169000	-3.327015000
N	0.054775000	-3.637072000	-3.730871000
H	1.126414000	-1.134787000	-1.746585000
H	-1.002197000	-5.307029000	-3.136029000
H	1.107898000	-1.901587000	-4.096955000
H	-1.096522000	-4.687300000	-0.747244000
C	-0.016425000	2.849591000	-1.030492000
C	0.583655000	4.041289000	-1.450840000

C	-0.637118000	2.064356000	-2.007171000
C	0.537530000	4.385800000	-2.795075000
C	-0.627214000	2.499169000	-3.327015000
N	-0.054775000	3.637072000	-3.730871000
H	1.096522000	4.687300000	-0.747244000
H	-1.126414000	1.134787000	-1.746585000
H	1.002197000	5.307029000	-3.136029000
H	-1.107898000	1.901587000	-4.096955000
H	0.053125000	2.836643000	3.671124000
H	-0.053125000	-2.836643000	3.671124000

38

D6 GS CAM-B3LYP/6-31+G(d,p)

N	0.000000000	1.225209000	2.282275000
C	-0.025351000	1.142472000	0.889717000
C	-0.032507000	2.477039000	0.361524000
C	0.007133000	3.324130000	1.459438000
C	0.020372000	2.520016000	2.610706000
N	0.000000000	-1.225209000	2.282275000
C	0.025351000	-1.142472000	0.889717000
C	0.032507000	-2.477039000	0.361524000
C	-0.007133000	-3.324130000	1.459438000
C	-0.020372000	-2.520016000	2.610706000
B	0.000000000	0.000000000	3.227362000
N	0.000000000	0.000000000	0.233599000
F	-1.140360000	-0.001547000	4.035366000
F	1.140360000	0.001547000	4.035366000
H	0.014083000	4.402715000	1.438789000
H	-0.014083000	-4.402715000	1.438789000
C	0.072648000	-2.896096000	-1.046314000

N	-0.288939000	-4.169528000	-1.281957000
C	0.489201000	-2.040467000	-2.070995000
C	-0.255988000	-4.614872000	-2.535414000
C	0.517356000	-2.519408000	-3.375185000
C	0.136263000	-3.832251000	-3.619746000
H	0.787837000	-1.026147000	-1.842141000
H	-0.557912000	-5.648334000	-2.685089000
H	0.838785000	-1.874491000	-4.186480000
H	0.143616000	-4.248692000	-4.620721000
C	-0.072648000	2.896096000	-1.046314000
N	0.288939000	4.169528000	-1.281957000
C	-0.489201000	2.040467000	-2.070995000
C	0.255988000	4.614872000	-2.535414000
C	-0.517356000	2.519408000	-3.375185000
C	-0.136263000	3.832251000	-3.619746000
H	-0.787837000	1.026147000	-1.842141000
H	0.557912000	5.648334000	-2.685089000
H	-0.838785000	1.874491000	-4.186480000
H	-0.143616000	4.248692000	-4.620721000
H	0.039935000	2.831311000	3.646742000
H	-0.039935000	-2.831311000	3.646742000

20

D7 GS CAM-B3LYP/6-31+G(d,p)

N	0.000000000	1.226503000	-0.297754000
C	0.000000000	1.139653000	1.098027000
C	0.000000000	2.460102000	1.623440000
C	0.000000000	3.321902000	0.547038000
C	0.000000000	2.521013000	-0.618177000
N	0.000000000	-1.226503000	-0.297754000

C	0.000000000	-1.139653000	1.098027000
C	0.000000000	-2.460102000	1.623440000
C	0.000000000	-3.321902000	0.547038000
C	0.000000000	-2.521013000	-0.618177000
B	0.000000000	0.000000000	-1.245289000
N	0.000000000	0.000000000	1.761804000
F	1.140879000	0.000000000	-2.050907000
F	-1.140879000	0.000000000	-2.050907000
H	0.000000000	2.700392000	2.677147000
H	0.000000000	4.401704000	0.562460000
H	0.000000000	2.839776000	-1.652236000
H	0.000000000	-2.700392000	2.677147000
H	0.000000000	-4.401704000	0.562460000
H	0.000000000	-2.839776000	-1.652236000

54

A1 GS CAM-B3LYP/6-31G(d)

N	0.059246000	1.242983000	-0.736344000
C	0.049123000	1.141344000	0.652867000
C	0.027667000	2.455674000	1.221362000
C	0.000000000	3.322322000	0.146534000
C	0.026689000	2.561245000	-1.046127000
N	-0.059246000	-1.242983000	-0.736344000
C	-0.049123000	-1.141344000	0.652867000
C	-0.027667000	-2.455674000	1.221362000
C	0.000000000	-3.322322000	0.146534000
C	-0.026689000	-2.561245000	-1.046127000
B	0.000000000	0.000000000	-1.671713000
N	0.000000000	0.000000000	1.305309000
F	1.139106000	-0.086901000	-2.464366000

F	-1.139106000	0.086901000	-2.464366000
C	0.021249000	2.807254000	2.644338000
H	0.019273000	4.401907000	0.184897000
C	0.023189000	3.118813000	-2.380890000
C	-0.023189000	-3.118813000	-2.380890000
H	-0.019273000	-4.401907000	0.184897000
C	-0.021249000	-2.807254000	2.644338000
C	0.577061000	2.338061000	4.954759000
C	0.581858000	1.967116000	3.615990000
C	-0.541483000	4.393110000	4.390930000
C	-0.577061000	-2.338061000	4.954759000
C	0.017496000	3.550182000	5.347764000
C	-0.539520000	4.025217000	3.052653000
C	-0.581858000	-1.967116000	3.615990000
C	-0.017496000	-3.550182000	5.347764000
H	0.016137000	3.836980000	6.394609000
H	-1.021500000	-1.024287000	3.316403000
H	-0.016137000	-3.836980000	6.394609000
H	-0.985333000	5.337855000	4.688344000
H	-1.018468000	-1.677445000	5.694375000
H	-0.995106000	4.678905000	2.315900000
S	0.555250000	-4.750566000	-2.611804000
C	0.220869000	-4.686948000	-4.294524000
C	-0.306905000	-3.484299000	-4.664376000
C	-0.447876000	-2.586760000	-3.575196000
C	0.447876000	2.586760000	-3.575196000
C	0.306905000	3.484299000	-4.664376000
C	-0.220869000	4.686948000	-4.294524000
S	-0.555250000	4.750566000	-2.611804000

H	0.439075000	-5.540735000	-4.921096000
H	-0.591892000	-3.244294000	-5.681378000
H	-0.869615000	-1.595725000	-3.655294000
H	0.869615000	1.595725000	-3.655294000
H	0.591892000	3.244294000	-5.681378000
H	-0.439075000	5.540735000	-4.921096000
C	0.539520000	-4.025217000	3.052653000
C	0.541483000	-4.393110000	4.390930000
H	1.018468000	1.677445000	5.694375000
H	1.021500000	1.024287000	3.316403000
H	0.995106000	-4.678905000	2.315900000
H	0.985333000	-5.337855000	4.688344000

48

A2 GS CAM-B3LYP/6-31G(d)

N	0.771651000	-1.113393000	0.114461000
C	1.221524000	0.204740000	0.118930000
C	2.655806000	0.205756000	0.147810000
C	3.031507000	-1.124767000	0.122335000
C	1.862814000	-1.917784000	0.108367000
N	-1.510979000	-0.147454000	-0.058916000
C	-0.874559000	1.088749000	-0.040515000
C	-1.860267000	2.125084000	-0.077975000
C	-3.083771000	1.475653000	-0.092609000
C	-2.848236000	0.085109000	-0.082423000
B	-0.735974000	-1.491957000	0.025998000
N	0.429229000	1.252192000	0.042673000
F	-1.146912000	-2.183058000	1.160633000
F	-0.939971000	-2.263525000	-1.112822000
H	4.039070000	-1.513310000	0.165487000

H	-4.059950000	1.933957000	-0.153107000
C	-1.637953000	3.553772000	-0.101631000
S	-0.079838000	4.281421000	-0.380860000
C	-2.597776000	4.525543000	0.054209000
C	-0.745302000	5.865111000	-0.288286000
C	-2.088145000	5.845317000	-0.050140000
H	-3.639406000	4.298241000	0.247012000
H	-0.103753000	6.727261000	-0.408775000
H	-2.694959000	6.736906000	0.049415000
C	3.517042000	1.365839000	0.200177000
S	5.207242000	1.231856000	-0.206219000
C	3.213613000	2.650719000	0.576712000
C	5.475598000	2.897294000	0.132783000
C	4.333661000	3.523882000	0.536131000
H	2.219256000	2.949296000	0.878305000
H	6.462330000	3.321982000	0.010673000
H	4.289332000	4.573469000	0.800239000
C	1.849376000	-3.364261000	0.093346000
S	3.267162000	-4.214645000	-0.469316000
C	0.884007000	-4.255143000	0.498257000
C	2.541733000	-5.738474000	-0.155074000
C	1.282790000	-5.608646000	0.354885000
H	-0.064573000	-3.941153000	0.908099000
H	3.083072000	-6.649076000	-0.371610000
H	0.657888000	-6.450929000	0.625295000
C	-3.882863000	-0.927141000	-0.092421000
S	-5.490184000	-0.494461000	0.436218000
C	-3.847771000	-2.240259000	-0.496436000
C	-6.080019000	-2.073356000	0.108175000

C	-5.103226000	-2.890728000	-0.380525000
H	-2.955604000	-2.707104000	-0.886792000
H	-7.116381000	-2.313782000	0.301193000
H	-5.271412000	-3.924923000	-0.654900000

48

A3 GS CAM-B3LYP/6-31G(d)

N	0.045424000	1.242195000	-0.446812000
C	0.048631000	1.137514000	0.940916000
C	0.044513000	2.453065000	1.501506000
C	0.000000000	3.327143000	0.430955000
C	0.008708000	2.563609000	-0.756650000
N	-0.045424000	-1.242195000	-0.446812000
C	-0.048631000	-1.137514000	0.940916000
C	-0.044513000	-2.453065000	1.501506000
C	0.000000000	-3.327143000	0.430955000
C	-0.008708000	-2.563609000	-0.756650000
B	0.000000000	0.000000000	-1.383040000
N	0.000000000	0.000000000	1.601260000
F	1.139869000	-0.071538000	-2.176458000
F	-1.139869000	0.071538000	-2.176458000
H	0.005722000	4.405718000	0.481329000
H	-0.005722000	-4.405718000	0.481329000
C	-0.094327000	-2.788842000	2.896479000
O	0.099772000	-4.104662000	3.210327000
C	-0.334481000	-2.078523000	4.039658000
C	-0.012861000	-4.218036000	4.553338000
C	-0.278621000	-3.010101000	5.115382000
H	-0.543754000	-1.021663000	4.089704000
H	0.121906000	-5.210075000	4.955539000

H	-0.421038000	-2.806813000	6.166421000
C	0.094327000	2.788842000	2.896479000
O	-0.099772000	4.104662000	3.210327000
C	0.334481000	2.078523000	4.039658000
C	0.012861000	4.218036000	4.553338000
C	0.278621000	3.010101000	5.115382000
H	0.543754000	1.021663000	4.089704000
H	-0.121906000	5.210075000	4.955539000
H	0.421038000	2.806813000	6.166421000
C	-0.018857000	3.117864000	-2.093099000
C	0.396638000	2.588199000	-3.291401000
C	0.232709000	3.482487000	-4.380308000
C	-0.303039000	4.680071000	-4.005910000
S	-0.617182000	4.742949000	-2.319074000
H	0.828524000	1.601866000	-3.375145000
H	0.507460000	3.243606000	-5.400396000
H	-0.538325000	5.530341000	-4.631075000
C	0.018857000	-3.117864000	-2.093099000
C	-0.396638000	-2.588199000	-3.291401000
C	-0.232709000	-3.482487000	-4.380308000
C	0.303039000	-4.680071000	-4.005910000
S	0.617182000	-4.742949000	-2.319074000
H	-0.828524000	-1.601866000	-3.375145000
H	-0.507460000	-3.243606000	-5.400396000
H	0.538325000	-5.530341000	-4.631075000

52

A4 GS CAM-B3LYP/6-31G(d)

N	0.058331000	1.243777000	-0.729540000
C	0.046840000	1.140286000	0.658369000

C	0.025057000	2.453147000	1.226651000
C	0.000000000	3.323107000	0.156389000
C	0.027052000	2.562059000	-1.038682000
N	-0.058331000	-1.243777000	-0.729540000
C	-0.046840000	-1.140286000	0.658369000
C	-0.025057000	-2.453147000	1.226651000
C	0.000000000	-3.323107000	0.156389000
C	-0.027052000	-2.562059000	-1.038682000
B	0.000000000	0.000000000	-1.666484000
N	0.000000000	0.000000000	1.313053000
F	1.139216000	-0.086305000	-2.456824000
F	-1.139216000	0.086305000	-2.456824000
H	0.020357000	4.402620000	0.196449000
H	-0.020357000	-4.402620000	0.196449000
C	-0.014075000	-2.790119000	2.650611000
C	0.574550000	-3.985889000	3.080651000
C	-0.583114000	-1.969783000	3.630136000
N	0.615638000	-4.386750000	4.348022000
C	-0.540355000	-2.375484000	4.955397000
C	0.062288000	-3.588943000	5.265012000
H	-1.051003000	-1.034025000	3.350724000
H	-0.975365000	-1.765375000	5.739108000
H	0.102798000	-3.937159000	6.294263000
H	1.051467000	-4.641677000	2.355714000
C	0.014075000	2.790119000	2.650611000
C	-0.574550000	3.985889000	3.080651000
C	0.583114000	1.969783000	3.630136000
N	-0.615638000	4.386750000	4.348022000
C	0.540355000	2.375484000	4.955397000

C	-0.062288000	3.588943000	5.265012000
H	-1.051467000	4.641677000	2.355714000
H	1.051003000	1.034025000	3.350724000
H	0.975365000	1.765375000	5.739108000
H	-0.102798000	3.937159000	6.294263000
C	-0.025469000	-3.121290000	-2.371589000
C	-0.445560000	-2.587477000	-3.567336000
C	-0.308368000	-3.486588000	-4.654958000
C	0.211925000	-4.692115000	-4.282718000
S	0.543254000	-4.756815000	-2.600069000
H	-0.862434000	-1.594572000	-3.649334000
H	-0.590490000	-3.246201000	-5.672608000
H	0.426160000	-5.547683000	-4.908248000
C	0.025469000	3.121290000	-2.371589000
C	0.445560000	2.587477000	-3.567336000
C	0.308368000	3.486588000	-4.654958000
C	-0.211925000	4.692115000	-4.282718000
S	-0.543254000	4.756815000	-2.600069000
H	0.862434000	1.594572000	-3.649334000
H	0.590490000	3.246201000	-5.672608000
H	-0.426160000	5.547683000	-4.908248000

52

A5 GS CAM-B3LYP/6-31G(d)

N	0.060727000	1.244276000	-0.732881000
C	0.046666000	1.140640000	0.654603000
C	0.022178000	2.454289000	1.221071000
C	0.000000000	3.323204000	0.151855000
C	0.031796000	2.561727000	-1.044088000
N	-0.060727000	-1.244276000	-0.732881000

C	-0.046666000	-1.140640000	0.654603000
C	-0.022178000	-2.454289000	1.221071000
C	0.000000000	-3.323204000	0.151855000
C	-0.031796000	-2.561727000	-1.044088000
B	0.000000000	0.000000000	-1.670254000
N	0.000000000	0.000000000	1.308334000
F	1.139181000	-0.088678000	-2.459017000
F	-1.139181000	0.088678000	-2.459017000
H	0.017150000	4.402761000	0.192475000
H	-0.017150000	-4.402761000	0.192475000
C	-0.011369000	-2.801804000	2.645508000
C	0.541878000	-4.013061000	3.071600000
C	-0.558492000	-1.969456000	3.626399000
C	0.522551000	-4.328993000	4.422193000
C	-0.523450000	-2.380324000	4.952235000
N	0.002412000	-3.536517000	5.362475000
H	-1.003587000	-1.019422000	3.362200000
H	0.951580000	-5.267498000	4.765166000
H	-0.946017000	-1.742822000	5.725345000
H	0.998699000	-4.696380000	2.364547000
C	0.011369000	2.801804000	2.645508000
C	-0.541878000	4.013061000	3.071600000
C	0.558492000	1.969456000	3.626399000
C	-0.522551000	4.328993000	4.422193000
C	0.523450000	2.380324000	4.952235000
N	-0.002412000	3.536517000	5.362475000
H	-0.998699000	4.696380000	2.364547000
H	1.003587000	1.019422000	3.362200000
H	-0.951580000	5.267498000	4.765166000

H	0.946017000	1.742822000	5.725345000
C	-0.034286000	-3.120847000	-2.376294000
C	-0.452084000	-2.584570000	-3.572129000
C	-0.320146000	-3.484160000	-4.659526000
C	0.193876000	-4.692589000	-4.287282000
S	0.525226000	-4.759620000	-2.605119000
H	-0.864184000	-1.589713000	-3.654194000
H	-0.601381000	-3.242392000	-5.677057000
H	0.403294000	-5.549225000	-4.912995000
C	0.034286000	3.120847000	-2.376294000
C	0.452084000	2.584570000	-3.572129000
C	0.320146000	3.484160000	-4.659526000
C	-0.193876000	4.692589000	-4.287282000
S	-0.525226000	4.759620000	-2.605119000
H	0.864184000	1.589713000	-3.654194000
H	0.601381000	3.242392000	-5.677057000
H	-0.403294000	5.549225000	-4.912995000

52

A6 GS CAM-B3LYP/6-31G(d)

N	0.057608000	1.243110000	-0.708864000
C	0.053116000	1.142460000	0.680075000
C	0.038777000	2.460341000	1.239515000
C	0.000000000	3.324884000	0.168467000
C	0.023025000	2.560766000	-1.023352000
N	-0.057608000	-1.243110000	-0.708864000
C	-0.053116000	-1.142460000	0.680075000
C	-0.038777000	-2.460341000	1.239515000
C	0.000000000	-3.324884000	0.168467000
C	-0.023025000	-2.560766000	-1.023352000

B	0.000000000	0.000000000	-1.644087000
N	0.000000000	0.000000000	1.332101000
F	1.139340000	-0.083794000	-2.435573000
F	-1.139340000	0.083794000	-2.435573000
H	-0.004870000	4.402663000	0.225714000
H	0.004870000	-4.402663000	0.225714000
C	-0.061309000	-2.857974000	2.653723000
N	0.276917000	-4.135946000	2.893863000
C	-0.438972000	-1.983329000	3.675575000
C	0.259634000	-4.565470000	4.151442000
C	-0.448486000	-2.447394000	4.984455000
C	-0.090368000	-3.764532000	5.235234000
H	-0.720943000	-0.966238000	3.439052000
H	0.540468000	-5.604722000	4.306439000
H	-0.738205000	-1.786892000	5.795290000
H	-0.084601000	-4.169764000	6.240803000
C	0.061309000	2.857974000	2.653723000
N	-0.276917000	4.135946000	2.893863000
C	0.438972000	1.983329000	3.675575000
C	-0.259634000	4.565470000	4.151442000
C	0.448486000	2.447394000	4.984455000
C	0.090368000	3.764532000	5.235234000
H	0.720943000	0.966238000	3.439052000
H	-0.540468000	5.604722000	4.306439000
H	0.738205000	1.786892000	5.795290000
H	0.084601000	4.169764000	6.240803000
C	0.011527000	3.115486000	-2.358007000
C	0.420055000	2.578718000	-3.556539000
C	0.275590000	3.476179000	-4.644539000

C	-0.238498000	4.683695000	-4.269928000
S	-0.554117000	4.752560000	-2.584454000
H	0.834608000	1.585044000	-3.640321000
H	0.548265000	3.233211000	-5.664172000
H	-0.455905000	5.538621000	-4.895252000
C	-0.011527000	-3.115486000	-2.358007000
C	-0.420055000	-2.578718000	-3.556539000
C	-0.275590000	-3.476179000	-4.644539000
C	0.238498000	-4.683695000	-4.269928000
S	0.554117000	-4.752560000	-2.584454000
H	-0.834608000	-1.585044000	-3.640321000
H	-0.548265000	-3.233211000	-5.664172000
H	0.455905000	-5.538621000	-4.895252000

34

A7 GS CAM-B3LYP/6-31G(d)

N	0.038687000	1.244555000	1.060835000
C	0.010675000	1.138850000	2.452316000
C	-0.036614000	2.442437000	3.008235000
C	-0.052441000	3.322419000	1.959435000
C	-0.001694000	2.561617000	0.754927000
N	-0.038687000	-1.244555000	1.060835000
C	-0.010675000	-1.138850000	2.452316000
C	0.036614000	-2.442437000	3.008235000
C	0.052441000	-3.322419000	1.959435000
C	0.001694000	-2.561617000	0.754927000
B	0.000000000	0.000000000	0.123322000
N	0.000000000	0.000000000	3.113936000
F	1.140595000	-0.068727000	-0.667242000
F	-1.140595000	0.068727000	-0.667242000

H	-0.069286000	4.401730000	2.007019000
C	0.000000000	3.125846000	-0.575649000
C	0.000000000	-3.125846000	-0.575649000
H	0.069286000	-4.401730000	2.007019000
S	0.568355000	-4.762826000	-0.798626000
C	0.236988000	-4.704498000	-2.481600000
C	-0.283584000	-3.500463000	-2.858002000
C	-0.420744000	-2.597283000	-1.773698000
C	0.420744000	2.597283000	-1.773698000
C	0.283584000	3.500463000	-2.858002000
C	-0.236988000	4.704498000	-2.481600000
S	-0.568355000	4.762826000	-0.798626000
H	0.450720000	-5.562532000	-3.103911000
H	-0.566127000	-3.263887000	-3.876454000
H	-0.838446000	-1.605079000	-1.859383000
H	0.838446000	1.605079000	-1.859383000
H	0.566127000	3.263887000	-3.876454000
H	-0.450720000	5.562532000	-3.103911000
H	-0.054920000	2.655901000	4.067208000
H	0.054920000	-2.655901000	4.067208000

52

B1 GS CAM-B3LYP/6-31G(d)

N	-0.009854000	1.227162000	-1.663451000
C	0.010181000	1.140645000	-0.272406000
C	0.000000000	2.469379000	0.265252000
C	-0.035322000	3.316474000	-0.833683000
C	-0.036530000	2.526702000	-2.000733000
N	0.009854000	-1.227162000	-1.663451000
C	-0.010181000	-1.140645000	-0.272406000

C	0.000000000	-2.469379000	0.265252000
C	0.035322000	-3.316474000	-0.833683000
C	0.036530000	-2.526702000	-2.000733000
B	0.000000000	0.000000000	-2.610470000
N	0.000000000	0.000000000	0.386105000
F	1.138249000	0.008296000	-3.409506000
F	-1.138249000	-0.008296000	-3.409506000
C	0.008305000	2.854079000	1.679145000
H	-0.030655000	4.396118000	-0.819207000
H	0.030655000	-4.396118000	-0.819207000
C	-0.008305000	-2.854079000	1.679145000
C	0.563556000	2.425472000	3.998490000
C	0.555603000	2.024994000	2.668288000
C	-0.514975000	4.489687000	3.392831000
C	-0.563556000	-2.425472000	3.998490000
C	0.030461000	3.657175000	4.366541000
C	-0.525724000	4.092236000	2.063161000
C	-0.555603000	-2.024994000	2.668288000
C	-0.030461000	-3.657175000	4.366541000
H	0.039395000	3.967320000	5.406677000
H	-0.975845000	-1.067408000	2.388089000
H	-0.039395000	-3.967320000	5.406677000
H	-0.938231000	5.449786000	3.670591000
H	-0.994690000	-1.772719000	4.751072000
H	-0.970724000	4.738779000	1.313890000
C	0.525724000	-4.092236000	2.063161000
C	0.514975000	-4.489687000	3.392831000
H	0.994690000	1.772719000	4.751072000
H	0.975845000	1.067408000	2.388089000

H	0.970724000	-4.738779000	1.313890000
H	0.938231000	-5.449786000	3.670591000
C	0.053648000	-2.965390000	-3.429378000
H	-0.814915000	-2.519865000	-3.927956000
H	0.925956000	-2.507621000	-3.910047000
C	0.066625000	-4.477884000	-3.620618000
H	-0.820789000	-4.944068000	-3.182257000
H	0.950247000	-4.931332000	-3.161700000
H	0.080770000	-4.720850000	-4.685965000
C	-0.053648000	2.965390000	-3.429378000
H	0.814915000	2.519865000	-3.927956000
H	-0.925956000	2.507621000	-3.910047000
C	-0.066625000	4.477884000	-3.620618000
H	0.820789000	4.944068000	-3.182257000
H	-0.950247000	4.931332000	-3.161700000
H	-0.080770000	4.720850000	-4.685965000

46

B2 GS CAM-B3LYP/6-31G(d)

N	-0.030221000	1.226288000	-1.603412000
C	0.000000000	1.138014000	-0.214168000
C	-0.007948000	2.468077000	0.321486000
C	-0.062282000	3.318135000	-0.777591000
C	-0.070770000	2.527604000	-1.940784000
N	0.030221000	-1.226288000	-1.603412000
C	0.000000000	-1.138014000	-0.214168000
C	0.007948000	-2.468077000	0.321486000
C	0.062282000	-3.318135000	-0.777591000
C	0.070770000	-2.527604000	-1.940784000
B	0.000000000	0.000000000	-2.551192000

N	0.000000000	0.000000000	0.448506000
F	1.137710000	0.027787000	-3.350428000
F	-1.137710000	-0.027787000	-3.350428000
H	-0.072899000	4.398152000	-0.761073000
H	0.072899000	-4.398152000	-0.761073000
C	-0.033115000	-2.825593000	1.721335000
S	0.378254000	-4.439334000	2.237228000
C	-0.392972000	-2.054630000	2.800192000
C	0.064833000	-4.051794000	3.884265000
C	-0.334783000	-2.756621000	4.033845000
H	-0.700058000	-1.022515000	2.698950000
H	0.196207000	-4.802570000	4.651057000
H	-0.584662000	-2.315135000	4.990924000
C	0.033115000	2.825593000	1.721335000
S	-0.378254000	4.439334000	2.237228000
C	0.392972000	2.054630000	2.800192000
C	-0.064833000	4.051794000	3.884265000
C	0.334783000	2.756621000	4.033845000
H	0.700058000	1.022515000	2.698950000
H	-0.196207000	4.802570000	4.651057000
H	0.584662000	2.315135000	4.990924000
C	0.106562000	-2.964360000	-3.369494000
H	-0.762668000	-2.528178000	-3.875167000
H	0.977785000	-2.495839000	-3.841584000
C	0.138519000	-4.476363000	-3.562009000
H	-0.747822000	-4.952991000	-3.132802000
H	1.022809000	-4.920227000	-3.095079000
H	0.165690000	-4.717922000	-4.627399000
C	-0.106562000	2.964360000	-3.369494000

H	0.762668000	2.528178000	-3.875167000
H	-0.977785000	2.495839000	-3.841584000
C	-0.138519000	4.476363000	-3.562009000
H	0.747822000	4.952991000	-3.132802000
H	-1.022809000	4.920227000	-3.095079000
H	-0.165690000	4.717922000	-4.627399000

46

B3 GS CAM-B3LYP/6-31G(d)

N	-0.006957000	1.225970000	-1.355798000
C	0.019222000	1.136485000	0.033590000
C	0.036080000	2.466260000	0.562802000
C	0.000000000	3.321263000	-0.532463000
C	-0.021445000	2.529148000	-1.693740000
N	0.006957000	-1.225970000	-1.355798000
C	-0.019222000	-1.136485000	0.033590000
C	-0.036080000	-2.466260000	0.562802000
C	0.000000000	-3.321263000	-0.532463000
C	0.021445000	-2.529148000	-1.693740000
B	0.000000000	0.000000000	-2.303429000
N	0.000000000	0.000000000	0.700286000
F	1.137974000	0.006627000	-3.103490000
F	-1.137974000	-0.006627000	-3.103490000
H	0.001434000	4.399992000	-0.505051000
H	-0.001434000	-4.399992000	-0.505051000
C	-0.090966000	-2.833815000	1.948749000
O	0.054557000	-4.163286000	2.231583000
C	-0.290766000	-2.141379000	3.111072000
C	-0.048731000	-4.302750000	3.572732000
C	-0.260826000	-3.098423000	4.164852000

H	-0.456445000	-1.078334000	3.187155000
H	0.050399000	-5.308259000	3.951211000
H	-0.384189000	-2.913886000	5.221761000
C	0.090966000	2.833815000	1.948749000
O	-0.054557000	4.163286000	2.231583000
C	0.290766000	2.141379000	3.111072000
C	0.048731000	4.302750000	3.572732000
C	0.260826000	3.098423000	4.164852000
H	0.456445000	1.078334000	3.187155000
H	-0.050399000	5.308259000	3.951211000
H	0.384189000	2.913886000	5.221761000
C	-0.049911000	2.964431000	-3.123144000
H	0.810668000	2.511450000	-3.628867000
H	-0.930022000	2.512151000	-3.594586000
C	-0.053142000	4.476596000	-3.316890000
H	0.841796000	4.936509000	-2.887321000
H	-0.928909000	4.937382000	-2.850313000
H	-0.075350000	4.718060000	-4.382448000
C	0.049911000	-2.964431000	-3.123144000
H	-0.810668000	-2.511450000	-3.628867000
H	0.930022000	-2.512151000	-3.594586000
C	0.053142000	-4.476596000	-3.316890000
H	-0.841796000	-4.936509000	-2.887321000
H	0.928909000	-4.937382000	-2.850313000
H	0.075350000	-4.718060000	-4.382448000

50

B4 GS CAM-B3LYP/6-31G(d)

N	0.019871000	1.227853000	-1.654123000
C	0.000000000	1.139609000	-0.264242000

C	0.019017000	2.466816000	0.273406000
C	0.058093000	3.316795000	-0.821169000
C	0.054522000	2.526852000	-1.990447000
N	-0.019871000	-1.227853000	-1.654123000
C	0.000000000	-1.139609000	-0.264242000
C	-0.019017000	-2.466816000	0.273406000
C	-0.058093000	-3.316795000	-0.821169000
C	-0.054522000	-2.526852000	-1.990447000
B	0.000000000	0.000000000	-2.603183000
N	0.000000000	0.000000000	0.396077000
F	1.138342000	-0.017663000	-3.399169000
F	-1.138342000	0.017663000	-3.399169000
H	0.059396000	4.396408000	-0.805468000
H	-0.059396000	-4.396408000	-0.805468000
C	-0.016395000	-2.837731000	1.688430000
C	-0.581990000	-4.053561000	2.093885000
C	0.542574000	-2.030941000	2.685348000
N	-0.610696000	-4.484206000	3.351717000
C	0.512351000	-2.467615000	4.001068000
C	-0.067516000	-3.698248000	4.285017000
H	0.993857000	-1.081358000	2.425794000
H	0.940193000	-1.868269000	4.796961000
H	-0.097274000	-4.070983000	5.306018000
H	-1.050271000	-4.701031000	1.356102000
C	0.016395000	2.837731000	1.688430000
C	0.581990000	4.053561000	2.093885000
C	-0.542574000	2.030941000	2.685348000
N	0.610696000	4.484206000	3.351717000
C	-0.512351000	2.467615000	4.001068000

C	0.067516000	3.698248000	4.285017000
H	1.050271000	4.701031000	1.356102000
H	-0.993857000	1.081358000	2.425794000
H	-0.940193000	1.868269000	4.796961000
H	0.097274000	4.070983000	5.306018000
C	-0.074220000	-2.967502000	-3.418057000
H	-0.943802000	-2.504778000	-3.898963000
H	0.797154000	-2.527629000	-3.916750000
C	-0.096452000	-4.480155000	-3.607175000
H	-0.983025000	-4.927653000	-3.148194000
H	0.788194000	-4.951260000	-3.168561000
H	-0.111970000	-4.723770000	-4.672275000
C	0.074220000	2.967502000	-3.418057000
H	0.943802000	2.504778000	-3.898963000
H	-0.797154000	2.527629000	-3.916750000
C	0.096452000	4.480155000	-3.607175000
H	0.983025000	4.927653000	-3.148194000
H	-0.788194000	4.951260000	-3.168561000
H	0.111970000	4.723770000	-4.672275000

50

B5 GS CAM-B3LYP/6-31G(d)

N	0.007339000	1.228801000	-1.659206000
C	-0.009547000	1.139996000	-0.269722000
C	0.000000000	2.467936000	0.266525000
C	0.028609000	3.317353000	-0.826711000
C	0.028196000	2.526931000	-1.997157000
N	-0.007339000	-1.228801000	-1.659206000
C	0.009547000	-1.139996000	-0.269722000
C	0.000000000	-2.467936000	0.266525000

C	-0.028609000	-3.317353000	-0.826711000
C	-0.028196000	-2.526931000	-1.997157000
B	0.000000000	0.000000000	-2.609111000
N	0.000000000	0.000000000	0.389339000
F	1.138683000	-0.006530000	-3.402906000
F	-1.138683000	0.006530000	-3.402906000
H	0.022731000	4.396967000	-0.810611000
H	-0.022731000	-4.396967000	-0.810611000
C	0.006078000	-2.847997000	1.682065000
C	-0.516178000	-4.081279000	2.084711000
C	0.538020000	-2.024968000	2.679590000
C	-0.483101000	-4.425986000	3.427836000
C	0.518785000	-2.465242000	3.996211000
N	0.022535000	-3.642286000	4.383433000
H	0.960528000	-1.059573000	2.434431000
H	-0.888101000	-5.381775000	3.751866000
H	0.929943000	-1.834920000	4.781298000
H	-0.959765000	-4.759914000	1.364974000
C	-0.006078000	2.847997000	1.682065000
C	0.516178000	4.081279000	2.084711000
C	-0.538020000	2.024968000	2.679590000
C	0.483101000	4.425986000	3.427836000
C	-0.518785000	2.465242000	3.996211000
N	-0.022535000	3.642286000	4.383433000
H	0.959765000	4.759914000	1.364974000
H	-0.960528000	1.059573000	2.434431000
H	0.888101000	5.381775000	3.751866000
H	-0.929943000	1.834920000	4.781298000
C	0.040843000	2.967676000	-3.424591000

H	0.912230000	2.510810000	-3.907772000
H	-0.829025000	2.521808000	-3.920584000
C	0.052069000	4.480485000	-3.613499000
H	0.937053000	4.934074000	-3.157467000
H	-0.834556000	4.945463000	-3.172393000
H	0.062623000	4.723912000	-4.678653000
C	-0.040843000	-2.967676000	-3.424591000
H	-0.912230000	-2.510810000	-3.907772000
H	0.829025000	-2.521808000	-3.920584000
C	-0.052069000	-4.480485000	-3.613499000
H	-0.937053000	-4.934074000	-3.157467000
H	0.834556000	-4.945463000	-3.172393000
H	-0.062623000	-4.723912000	-4.678653000

50

B6 GS CAM-B3LYP/6-31G(d)

N	0.000000000	1.227725000	-1.629768000
C	-0.024476000	1.141856000	-0.239027000
C	-0.034267000	2.474049000	0.289779000
C	0.002094000	3.319319000	-0.805195000
C	0.016261000	2.526237000	-1.971365000
N	0.000000000	-1.227725000	-1.629768000
C	0.024476000	-1.141856000	-0.239027000
C	0.034267000	-2.474049000	0.289779000
C	-0.002094000	-3.319319000	-0.805195000
C	-0.016261000	-2.526237000	-1.971365000
B	0.000000000	0.000000000	-2.577292000
N	0.000000000	0.000000000	0.418334000
F	1.138527000	-0.000148000	-3.374067000
F	-1.138527000	0.000148000	-3.374067000

H	0.007532000	4.397248000	-0.771828000
H	-0.007532000	-4.397248000	-0.771828000
C	0.073693000	-2.901659000	1.694250000
N	-0.221487000	-4.195009000	1.909866000
C	0.421822000	-2.035604000	2.734423000
C	-0.188978000	-4.648226000	3.158723000
C	0.447605000	-2.524606000	4.033788000
C	0.134591000	-3.857912000	4.258430000
H	0.670026000	-1.005221000	2.518013000
H	-0.434690000	-5.699223000	3.293248000
H	0.715014000	-1.870806000	4.857631000
H	0.143283000	-4.283111000	5.255708000
C	-0.073693000	2.901659000	1.694250000
N	0.221487000	4.195009000	1.909866000
C	-0.421822000	2.035604000	2.734423000
C	0.188978000	4.648226000	3.158723000
C	-0.447605000	2.524606000	4.033788000
C	-0.134591000	3.857912000	4.258430000
H	-0.670026000	1.005221000	2.518013000
H	0.434690000	5.699223000	3.293248000
H	-0.715014000	1.870806000	4.857631000
H	-0.143283000	4.283111000	5.255708000
C	-0.041217000	-2.961626000	-3.400483000
H	-0.921193000	-2.510661000	-3.873492000
H	0.819612000	-2.507386000	-3.904680000
C	-0.041935000	-4.473958000	-3.593391000
H	-0.918453000	-4.935653000	-3.129273000
H	0.852260000	-4.932498000	-3.160971000
H	-0.060716000	-4.715483000	-4.658971000

C	0.041217000	2.961626000	-3.400483000
H	0.921193000	2.510661000	-3.873492000
H	-0.819612000	2.507386000	-3.904680000
C	0.041935000	4.473958000	-3.593391000
H	0.918453000	4.935653000	-3.129273000
H	-0.852260000	4.932498000	-3.160971000
H	0.060716000	4.715483000	-4.658971000

32

B7 GS CAM-B3LYP/6-31G(d)

N	0.000000000	1.228224000	0.257501000
C	0.000000000	1.137504000	1.651845000
C	0.000000000	2.455331000	2.177839000
C	0.000000000	3.316540000	1.105037000
C	0.000000000	2.526217000	-0.074941000
N	0.000000000	-1.228224000	0.257501000
C	0.000000000	-1.137504000	1.651845000
C	0.000000000	-2.455331000	2.177839000
C	0.000000000	-3.316540000	1.105037000
C	0.000000000	-2.526217000	-0.074941000
B	0.000000000	0.000000000	-0.692320000
N	0.000000000	0.000000000	2.318202000
F	1.138800000	0.000000000	-1.488062000
F	-1.138800000	0.000000000	-1.488062000
H	0.000000000	4.396083000	1.128776000
H	0.000000000	-4.396083000	1.128776000
C	0.000000000	-2.972547000	-1.501292000
H	-0.870636000	-2.523953000	-1.993613000
H	0.870636000	-2.523953000	-1.993613000
C	0.000000000	-4.486285000	-1.683911000

H	-0.885642000	-4.943591000	-1.232844000
H	0.885642000	-4.943591000	-1.232844000
H	0.000000000	-4.735168000	-2.747947000
C	0.000000000	2.972547000	-1.501292000
H	0.870636000	2.523953000	-1.993613000
H	-0.870636000	2.523953000	-1.993613000
C	0.000000000	4.486285000	-1.683911000
H	0.885642000	4.943591000	-1.232844000
H	-0.885642000	4.943591000	-1.232844000
H	0.000000000	4.735168000	-2.747947000
H	0.000000000	-2.694759000	3.231765000
H	0.000000000	2.694759000	3.231765000

60

? GS CAM-B3LYP/6-31G(d)

N	-0.689593000	-1.246236000	0.151519000
C	0.700779000	-1.149215000	0.175816000
C	1.263929000	-2.462538000	0.077679000
C	0.184268000	-3.317755000	-0.039196000
C	-1.003685000	-2.553456000	0.019671000
N	-0.680975000	1.242987000	0.110834000
C	0.709564000	1.136253000	0.100929000
C	1.279160000	2.443048000	-0.041686000
C	0.201709000	3.304340000	-0.131538000
C	-0.989614000	2.550070000	-0.034173000
B	-1.609619000	0.003744000	0.286358000
N	1.355726000	-0.008437000	0.155580000
F	-2.152615000	0.024809000	1.579243000
F	-2.602685000	-0.007801000	-0.667936000
H	0.213235000	-4.395749000	-0.098808000

H	0.235792000	4.376911000	-0.252498000
C	-2.339891000	3.122751000	-0.098528000
C	-3.405654000	2.646301000	0.674816000
C	-2.550567000	4.228317000	-0.934775000
C	-4.649190000	3.260221000	0.602900000
C	-3.797808000	4.832452000	-1.008560000
C	-4.851950000	4.350311000	-0.238114000
H	-3.253306000	1.807708000	1.340663000
H	-1.736485000	4.600516000	-1.547697000
H	-5.465001000	2.885739000	1.212971000
H	-3.946660000	5.679546000	-1.670290000
H	-5.827184000	4.823686000	-0.291818000
C	-2.356547000	-3.118481000	-0.043058000
C	-2.568055000	-4.241292000	-0.855553000
C	-3.424585000	-2.616895000	0.711098000
C	-3.818980000	-4.838249000	-0.925545000
C	-4.671790000	-3.223544000	0.643214000
C	-4.875524000	-4.331062000	-0.174674000
H	-1.751026000	-4.632867000	-1.452394000
H	-3.270816000	-1.765029000	1.359787000
H	-3.968905000	-5.699348000	-1.568684000
H	-5.489727000	-2.829960000	1.238208000
H	-5.853679000	-4.798710000	-0.225366000
C	2.685536000	-2.816062000	0.076097000
C	3.108118000	-3.986283000	-0.569325000
C	3.642703000	-2.023360000	0.724026000
C	4.446739000	-4.352779000	-0.569954000
C	4.982057000	-2.392308000	0.720080000
C	5.389317000	-3.556300000	0.075015000

H	2.382280000	-4.601565000	-1.091090000
H	3.331008000	-1.120079000	1.233220000
H	4.755978000	-5.259643000	-1.079820000
H	5.710417000	-1.769394000	1.229680000
H	6.436388000	-3.842250000	0.074707000
C	2.701377000	2.799836000	-0.074285000
C	3.684892000	1.899685000	-0.506979000
C	3.096980000	4.085758000	0.319967000
C	5.020926000	2.279252000	-0.540884000
C	4.432547000	4.461455000	0.284208000
C	5.400745000	3.558789000	-0.147277000
H	3.396699000	0.903370000	-0.816662000
H	2.352195000	4.789525000	0.676961000
H	5.769099000	1.570579000	-0.882057000
H	4.718790000	5.459927000	0.599132000
H	6.445568000	3.851537000	-0.175684000

54

C2 GS CAM-B3LYP/6-31G(d)

N	0.301594000	1.295213000	0.157876000
C	-0.995257000	0.785320000	0.192927000
C	-1.923059000	1.875353000	0.098208000
C	-1.147609000	3.013515000	-0.040783000
C	0.211108000	2.636005000	0.009307000
N	1.045995000	-1.075960000	0.150148000
C	-0.309727000	-1.390404000	0.140785000
C	-0.460769000	-2.805289000	-0.009647000
C	0.828299000	-3.307650000	-0.105142000
C	1.733138000	-2.233040000	-0.000561000
B	1.557537000	0.384651000	0.308133000

N	-1.278058000	-0.499547000	0.183291000
F	2.079306000	0.549486000	1.599712000
F	2.502606000	0.683850000	-0.648211000
H	-1.498159000	4.032929000	-0.111947000
H	1.116529000	-4.341851000	-0.219120000
C	-1.692546000	-3.560714000	-0.060365000
S	-3.271444000	-2.828901000	-0.150726000
C	-1.801063000	-4.931359000	-0.059982000
C	-4.038956000	-4.368414000	-0.187033000
C	-3.140535000	-5.392953000	-0.130678000
H	-0.942391000	-5.589436000	-0.001541000
H	-5.116869000	-4.431701000	-0.246829000
H	-3.419331000	-6.439515000	-0.138260000
C	-3.364639000	1.782690000	0.140368000
S	-4.355637000	3.090712000	-0.447620000
C	-4.148922000	0.770840000	0.636704000
C	-5.800246000	2.258389000	-0.022052000
C	-5.539848000	1.044026000	0.541120000
H	-3.733818000	-0.132183000	1.062642000
H	-6.762567000	2.711581000	-0.215719000
H	-6.312270000	0.365503000	0.882150000
C	1.334198000	3.576758000	-0.077540000
C	2.506011000	3.431817000	0.675183000
C	1.197445000	4.694502000	-0.912551000
C	3.515145000	4.381224000	0.583605000
C	2.213226000	5.635539000	-1.006256000
C	3.376046000	5.481769000	-0.256945000
H	2.615823000	2.586405000	1.340974000
H	0.298265000	4.812227000	-1.508151000

H	4.415591000	4.261868000	1.177646000
H	2.096888000	6.488689000	-1.666685000
H	4.170074000	6.218497000	-0.326220000
C	3.193765000	-2.370626000	-0.057504000
C	4.059943000	-1.594699000	0.722444000
C	3.734605000	-3.359355000	-0.891396000
C	5.431442000	-1.803476000	0.659762000
C	5.106628000	-3.558340000	-0.955371000
C	5.960204000	-2.780731000	-0.178184000
H	3.656406000	-0.841247000	1.385374000
H	3.075103000	-3.959087000	-1.509727000
H	6.091572000	-1.200050000	1.274650000
H	5.509392000	-4.320078000	-1.615038000
H	7.033281000	-2.936929000	-0.224558000

54

? GS CAM-B3LYP/6-31G(d)

N	0.482344000	-1.209987000	0.125012000
C	1.496218000	-0.255708000	0.163318000
C	2.757313000	-0.923596000	0.063761000
C	2.467003000	-2.269064000	-0.082444000
C	1.065772000	-2.422024000	-0.029910000
N	-1.084197000	0.721643000	0.146868000
C	0.061323000	1.514211000	0.123502000
C	-0.331280000	2.882156000	-0.017778000
C	-1.714062000	2.878584000	-0.081945000
C	-2.154042000	1.542866000	0.021354000
B	-1.018581000	-0.827925000	0.290735000
N	1.290442000	1.044256000	0.159182000
F	-1.430926000	-1.184670000	1.583256000

F	-1.797020000	-1.444408000	-0.663922000
H	3.177931000	-3.076473000	-0.169851000
H	-2.356805000	3.740746000	-0.173106000
C	0.538578000	4.021519000	-0.096649000
O	-0.068486000	5.246291000	-0.098395000
C	1.892005000	4.184581000	-0.201566000
C	0.906507000	6.177901000	-0.199088000
C	2.128174000	5.587342000	-0.266376000
H	2.618014000	3.388414000	-0.245967000
H	0.577488000	7.205405000	-0.208683000
H	3.080954000	6.088105000	-0.354499000
C	4.054365000	-0.311104000	0.113401000
O	5.115850000	-1.107792000	-0.212217000
C	4.507573000	0.931115000	0.461635000
C	6.235320000	-0.361571000	-0.074601000
C	5.925597000	0.896000000	0.335765000
H	3.893048000	1.755588000	0.787450000
H	7.164658000	-0.859297000	-0.304178000
H	6.620963000	1.699298000	0.529289000
C	0.370396000	-3.711271000	-0.124570000
C	-0.771039000	-4.015741000	0.627218000
C	0.910629000	-4.693652000	-0.966207000
C	-1.356862000	-5.270851000	0.528050000
C	0.315866000	-5.943539000	-1.067177000
C	-0.820622000	-6.236358000	-0.318858000
H	-1.185310000	-3.275763000	1.298746000
H	1.789195000	-4.465882000	-1.560631000
H	-2.236996000	-5.497309000	1.121393000
H	0.739584000	-6.688751000	-1.732558000

H	-1.285290000	-7.214368000	-0.393949000
C	-3.562983000	1.131435000	-0.014816000
C	-4.069568000	0.082124000	0.761751000
C	-4.444142000	1.860724000	-0.825750000
C	-5.422416000	-0.228417000	0.718150000
C	-5.793795000	1.540738000	-0.870977000
C	-6.287907000	0.494501000	-0.097304000
H	-3.406179000	-0.477114000	1.407458000
H	-4.062560000	2.668596000	-1.441054000
H	-5.803429000	-1.039497000	1.330490000
H	-6.459706000	2.108053000	-1.513119000
H	-7.343704000	0.244791000	-0.128802000

58

C4 GS CAM-B3LYP/6-31G(d)

N	-0.685112000	-1.245851000	0.152630000
C	0.704238000	-1.149398000	0.176750000
C	1.265037000	-2.462542000	0.082643000
C	0.188227000	-3.319459000	-0.030322000
C	-1.000684000	-2.552792000	0.025182000
N	-0.671999000	1.245301000	0.110323000
C	0.717074000	1.133867000	0.101507000
C	1.289163000	2.437711000	-0.043766000
C	0.218184000	3.304139000	-0.136736000
C	-0.977099000	2.552543000	-0.037327000
B	-1.604877000	0.006880000	0.285370000
N	1.363181000	-0.010833000	0.156340000
F	-2.146893000	0.029774000	1.576909000
F	-2.594037000	-0.003472000	-0.671195000
H	0.216573000	-4.397673000	-0.085516000

H	0.256368000	4.376153000	-0.261252000
C	2.712962000	2.777225000	-0.074416000
C	3.134578000	4.044212000	0.348939000
C	3.700298000	1.893503000	-0.522595000
N	4.399512000	4.454466000	0.341199000
C	5.023121000	2.309522000	-0.529570000
C	5.323666000	3.594929000	-0.095500000
H	3.428487000	0.901338000	-0.860222000
H	5.812033000	1.650523000	-0.874634000
H	6.350783000	3.951715000	-0.098261000
C	2.687111000	-2.804400000	0.080114000
C	3.129318000	-3.956599000	-0.581935000
C	3.654015000	-2.029507000	0.728925000
N	4.396939000	-4.356516000	-0.622460000
C	4.979758000	-2.433848000	0.687248000
C	5.301798000	-3.602079000	0.006733000
H	3.364614000	-1.130256000	1.258603000
H	5.753798000	-1.858639000	1.182855000
H	6.331372000	-3.949156000	-0.034955000
C	-2.352960000	-3.117506000	-0.037054000
C	-3.422815000	-2.610455000	0.711033000
C	-2.562290000	-4.245284000	-0.843477000
C	-4.670070000	-3.216680000	0.642812000
C	-3.813447000	-4.841356000	-0.914018000
C	-4.871842000	-4.328717000	-0.169426000
H	-3.270513000	-1.755713000	1.356180000
H	-1.743993000	-4.641221000	-1.435642000
H	-5.489506000	-2.819333000	1.233126000
H	-3.962101000	-5.706084000	-1.552469000

H	-5.850144000	-4.795956000	-0.220413000
C	-2.324688000	3.129455000	-0.102726000
C	-3.393307000	2.654101000	0.667535000
C	-2.530035000	4.237391000	-0.937398000
C	-4.634894000	3.271433000	0.593807000
C	-3.775566000	4.844441000	-1.013200000
C	-4.832627000	4.363375000	-0.246027000
H	-3.244697000	1.814714000	1.333167000
H	-1.713858000	4.608906000	-1.547893000
H	-5.453037000	2.898340000	1.201500000
H	-3.920802000	5.693056000	-1.673686000
H	-5.806465000	4.839378000	-0.301165000
H	2.414535000	-4.574919000	-1.120214000
H	2.404309000	4.754392000	0.729984000

58

? GS CAM-B3LYP/6-31G(d)

N	-0.688594000	-1.246580000	0.150803000
C	0.700399000	-1.149670000	0.172572000
C	1.259572000	-2.462879000	0.071288000
C	0.183929000	-3.318804000	-0.041385000
C	-1.006055000	-2.552294000	0.021266000
N	-0.675601000	1.245985000	0.111968000
C	0.713147000	1.134634000	0.105141000
C	1.283534000	2.440069000	-0.034133000
C	0.213606000	3.305234000	-0.127969000
C	-0.982625000	2.552262000	-0.034195000
B	-1.609008000	0.006593000	0.285815000
N	1.358180000	-0.010483000	0.156131000
F	-2.148460000	0.027879000	1.577302000

F	-2.596893000	-0.002325000	-0.670972000
H	0.213094000	-4.396659000	-0.102979000
H	0.251843000	4.377804000	-0.247564000
C	2.707821000	2.789785000	-0.064551000
C	3.696936000	1.897201000	-0.488462000
C	3.125385000	4.066816000	0.323390000
C	5.020419000	2.317063000	-0.501455000
C	4.474394000	4.385029000	0.270005000
N	5.422123000	3.535577000	-0.133707000
H	2.412688000	4.801662000	0.680308000
H	5.799149000	1.632638000	-0.829910000
H	4.809693000	5.374644000	0.571307000
H	3.440704000	0.894352000	-0.802705000
C	2.682256000	-2.814742000	0.066735000
C	3.649988000	-2.030619000	0.701577000
C	3.120676000	-3.979806000	-0.569739000
C	4.976364000	-2.439857000	0.665486000
C	4.470330000	-4.299190000	-0.543126000
N	5.398222000	-3.551940000	0.059830000
H	3.375243000	-1.119657000	1.216839000
H	2.423900000	-4.623051000	-1.095309000
H	5.739457000	-1.839703000	1.155521000
H	4.823331000	-5.201850000	-1.036129000
C	-2.358065000	-3.116729000	-0.039138000
C	-3.426715000	-2.610168000	0.711123000
C	-2.568528000	-4.243772000	-0.846474000
C	-4.674092000	-3.216077000	0.643928000
C	-3.819922000	-4.839224000	-0.916115000
C	-4.877116000	-4.327053000	-0.169453000

H	-3.273424000	-1.756539000	1.357453000
H	-1.751370000	-4.639431000	-1.440373000
H	-5.492577000	-2.819401000	1.235947000
H	-3.969738000	-5.703132000	-1.555352000
H	-5.855567000	-4.794023000	-0.219618000
C	-2.329801000	3.128827000	-0.100044000
C	-3.399697000	2.651052000	0.667080000
C	-2.533441000	4.239028000	-0.932259000
C	-4.641204000	3.268199000	0.592308000
C	-3.779067000	4.845538000	-1.009313000
C	-4.837449000	4.362017000	-0.245480000
H	-3.252110000	1.810666000	1.331647000
H	-1.716190000	4.612491000	-1.540127000
H	-5.460419000	2.893609000	1.197567000
H	-3.923347000	5.695692000	-1.667973000
H	-5.811321000	4.837812000	-0.301442000

58

C6 GS CAM-B3LYP/6-31G(d)

N	-0.650010000	-1.255571000	0.137384000
C	0.738819000	-1.146866000	0.170263000
C	1.305858000	-2.456791000	0.060666000
C	0.239158000	-3.317797000	-0.082136000
C	-0.955614000	-2.562627000	-0.016803000
N	-0.664176000	1.234686000	0.134848000
C	0.727326000	1.141928000	0.116994000
C	1.274859000	2.458602000	-0.021865000
C	0.192275000	3.308486000	-0.095160000
C	-0.990440000	2.539164000	0.003283000
B	-1.581385000	-0.016022000	0.292408000

N	1.382910000	0.001311000	0.159965000
F	-2.121496000	-0.022412000	1.585608000
F	-2.573397000	-0.020186000	-0.661975000
H	0.296998000	-4.390700000	-0.179518000
H	0.235880000	4.382151000	-0.187921000
C	2.683711000	2.873734000	-0.084306000
N	2.892118000	4.197135000	0.023239000
C	3.732811000	1.969033000	-0.265180000
C	4.141605000	4.645177000	-0.035883000
C	5.032962000	2.453811000	-0.322674000
C	5.249904000	3.819262000	-0.204178000
H	3.522660000	0.912736000	-0.363209000
H	4.269390000	5.721373000	0.056232000
H	5.863795000	1.769876000	-0.462916000
H	6.247614000	4.241666000	-0.243986000
C	2.722929000	-2.840639000	0.082805000
N	2.997942000	-4.058005000	-0.414531000
C	3.713529000	-2.013492000	0.618519000
C	4.261760000	-4.469750000	-0.404186000
C	5.029461000	-2.457158000	0.617448000
C	5.316874000	-3.709898000	0.093788000
H	3.448165000	-1.049234000	1.031088000
H	4.446255000	-5.459417000	-0.815812000
H	5.816542000	-1.832911000	1.027815000
H	6.328979000	-4.098032000	0.073736000
C	-2.303473000	-3.135414000	-0.097386000
C	-3.380877000	-2.652410000	0.655847000
C	-2.500062000	-4.248394000	-0.927254000
C	-4.622986000	-3.266818000	0.569204000

C	-3.746202000	-4.852426000	-1.016362000
C	-4.812261000	-4.363320000	-0.266845000
H	-3.238220000	-1.810160000	1.319404000
H	-1.675214000	-4.626447000	-1.521968000
H	-5.448272000	-2.888192000	1.163697000
H	-3.884878000	-5.705231000	-1.672877000
H	-5.786627000	-4.836986000	-0.332233000
C	-2.346159000	3.098521000	-0.040945000
C	-3.404542000	2.595797000	0.726187000
C	-2.570091000	4.220288000	-0.852257000
C	-4.653844000	3.199403000	0.671688000
C	-3.823160000	4.813647000	-0.909116000
C	-4.869887000	4.304929000	-0.145574000
H	-3.242055000	1.745843000	1.374822000
H	-1.761239000	4.614027000	-1.458473000
H	-5.463696000	2.804776000	1.276929000
H	-3.982356000	5.673472000	-1.551685000
H	-5.849700000	4.770102000	-0.185706000

40

C7 GS CAM-B3LYP/6-31G(d)

N	1.175143000	0.106597000	1.246005000
C	2.568330000	0.099124000	1.140042000
C	3.121929000	-0.024827000	2.439755000
C	2.068775000	-0.114216000	3.312064000
C	0.867255000	-0.023644000	2.552572000
N	1.175143000	0.106597000	-1.246005000
C	2.568330000	0.099124000	-1.140042000
C	3.121929000	-0.024827000	-2.439755000
C	2.068775000	-0.114216000	-3.312064000

C	0.867255000	-0.023644000	-2.552572000
B	0.249470000	0.267405000	0.000000000
N	3.227755000	0.108808000	0.000000000
F	-0.282434000	1.563588000	0.000000000
F	-0.747328000	-0.681608000	0.000000000
H	2.109020000	-0.204884000	4.387452000
H	2.109020000	-0.204884000	-4.387452000
C	-0.480876000	-0.070148000	-3.128652000
C	-1.546586000	0.692726000	-2.634644000
C	-0.691122000	-0.879084000	-4.254779000
C	-2.789622000	0.636125000	-3.250763000
C	-1.938063000	-0.937955000	-4.860827000
C	-2.992080000	-0.178840000	-4.360608000
H	-1.393967000	1.340172000	-1.781821000
H	0.122845000	-1.482903000	-4.641804000
H	-3.605209000	1.237950000	-2.862785000
H	-2.086850000	-1.578727000	-5.723853000
H	-3.967016000	-0.220561000	-4.835762000
C	-0.480876000	-0.070148000	3.128652000
C	-0.691122000	-0.879084000	4.254779000
C	-1.546586000	0.692726000	2.634644000
C	-1.938063000	-0.937955000	4.860827000
C	-2.789622000	0.636125000	3.250763000
C	-2.992080000	-0.178840000	4.360608000
H	0.122845000	-1.482903000	4.641804000
H	-1.393967000	1.340172000	1.781821000
H	-2.086850000	-1.578727000	5.723853000
H	-3.605209000	1.237950000	2.862785000
H	-3.967016000	-0.220561000	4.835762000

H	4.180660000	-0.051035000	2.654230000
H	4.180660000	-0.051035000	-2.654230000

40

D1 GS CAM-B3LYP/6-31G(d)

N	0.014233000	1.224922000	2.317648000
C	-0.006763000	1.141431000	0.925849000
C	0.010391000	2.472446000	0.387661000
C	0.052431000	3.320036000	1.488287000
C	0.050082000	2.518833000	2.639830000
N	-0.014233000	-1.224922000	2.317648000
C	0.006763000	-1.141431000	0.925849000
C	-0.010391000	-2.472446000	0.387661000
C	-0.052431000	-3.320036000	1.488287000
C	-0.050082000	-2.518833000	2.639830000
B	0.000000000	0.000000000	3.274909000
N	0.000000000	0.000000000	0.268971000
F	-1.136473000	0.010247000	4.063098000
F	1.136473000	-0.010247000	4.063098000
C	0.002184000	2.855827000	-1.025911000
H	0.055176000	4.399954000	1.482192000
H	-0.055176000	-4.399954000	1.482192000
C	-0.002184000	-2.855827000	-1.025911000
C	-0.560978000	2.429220000	-3.343201000
C	-0.554131000	2.029988000	-2.012748000
C	0.536607000	4.485087000	-2.741376000
C	0.560978000	-2.429220000	-3.343201000
C	-0.017635000	3.655898000	-3.713030000
C	0.546089000	4.089277000	-1.411388000
C	0.554131000	-2.029988000	-2.012748000

C	0.017635000	-3.655898000	-3.713030000
H	-0.025670000	3.965024000	-4.753441000
H	0.983619000	-1.076827000	-1.731355000
H	0.025670000	-3.965024000	-4.753441000
H	0.967571000	5.441101000	-3.021091000
H	0.999281000	-1.779780000	-4.094400000
H	0.997849000	4.732727000	-0.663536000
C	-0.546089000	-4.089277000	-1.411388000
C	-0.536607000	-4.485087000	-2.741376000
H	-0.999281000	1.779780000	-4.094400000
H	-0.983619000	1.076827000	-1.731355000
H	-0.997849000	-4.732727000	-0.663536000
H	-0.967571000	-5.441101000	-3.021091000
H	0.064752000	2.832648000	3.675067000
H	-0.064752000	-2.832648000	3.675067000

34

D2 GS CAM-B3LYP/6-31G(d)

N	0.025255000	1.224093000	2.233063000
C	-0.005145000	1.138790000	0.843081000
C	0.000000000	2.471407000	0.306921000
C	0.054749000	3.322143000	1.408191000
C	0.065120000	2.520043000	2.555465000
N	-0.025255000	-1.224093000	2.233063000
C	0.005145000	-1.138790000	0.843081000
C	0.000000000	-2.471407000	0.306921000
C	-0.054749000	-3.322143000	1.408191000
C	-0.065120000	-2.520043000	2.555465000
B	0.000000000	0.000000000	3.190918000
N	0.000000000	0.000000000	0.182001000

F	-1.136054000	0.021013000	3.979657000
F	1.136054000	-0.021013000	3.979657000
H	0.064846000	4.402426000	1.400460000
H	-0.064846000	-4.402426000	1.400460000
C	0.043807000	-2.828088000	-1.092098000
S	-0.362620000	-4.442190000	-1.610111000
C	0.404037000	-2.054546000	-2.169575000
C	-0.047518000	-4.051544000	-3.255379000
C	0.349597000	-2.755083000	-3.403523000
H	0.709534000	-1.022029000	-2.067379000
H	-0.176065000	-4.801707000	-4.023270000
H	0.600158000	-2.312376000	-4.359776000
C	-0.043807000	2.828088000	-1.092098000
S	0.362620000	4.442190000	-1.610111000
C	-0.404037000	2.054546000	-2.169575000
C	0.047518000	4.051544000	-3.255379000
C	-0.349597000	2.755083000	-3.403523000
H	-0.709534000	1.022029000	-2.067379000
H	0.176065000	4.801707000	-4.023270000
H	-0.600158000	2.312376000	-4.359776000
H	0.091227000	2.833013000	3.590595000
H	-0.091227000	-2.833013000	3.590595000

34

D3 GS CAM-B3LYP/6-31G(d)

N	-0.022785000	1.223284000	-1.997076000
C	0.003986000	1.137411000	-0.606924000
C	0.000389000	2.470049000	-0.077821000
C	-0.048899000	3.324821000	-1.175625000
C	-0.057845000	2.520554000	-2.320612000

N	0.022785000	-1.223284000	-1.997076000
C	-0.003986000	-1.137411000	-0.606924000
C	-0.000389000	-2.470049000	-0.077821000
C	0.048899000	-3.324821000	-1.175625000
C	0.057845000	-2.520554000	-2.320612000
B	0.000000000	0.000000000	-2.954717000
N	0.000000000	0.000000000	0.058350000
F	1.135937000	0.019403000	-3.744504000
F	-1.135937000	-0.019403000	-3.744504000
H	-0.064728000	4.403782000	-1.156931000
H	0.064728000	-4.403782000	-1.156931000
C	-0.048933000	-2.839528000	1.306683000
O	0.112891000	-4.167422000	1.587369000
C	-0.255249000	-2.150724000	2.470529000
C	0.013414000	-4.309476000	2.927905000
C	-0.212237000	-3.108329000	3.522403000
H	-0.433589000	-1.089897000	2.548999000
H	0.125062000	-5.314137000	3.305162000
H	-0.336208000	-2.926851000	4.579710000
C	0.048933000	2.839528000	1.306683000
O	-0.112891000	4.167422000	1.587369000
C	0.255249000	2.150724000	2.470529000
C	-0.013414000	4.309476000	2.927905000
C	0.212237000	3.108329000	3.522403000
H	0.433589000	1.089897000	2.548999000
H	-0.125062000	5.314137000	3.305162000
H	0.336208000	2.926851000	4.579710000
H	-0.083520000	2.831149000	-3.356487000
H	0.083520000	-2.831149000	-3.356487000

38

D4 GS CAM-B3LYP/6-31G(d)

N	0.012128000	1.225990000	2.307541000
C	-0.007649000	1.140429000	0.916757000
C	0.004952000	2.469996000	0.378458000
C	0.041656000	3.320866000	1.474625000
C	0.042437000	2.519586000	2.628417000
N	-0.012128000	-1.225990000	2.307541000
C	0.007649000	-1.140429000	0.916757000
C	-0.004952000	-2.469996000	0.378458000
C	-0.041656000	-3.320866000	1.474625000
C	-0.042437000	-2.519586000	2.628417000
B	0.000000000	0.000000000	3.266976000
N	0.000000000	0.000000000	0.258324000
F	-1.136826000	0.008699000	4.051779000
F	1.136826000	-0.008699000	4.051779000
H	0.039281000	4.400758000	1.467341000
H	-0.039281000	-4.400758000	1.467341000
C	0.000902000	-2.839531000	-1.036433000
C	-0.563272000	-4.055612000	-1.443280000
C	0.560913000	-2.030884000	-2.031251000
N	-0.589720000	-4.484603000	-2.701469000
C	0.532991000	-2.466303000	-3.347303000
C	-0.045679000	-3.697152000	-3.633042000
H	1.012431000	-1.081642000	-1.770697000
H	0.961799000	-1.866128000	-4.141964000
H	-0.073455000	-4.068984000	-4.654376000
H	-1.032332000	-4.704059000	-0.706875000
C	-0.000902000	2.839531000	-1.036433000

C	0.563272000	4.055612000	-1.443280000
C	-0.560913000	2.030884000	-2.031251000
N	0.589720000	4.484603000	-2.701469000
C	-0.532991000	2.466303000	-3.347303000
C	0.045679000	3.697152000	-3.633042000
H	1.032332000	4.704059000	-0.706875000
H	-1.012431000	1.081642000	-1.770697000
H	-0.961799000	1.866128000	-4.141964000
H	0.073455000	4.068984000	-4.654376000
H	0.055269000	2.835283000	3.663009000
H	-0.055269000	-2.835283000	3.663009000

38

D5 GS CAM-B3LYP/6-31G(d)

N	0.013152000	1.226863000	2.312748000
C	-0.004289000	1.140919000	0.922230000
C	0.013507000	2.470990000	0.385469000
C	0.048600000	3.320914000	1.480114000
C	0.043611000	2.519153000	2.635152000
N	-0.013152000	-1.226863000	2.312748000
C	0.004289000	-1.140919000	0.922230000
C	-0.013507000	-2.470990000	0.385469000
C	-0.048600000	-3.320914000	1.480114000
C	-0.043611000	-2.519153000	2.635152000
B	0.000000000	0.000000000	3.273151000
N	0.000000000	0.000000000	0.265155000
F	-1.137111000	0.010273000	4.055533000
F	1.137111000	-0.010273000	4.055533000
H	0.050671000	4.400826000	1.472350000
H	-0.050671000	-4.400826000	1.472350000

C	-0.008831000	-2.849607000	-1.030316000
C	-0.547119000	-4.075199000	-1.434784000
C	0.536797000	-2.032156000	-2.024775000
C	-0.515782000	-4.418375000	-2.778387000
C	0.514604000	-2.470526000	-3.342064000
N	0.002846000	-3.640069000	-3.731326000
H	0.973203000	-1.073347000	-1.777930000
H	-0.933122000	-5.367924000	-3.104835000
H	0.936292000	-1.845180000	-4.125437000
H	-1.001648000	-4.748465000	-0.716849000
C	0.008831000	2.849607000	-1.030316000
C	0.547119000	4.075199000	-1.434784000
C	-0.536797000	2.032156000	-2.024775000
C	0.515782000	4.418375000	-2.778387000
C	-0.514604000	2.470526000	-3.342064000
N	-0.002846000	3.640069000	-3.731326000
H	1.001648000	4.748465000	-0.716849000
H	-0.973203000	1.073347000	-1.777930000
H	0.933122000	5.367924000	-3.104835000
H	-0.936292000	1.845180000	-4.125437000
H	0.055006000	2.834470000	3.669818000
H	-0.055006000	-2.834470000	3.669818000

38

D6 GS CAM-B3LYP/6-31G(d)

N	0.002519000	1.225461000	2.278724000
C	-0.022259000	1.142725000	0.887006000
C	-0.026119000	2.477293000	0.358119000
C	0.015247000	3.322952000	1.454822000
C	0.026361000	2.518117000	2.605355000

N	-0.002519000	-1.225461000	2.278724000
C	0.022259000	-1.142725000	0.887006000
C	0.026119000	-2.477293000	0.358119000
C	-0.015247000	-3.322952000	1.454822000
C	-0.026361000	-2.518117000	2.605355000
B	0.000000000	0.000000000	3.236681000
N	0.000000000	0.000000000	0.231509000
F	-1.136798000	-0.000031000	4.022418000
F	1.136798000	0.000031000	4.022418000
H	0.026436000	4.401207000	1.429937000
H	-0.026436000	-4.401207000	1.429937000
C	0.063841000	-2.905132000	-1.046102000
N	-0.239554000	-4.196754000	-1.259772000
C	0.417912000	-2.041817000	-2.086140000
C	-0.209705000	-4.651394000	-2.507911000
C	0.440985000	-2.532375000	-3.385164000
C	0.119390000	-3.863692000	-3.608208000
H	0.673410000	-1.012785000	-1.871619000
H	-0.462102000	-5.700814000	-2.641644000
H	0.712944000	-1.881267000	-4.209560000
H	0.125683000	-4.289889000	-4.605049000
C	-0.063841000	2.905132000	-1.046102000
N	0.239554000	4.196754000	-1.259772000
C	-0.417912000	2.041817000	-2.086140000
C	0.209705000	4.651394000	-2.507911000
C	-0.440985000	2.532375000	-3.385164000
C	-0.119390000	3.863692000	-3.608208000
H	-0.673410000	1.012785000	-1.871619000
H	0.462102000	5.700814000	-2.641644000

H	-0.712944000	1.881267000	-4.209560000
H	-0.125683000	4.289889000	-4.605049000
H	0.047230000	2.827760000	3.641690000
H	-0.047230000	-2.827760000	3.641690000

20

D7 GS CAM-B3LYP/6-31G(d)

N	0.000000000	1.226775000	-0.299725000
C	0.000000000	1.138624000	1.095436000
C	0.000000000	2.458437000	1.622128000
C	0.000000000	3.320377000	0.548299000
C	0.000000000	2.519280000	-0.616813000
N	0.000000000	-1.226775000	-0.299725000
C	0.000000000	-1.138624000	1.095436000
C	0.000000000	-2.458437000	1.622128000
C	0.000000000	-3.320377000	0.548299000
C	0.000000000	-2.519280000	-0.616813000
B	0.000000000	0.000000000	-1.259937000
N	0.000000000	0.000000000	1.759528000
F	1.137348000	0.000000000	-2.043805000
F	-1.137348000	0.000000000	-2.043805000
H	0.000000000	2.696868000	2.676126000
H	0.000000000	4.400235000	0.564141000
H	0.000000000	2.837940000	-1.650763000
H	0.000000000	-2.696868000	2.676126000
H	0.000000000	-4.400235000	0.564141000
H	0.000000000	-2.837940000	-1.650763000

54

A1 ES CAM-B3LYP/6-31G(d)

N	0.096982745	1.233816476	-0.719105700

C	0.044658353	1.142765588	0.651955467
C	0.000469275	2.474426858	1.221395982
C	0.000000000	3.332423165	0.139847135
C	0.064892766	2.575049213	-1.050539878
N	-0.096982745	-1.233816476	-0.719105700
C	-0.044658353	-1.142765588	0.651955467
C	-0.000469275	-2.474426858	1.221395982
C	0.000000000	-3.332423165	0.139847135
C	-0.064892766	-2.575049213	-1.050539878
B	0.000000000	0.000000000	-1.649282266
N	0.000000000	0.000000000	1.330695109
F	1.133247060	-0.123915830	-2.452901758
F	-1.133247060	0.123915830	-2.452901758
C	-0.045729177	2.828111468	2.635889886
H	0.012234320	4.412571278	0.175304487
C	0.100736466	3.113410407	-2.370380657
C	-0.100736466	-3.113410407	-2.370380657
H	-0.012234320	-4.412571278	0.175304487
C	0.045729177	-2.828111468	2.635889886
C	0.352278775	2.328078153	4.976935073
C	0.389280574	1.948810151	3.641352488
C	-0.550766432	4.469439766	4.354730151
C	-0.352278775	-2.328078153	4.976935073
C	-0.116293728	3.586631872	5.341738305
C	-0.516721458	4.095492318	3.020342010
C	-0.389280574	-1.948810151	3.641352488
C	0.116293728	-3.586631872	5.341738305
H	-0.145286565	3.878518756	6.386769814
H	-0.758266743	-0.969581257	3.366143048

H	0.145286565	-3.878518756	6.386769814
H	-0.924201885	5.451401030	4.627902512
H	-0.696779289	-1.634588432	5.737989775
H	-0.878023899	4.784962224	2.264353174
S	0.321782282	-4.799895781	-2.613587398
C	0.021405387	-4.686953290	-4.297469596
C	-0.380253712	-3.430799491	-4.666573240
C	-0.450888627	-2.533572644	-3.580578878
C	0.450888627	2.533572644	-3.580578878
C	0.380253712	3.430799491	-4.666573240
C	-0.021405387	4.686953290	-4.297469596
S	-0.321782282	4.799895781	-2.613587398
H	0.161164824	-5.552515372	-4.930729390
H	-0.624385883	-3.160575409	-5.686713293
H	-0.781958050	-1.509006235	-3.659858885
H	0.781958050	1.509006235	-3.659858885
H	0.624385883	3.160575409	-5.686713293
H	-0.161164824	5.552515372	-4.930729390
C	0.516721458	-4.095492318	3.020342010
C	0.550766432	-4.469439766	4.354730151
H	0.696779289	1.634588432	5.737989775
H	0.758266743	0.969581257	3.366143048
H	0.878023899	-4.784962224	2.264353174
H	0.924201885	-5.451401030	4.627902512

48

A2 ES CAM-B3LYP/6-31G(d)

N	0.821219044	-1.045069064	0.127054145
C	1.212070196	0.268833377	0.084761094
C	2.662110867	0.326482074	0.067740934

C	3.087759202	-0.993351476	0.066908892
C	1.964283352	-1.830751792	0.108803952
N	-1.487849671	-0.195086641	-0.089274003
C	-0.927621592	1.053745687	-0.032784629
C	-1.982095988	2.046830855	-0.032374686
C	-3.169509255	1.328664381	-0.061320744
C	-2.867042292	-0.039385293	-0.097245349
B	-0.656770430	-1.494248932	0.023276322
N	0.377795071	1.306741573	0.031067207
F	-1.058250621	-2.196234229	1.159183143
F	-0.808287905	-2.297801573	-1.105989923
H	4.112636682	-1.337171624	0.085165117
H	-4.168552974	1.738593008	-0.094099664
C	-1.833455576	3.472035284	-0.019112144
S	-0.290115531	4.285399724	-0.100206074
C	-2.855288043	4.401773718	0.038152254
C	-1.040178302	5.830841067	-0.055063591
C	-2.402633290	5.740311805	0.018840911
H	-3.900541541	4.122986262	0.094707543
H	-0.434277089	6.726181561	-0.081910227
H	-3.059516652	6.600694373	0.059127401
C	3.468793973	1.510993386	0.062452015
S	5.197418038	1.404215228	-0.183947563
C	3.095158142	2.825400885	0.253304094
C	5.379457742	3.108658311	-0.026062401
C	4.184895470	3.729580628	0.202090735
H	2.070012512	3.111950730	0.433902034
H	6.358802002	3.558423036	-0.113992834
H	4.085502399	4.800532826	0.331984779

C	1.996504156	-3.261178720	0.132769957
S	3.477473581	-4.081735128	-0.323916086
C	1.035750877	-4.186229622	0.500895737
C	2.784521350	-5.619574353	-0.011859330
C	1.489407135	-5.521532838	0.417399234
H	0.055908494	-3.899139036	0.851667522
H	3.367576791	-6.516913073	-0.167971831
H	0.880185715	-6.380139487	0.672277625
C	-3.826885625	-1.100399213	-0.132303524
S	-5.493962650	-0.746864672	0.281218004
C	-3.696806422	-2.433325624	-0.479569849
C	-5.968488219	-2.367482940	-0.020398010
C	-4.915083153	-3.145809877	-0.414795450
H	-2.760076853	-2.861665375	-0.802933409
H	-6.999996550	-2.662768010	0.115083529
H	-5.009878473	-4.197119793	-0.657812724

48

A3 ES CAM-B3LYP/6-31G(d)

N	0.074092093	1.234549635	-0.430180830
C	0.043922874	1.137383596	0.936465440
C	0.023501738	2.467943643	1.498045556
C	0.000000000	3.340256359	0.421683809
C	0.038223621	2.583432095	-0.759566370
N	-0.074092093	-1.234549635	-0.430180830
C	-0.043922874	-1.137383596	0.936465440
C	-0.023501738	-2.467943643	1.498045556
C	0.000000000	-3.340256359	0.421683809
C	-0.038223621	-2.583432095	-0.759566370
B	0.000000000	0.000000000	-1.363014244

N	0.000000000	0.000000000	1.627028150
F	1.136043714	-0.100119591	-2.164799926
F	-1.136043714	0.100119591	-2.164799926
H	0.001182532	4.419182834	0.474154537
H	-0.001182532	-4.419182834	0.474154537
C	-0.047852437	-2.783764827	2.886684336
O	0.110693165	-4.101076361	3.219682619
C	-0.230703645	-2.046210960	4.032138287
C	0.031627906	-4.188443726	4.567247131
C	-0.177520124	-2.961457224	5.116912250
H	-0.408021982	-0.983727107	4.066595637
H	0.144995259	-5.177601562	4.983308891
H	-0.282955774	-2.738971815	6.168556114
C	0.047852437	2.783764827	2.886684336
O	-0.110693165	4.101076361	3.219682619
C	0.230703645	2.046210960	4.032138287
C	-0.031627906	4.188443726	4.567247131
C	0.177520124	2.961457224	5.116912250
H	0.408021982	0.983727107	4.066595637
H	-0.144995259	5.177601562	4.983308891
H	0.282955774	2.738971815	6.168556114
C	0.041516694	3.116790278	-2.087811119
C	0.399004411	2.545130209	-3.295680404
C	0.292797000	3.437861975	-4.385964653
C	-0.142935295	4.680910499	-4.017882035
S	-0.433641703	4.787077785	-2.330164493
H	0.759218812	1.530481873	-3.375457341
H	0.536677146	3.170736766	-5.407025231
H	-0.314765798	5.540528290	-4.651192725

C	-0.041516694	-3.116790278	-2.087811119
C	-0.399004411	-2.545130209	-3.295680404
C	-0.292797000	-3.437861975	-4.385964653
C	0.142935295	-4.680910499	-4.017882035
S	0.433641703	-4.787077785	-2.330164493
H	-0.759218812	-1.530481873	-3.375457341
H	-0.536677146	-3.170736766	-5.407025231
H	0.314765798	-5.540528290	-4.651192725

52

A4 ES CAM-B3LYP/6-31G(d)

N	0.092549663	1.234358444	-0.713456447
C	0.042634592	1.141533721	0.657514892
C	0.000880539	2.470532283	1.227000181
C	0.000000000	3.332411857	0.150448987
C	0.061396922	2.574956337	-1.043404420
N	-0.092549663	-1.234358444	-0.713456447
C	-0.042634592	-1.141533721	0.657514892
C	-0.000880539	-2.470532283	1.227000181
C	0.000000000	-3.332411857	0.150448987
C	-0.061396922	-2.574956337	-1.043404420
B	0.000000000	0.000000000	-1.645032909
N	0.000000000	0.000000000	1.338441692
F	1.133520840	-0.119503932	-2.447187173
F	-1.133520840	0.119503932	-2.447187173
H	0.013596368	4.412496436	0.188392701
H	-0.013596368	-4.412496436	0.188392701
C	0.044548640	-2.810597199	2.643124009
C	0.549459311	-4.056445885	3.049323973
C	-0.410415506	-1.953750402	3.654210897

N	0.609929290	-4.467790596	4.311021317
C	-0.346001371	-2.372273466	4.974428259
C	0.164835879	-3.633492703	5.255746378
H	-0.809004337	-0.980309485	3.398822823
H	-0.694723689	-1.732438808	5.777674999
H	0.221314220	-3.991174168	6.280969872
H	0.940168559	-4.746425092	2.304759339
C	-0.044548640	2.810597199	2.643124009
C	-0.549459311	4.056445885	3.049323973
C	0.410415506	1.953750402	3.654210897
N	-0.609929290	4.467790596	4.311021317
C	0.346001371	2.372273466	4.974428259
C	-0.164835879	3.633492703	5.255746378
H	-0.940168559	4.746425092	2.304759339
H	0.809004337	0.980309485	3.398822823
H	0.694723689	1.732438808	5.777674999
H	-0.221314220	3.991174168	6.280969872
C	-0.095839471	-3.115916390	-2.360594413
C	-0.436930458	-2.535184899	-3.574267265
C	-0.367004751	-3.434120639	-4.657411145
C	0.025131520	-4.692744569	-4.283423580
S	0.316409142	-4.805915162	-2.599116311
H	-0.762236689	-1.509075320	-3.656969345
H	-0.604596342	-3.164789650	-5.679254805
H	0.162434255	-5.560025138	-4.914948361
C	0.095839471	3.115916390	-2.360594413
C	0.436930458	2.535184899	-3.574267265
C	0.367004751	3.434120639	-4.657411145
C	-0.025131520	4.692744569	-4.283423580

S	-0.316409142	4.805915162	-2.599116311
H	0.762236689	1.509075320	-3.656969345
H	0.604596342	3.164789650	-5.679254805
H	-0.162434255	5.560025138	-4.914948361

52

A5 ES CAM-B3LYP/6-31G(d)

N	0.091276640	1.234497839	-0.717591613
C	0.043696639	1.141824245	0.654265644
C	0.003453946	2.471794783	1.221902093
C	0.000000000	3.331944551	0.145337109
C	0.061056423	2.573261356	-1.049233038
N	-0.091276640	-1.234497839	-0.717591613
C	-0.043696639	-1.141824245	0.654265644
C	-0.003453946	-2.471794783	1.221902093
C	0.000000000	-3.331944551	0.145337109
C	-0.061056423	-2.573261356	-1.049233038
B	0.000000000	0.000000000	-1.649414671
N	0.000000000	0.000000000	1.333249807
F	1.133585955	-0.117985581	-2.450770148
F	-1.133585955	0.117985581	-2.450770148
H	0.010087884	4.412084292	0.182816608
H	-0.010087884	-4.412084292	0.182816608
C	0.030150444	-2.825900719	2.636982758
C	0.475984831	-4.091975200	3.041608897
C	-0.391126021	-1.954018325	3.649252300
C	0.479056708	-4.415206580	4.388822343
C	-0.344919535	-2.378915624	4.969869112
N	0.079334255	-3.584183977	5.356376407
H	-0.751520770	-0.963223510	3.408645374

H	0.826443008	-5.394995603	4.708630385
H	-0.671822475	-1.708080584	5.761375176
H	0.834645970	-4.814867333	2.317581256
C	-0.030150444	2.825900719	2.636982758
C	-0.475984831	4.091975200	3.041608897
C	0.391126021	1.954018325	3.649252300
C	-0.479056708	4.415206580	4.388822343
C	0.344919535	2.378915624	4.969869112
N	-0.079334255	3.584183977	5.356376407
H	-0.834645970	4.814867333	2.317581256
H	0.751520770	0.963223510	3.408645374
H	-0.826443008	5.394995603	4.708630385
H	0.671822475	1.708080584	5.761375176
C	-0.093938252	-3.114557807	-2.365548380
C	-0.429156350	-2.532279621	-3.580851272
C	-0.359116544	-3.431182383	-4.663047581
C	0.027429627	-4.691528086	-4.287014112
S	0.312317420	-4.806391490	-2.602606161
H	-0.750986613	-1.505204070	-3.664783726
H	-0.592377989	-3.161571829	-5.685755483
H	0.163824409	-5.559179513	-4.918286141
C	0.093938252	3.114557807	-2.365548380
C	0.429156350	2.532279621	-3.580851272
C	0.359116544	3.431182383	-4.663047581
C	-0.027429627	4.691528086	-4.287014112
S	-0.312317420	4.806391490	-2.602606161
H	0.750986613	1.505204070	-3.664783726
H	0.592377989	3.161571829	-5.685755483
H	-0.163824409	5.559179513	-4.918286141

52

A6 ES CAM-B3LYP/6-31G(d)

N	0.082652034	1.233786519	-0.694468361
C	0.048311187	1.143104510	0.678650613
C	0.023045958	2.476461565	1.239344002
C	-0.000004491	3.334241249	0.162659053
C	0.047870940	2.573558976	-1.028539371
N	-0.082652034	-1.233786519	-0.694468361
C	-0.048311187	-1.143104510	0.678650613
C	-0.023045958	-2.476461565	1.239344002
C	0.000004491	-3.334241249	0.162659053
C	-0.047870940	-2.573558976	-1.028539371
B	0.000000000	0.000000000	-1.624619902
N	0.000000000	0.000000000	1.358001417
F	1.134451202	-0.107373595	-2.428320581
F	-1.134451202	0.107373595	-2.428320581
H	-0.010069034	4.412390844	0.221334577
H	0.010069034	-4.412390844	0.221334577
C	-0.026473970	-2.875117204	2.644297528
N	0.245390708	-4.174513198	2.874289967
C	-0.321498401	-1.986086630	3.684849985
C	0.242542998	-4.605926998	4.130024420
C	-0.317780237	-2.455657904	4.991086791
C	-0.026612704	-3.792046801	5.228395873
H	-0.556702176	-0.955439921	3.458221991
H	0.468099733	-5.660466578	4.274021424
H	-0.545282875	-1.783163549	5.812129095
H	-0.011186157	-4.201708622	6.232069184
C	0.026473970	2.875117204	2.644297528

N	-0.245390708	4.174513198	2.874289967
C	0.321498401	1.986086630	3.684849985
C	-0.242542998	4.605926998	4.130024420
C	0.317780237	2.455657904	4.991086791
C	0.026612704	3.792046801	5.228395873
H	0.556702176	0.955439921	3.458221991
H	-0.468099733	5.660466578	4.274021424
H	0.545282875	1.783163549	5.812129095
H	0.011186157	4.201708622	6.232069184
C	0.065131767	3.111547296	-2.347377131
C	0.388862621	2.530311785	-3.565597770
C	0.305567895	3.429283462	-4.647677771
C	-0.079398533	4.688595830	-4.268162230
S	-0.345947316	4.802762821	-2.580084224
H	0.712637381	1.504179837	-3.652673336
H	0.528498632	3.159502201	-5.672710417
H	-0.224265606	5.555856438	-4.898046783
C	-0.065131767	-3.111547296	-2.347377131
C	-0.388862621	-2.530311785	-3.565597770
C	-0.305567895	-3.429283462	-4.647677771
C	0.079398533	-4.688595830	-4.268162230
S	0.345947316	-4.802762821	-2.580084224
H	-0.712637381	-1.504179837	-3.652673336
H	-0.528498632	-3.159502201	-5.672710417
H	0.224265606	-5.555856438	-4.898046783

34

A7 ES CAM-B3LYP/6-31G(d)

N	0.041853003	1.235943097	1.065523082
C	-0.015443001	1.138348085	2.445795188

C	-0.102662008	2.450298185	3.003049227
C	-0.114267009	3.325224254	1.954153148
C	-0.022494002	2.570413197	0.741138058
N	-0.041853003	-1.235943097	1.065523082
C	0.015443001	-1.138348085	2.445795188
C	0.102662008	-2.450298185	3.003049227
C	0.114267009	-3.325224254	1.954153148
C	0.022494002	-2.570413197	0.741138058
B	0.000000000	0.000000000	0.134492010
N	0.000000000	0.000000000	3.134790237
F	1.136702084	-0.070074005	-0.671817051
F	-1.136702084	0.070074005	-0.671817051
H	-0.158053012	4.404477337	2.005147155
C	0.000000000	3.124637236	-0.566969045
C	0.000000000	-3.124637236	-0.566969045
H	0.158053012	-4.404477337	2.005147155
S	0.412736031	-4.819977370	-0.785094061
C	0.146991011	-4.721641362	-2.474771188
C	-0.238024018	-3.465297265	-2.865836218
C	-0.323218025	-2.555909195	-1.793930136
C	0.323218025	2.555909195	-1.793930136
C	0.238024018	3.465297265	-2.865836218
C	-0.146991011	4.721641362	-2.474771188
S	-0.412736031	4.819977370	-0.785094061
H	0.291042022	-5.595060429	-3.096279236
H	-0.460431035	-3.205996243	-3.893786297
H	-0.649096047	-1.531396116	-1.891174145
H	0.649096047	1.531396116	-1.891174145
H	0.460431035	3.205996243	-3.893786297

H	-0.291042022	5.595060429	-3.096279236
H	-0.145120011	2.661212202	4.061520308
H	0.145120011	-2.661212202	4.061520308

52

B1 ES CAM-B3LYP/6-31G(d)

N	-0.019689002	1.218858641	-1.626965448
C	0.000000000	1.143650129	-0.260559874
C	-0.021814277	2.499736977	0.273869556
C	-0.068286530	3.336334547	-0.850789405
C	-0.058561261	2.545042609	-1.990884468
N	0.019689002	-1.218858641	-1.626965448
C	0.000000000	-1.143650129	-0.260559874
C	0.021814277	-2.499736977	0.273869556
C	0.068286530	-3.336334547	-0.850789405
C	0.058561261	-2.545042609	-1.990884468
B	0.000000000	0.000000000	-2.572249283
N	0.000000000	0.000000000	0.429988490
F	1.137107154	0.018209206	-3.378191854
F	-1.137107154	-0.018209206	-3.378191854
C	-0.006325114	2.891972261	1.662656723
H	-0.075947301	4.415898898	-0.853876964
H	0.075947301	-4.415898898	-0.853876964
C	0.006325114	-2.891972261	1.662656723
C	0.350221840	2.408401185	4.019555648
C	0.326756472	1.991396484	2.697442519
C	-0.289121941	4.630810664	3.342113602
C	-0.350221840	-2.408401185	4.019555648
C	0.041765887	3.725014533	4.351892629
C	-0.311288346	4.224228538	2.020761419

C	-0.326756472	-1.991396484	2.697442519
C	-0.041765887	-3.725014533	4.351892629
H	0.057342189	4.045926545	5.388602454
H	-0.577167095	-0.970059395	2.444909054
H	-0.057342189	-4.045926545	5.388602454
H	-0.534131867	5.658107188	3.592534638
H	-0.614734796	-1.699658532	4.798183639
H	-0.585118179	4.936418024	1.250269920
C	0.311288346	-4.224228538	2.020761419
C	0.289121941	-4.630810664	3.342113602
H	0.614734796	1.699658532	4.798183639
H	0.577167095	0.970059395	2.444909054
H	0.585118179	-4.936418024	1.250269920
H	0.534131867	-5.658107188	3.592534638
C	0.075026631	-2.942607406	-3.425984015
H	-0.799489825	-2.492291120	-3.913104172
H	0.938035409	-2.458951503	-3.901561647
C	0.105952354	-4.448551261	-3.658997834
H	-0.774121422	-4.936529537	-3.229622616
H	0.996557708	-4.902410649	-3.214174826
H	0.119439086	-4.663257781	-4.730470181
C	-0.075026631	2.942607406	-3.425984015
H	0.799489825	2.492291120	-3.913104172
H	-0.938035409	2.458951503	-3.901561647
C	-0.105952354	4.448551261	-3.658997834
H	0.774121422	4.936529537	-3.229622616
H	-0.996557708	4.902410649	-3.214174826
H	-0.119439086	4.663257781	-4.730470181

46

B2 ES CAM-B3LYP/6-31G(d)

N	-0.005461073	1.218925709	-1.565214861
C	0.019203295	1.138479718	-0.202066264
C	0.036187024	2.489105494	0.328614484
C	0.000000000	3.339419650	-0.795244532
C	-0.018075066	2.551580837	-1.929282474
N	0.005461073	-1.218925709	-1.565214861
C	-0.019203295	-1.138479718	-0.202066264
C	-0.036187024	-2.489105494	0.328614484
C	0.000000000	-3.339419650	-0.795244532
C	0.018075066	-2.551580837	-1.929282474
B	0.000000000	0.000000000	-2.511254059
N	0.000000000	0.000000000	0.495705264
F	1.137247925	0.005710124	-3.317580733
F	-1.137247925	-0.005710124	-3.317580733
H	0.007190119	4.419816587	-0.784562724
H	-0.007190119	-4.419816587	-0.784562724
C	-0.088003971	-2.838600428	1.702817572
S	0.081870896	-4.507175771	2.212557670
C	-0.286457019	-2.021489006	2.810526564
C	-0.112236625	-4.076951524	3.865088735
C	-0.299784879	-2.729686336	4.029770092
H	-0.442105596	-0.957002693	2.712300752
H	-0.074703071	-4.839643311	4.630936725
H	-0.445463355	-2.264483066	4.997115474
C	0.088003971	2.838600428	1.702817572
S	-0.081870896	4.507175771	2.212557670
C	0.286457019	2.021489006	2.810526564
C	0.112236625	4.076951524	3.865088735

C	0.299784879	2.729686336	4.029770092
H	0.442105596	0.957002693	2.712300752
H	0.074703071	4.839643311	4.630936725
H	0.445463355	2.264483066	4.997115474
C	0.040559150	-2.947518663	-3.366348009
H	-0.822955756	-2.484771379	-3.860365748
H	0.915974395	-2.480296910	-3.834401471
C	0.048021724	-4.454186761	-3.597001322
H	-0.843646280	-4.926483250	-3.173871289
H	0.926978636	-4.922184214	-3.143549068
H	0.066860651	-4.671600117	-4.667877048
C	-0.040559150	2.947518663	-3.366348009
H	0.822955756	2.484771379	-3.860365748
H	-0.915974395	2.480296910	-3.834401471
C	-0.048021724	4.454186761	-3.597001322
H	0.843646280	4.926483250	-3.173871289
H	-0.926978636	4.922184214	-3.143549068
H	-0.066860651	4.671600117	-4.667877048

46

B3 ES CAM-B3LYP/6-31G(d)

N	-0.024313530	1.219148360	-1.330088920
C	0.000201351	1.136521344	0.033477319
C	0.000000000	2.481679195	0.559735669
C	-0.044237073	3.340562462	-0.555453238
C	-0.052731578	2.553462840	-1.691280032
N	0.024313530	-1.219148360	-1.330088920
C	-0.000201351	-1.136521344	0.033477319
C	0.000000000	-2.481679195	0.559735669
C	0.044237073	-3.340562462	-0.555453238

C	0.052731578	-2.553462840	-1.691280032
B	0.000000000	0.000000000	-2.276107550
N	0.000000000	0.000000000	0.735955270
F	1.137024046	0.023487387	-3.082974757
F	-1.137024046	-0.023487387	-3.082974757
H	-0.055263341	4.419598021	-0.529773616
H	0.055263341	-4.419598021	-0.529773616
C	-0.053313597	-2.822024051	1.928821624
O	0.041679543	-4.150511993	2.250174774
C	-0.209928894	-2.089117864	3.092621505
C	-0.051055675	-4.247953337	3.593250625
C	-0.207776168	-3.016198726	4.159871022
H	-0.337716700	-1.019801163	3.136629033
H	0.012922562	-5.245425194	4.000652452
H	-0.310575150	-2.805504042	5.214211351
C	0.053313597	2.822024051	1.928821624
O	-0.041679543	4.150511993	2.250174774
C	0.209928894	2.089117864	3.092621505
C	0.051055675	4.247953337	3.593250625
C	0.207776168	3.016198726	4.159871022
H	0.337716700	1.019801163	3.136629033
H	-0.012922562	5.245425194	4.000652452
H	0.310575150	2.805504042	5.214211351
C	-0.082113508	2.951191566	-3.128143797
H	0.785886218	2.499848607	-3.624827044
H	-0.953053898	2.475859293	-3.596430351
C	-0.106724867	4.458265590	-3.355218761
H	0.779788524	4.939363673	-2.931154301
H	-0.990348538	4.915141214	-2.899482890

H	-0.128910965	4.678551236	-4.425493701
C	0.082113508	-2.951191566	-3.128143797
H	-0.785886218	-2.499848607	-3.624827044
H	0.953053898	-2.475859293	-3.596430351
C	0.106724867	-4.458265590	-3.355218761
H	-0.779788524	-4.939363673	-2.931154301
H	0.990348538	-4.915141214	-2.899482890
H	0.128910965	-4.678551236	-4.425493701

50

B4 ES CAM-B3LYP/6-31G(d)

N	0.018876242	1.219098967	-1.622278113
C	0.000000000	1.141900494	-0.254448816
C	0.020750508	2.496878986	0.281794662
C	0.064250619	3.335278145	-0.835212972
C	0.055749480	2.542583617	-1.981639647
N	-0.018876242	-1.219098967	-1.622278113
C	0.000000000	-1.141900494	-0.254448816
C	-0.020750508	-2.496878986	0.281794662
C	-0.064250619	-3.335278145	-0.835212972
C	-0.055749480	-2.542583617	-1.981639647
B	0.000000000	0.000000000	-2.570716405
N	0.000000000	0.000000000	0.437881175
F	1.137173412	-0.017238565	-3.373030386
F	-1.137173412	0.017238565	-3.373030386
H	0.068271876	4.414936234	-0.837183528
H	-0.068271876	-4.414936234	-0.837183528
C	-0.010748516	-2.872977031	1.675493632
C	-0.364390010	-4.185137564	2.055932253
C	0.352452425	-1.995773119	2.713328134

N	-0.368704537	-4.629647924	3.304446051
C	0.348183503	-2.454428948	4.019837969
C	-0.014548659	-3.772868412	4.270364274
H	0.641754443	-0.979317209	2.481315042
H	0.629691070	-1.801263766	4.838539997
H	-0.022583999	-4.161433730	5.285642675
H	-0.675920217	-4.898491202	1.297040875
C	0.010748516	2.872977031	1.675493632
C	0.364390010	4.185137564	2.055932253
C	-0.352452425	1.995773119	2.713328134
N	0.368704537	4.629647924	3.304446051
C	-0.348183503	2.454428948	4.019837969
C	0.014548659	3.772868412	4.270364274
H	0.675920217	4.898491202	1.297040875
H	-0.641754443	0.979317209	2.481315042
H	-0.629691070	1.801263766	4.838539997
H	0.022583999	4.161433730	5.285642675
C	-0.070830983	-2.945907753	-3.413719973
H	-0.934743434	-2.464051200	-3.890205201
H	0.802392521	-2.492973843	-3.901354278
C	-0.097962749	-4.452352918	-3.643461381
H	-0.987770619	-4.907633935	-3.198739657
H	0.783477747	-4.937326865	-3.213722146
H	-0.110624571	-4.667968871	-4.714637854
C	0.070830983	2.945907753	-3.413719973
H	0.934743434	2.464051200	-3.890205201
H	-0.802392521	2.492973843	-3.901354278
C	0.097962749	4.452352918	-3.643461381
H	0.987770619	4.907633935	-3.198739657

H	-0.783477747	4.937326865	-3.213722146
H	0.110624571	4.667968871	-4.714637854

50

B5 ES CAM-B3LYP/6-31G(d)

N	0.020181083	1.219245469	-1.634088253
C	0.000000000	1.141683383	-0.261499215
C	0.018341814	2.497159540	0.274584300
C	0.061534620	3.332126778	-0.837559387
C	0.055079153	2.536623690	-1.991176627
N	-0.020181083	-1.219245469	-1.634088253
C	0.000000000	-1.141683383	-0.261499215
C	-0.018341814	-2.497159540	0.274584300
C	-0.061534620	-3.332126778	-0.837559387
C	-0.055079153	-2.536623690	-1.991176627
B	0.000000000	0.000000000	-2.584448073
N	0.000000000	0.000000000	0.427892483
F	1.137155055	-0.018732777	-3.384054005
F	-1.137155055	0.018732777	-3.384054005
H	0.068003535	4.411880871	-0.839537980
H	-0.068003535	-4.411880871	-0.839537980
C	-0.002937817	-2.883319975	1.671020359
C	-0.329095085	-4.196785215	2.052787168
C	0.349684731	-2.000489702	2.705138947
C	-0.291711712	-4.547373970	3.390767439
C	0.355680732	-2.459039484	4.014633183
N	0.042084257	-3.705352957	4.375773652
H	0.623945623	-0.977924456	2.486023237
H	-0.547510706	-5.560893200	3.690954203
H	0.630836289	-1.780638135	4.819209221

H	-0.626353846	-4.934330330	1.316371757
C	0.002937817	2.883319975	1.671020359
C	0.329095085	4.196785215	2.052787168
C	-0.349684731	2.000489702	2.705138947
C	0.291711712	4.547373970	3.390767439
C	-0.355680732	2.459039484	4.014633183
N	-0.042084257	3.705352957	4.375773652
H	0.626353846	4.934330330	1.316371757
H	-0.623945623	0.977924456	2.486023237
H	0.547510706	5.560893200	3.690954203
H	-0.630836289	1.780638135	4.819209221
C	0.072856658	2.945001769	-3.420587296
H	0.937486340	2.462884899	-3.896116265
H	-0.799091741	2.491171788	-3.910305496
C	0.099696291	4.451793864	-3.647813578
H	0.988922511	4.906888827	-3.201944180
H	-0.782803231	4.935859969	-3.219450268
H	0.113817502	4.667812478	-4.718791250
C	-0.072856658	-2.945001769	-3.420587296
H	-0.937486340	-2.462884899	-3.896116265
H	0.799091741	-2.491171788	-3.910305496
C	-0.099696291	-4.451793864	-3.647813578
H	-0.988922511	-4.906888827	-3.201944180
H	0.782803231	-4.935859969	-3.219450268
H	-0.113817502	-4.667812478	-4.718791250

50

B6 ES CAM-B3LYP/6-31G(d)

N	0.006610654	1.218924428	-1.601708257
C	-0.021837068	1.142578407	-0.231302769

C	-0.040549783	2.500504840	0.296584220
C	0.000000000	3.336766546	-0.816000938
C	0.019779030	2.541140239	-1.962918266
N	-0.006610654	-1.218924428	-1.601708257
C	0.021837068	-1.142578407	-0.231302769
C	0.040549783	-2.500504840	0.296584220
C	0.000000000	-3.336766546	-0.816000938
C	-0.019779030	-2.541140239	-1.962918266
B	0.000000000	0.000000000	-2.549473278
N	0.000000000	0.000000000	0.459498301
F	1.137257694	-0.006865190	-3.352286442
F	-1.137257694	0.006865190	-3.352286442
H	0.000891315	4.415115223	-0.791775035
H	-0.000891315	-4.415115223	-0.791775035
C	0.093151583	-2.924443995	1.679630168
N	-0.059064482	-4.252064082	1.888230062
C	0.311791590	-2.031721849	2.741835103
C	-0.010661322	-4.701683579	3.133121186
C	0.360002319	-2.524669184	4.037863186
C	0.191620927	-3.885966047	4.248725486
H	0.452685162	-0.980286100	2.534178013
H	-0.139518649	-5.774532163	3.260672355
H	0.530422853	-1.850633680	4.871242628
H	0.218671954	-4.315675558	5.243715417
C	-0.093151583	2.924443995	1.679630168
N	0.059064482	4.252064082	1.888230062
C	-0.311791590	2.031721849	2.741835103
C	0.010661322	4.701683579	3.133121186
C	-0.360002319	2.524669184	4.037863186

C	-0.191620927	3.885966047	4.248725486
H	-0.452685162	0.980286100	2.534178013
H	0.139518649	5.774532163	3.260672355
H	-0.530422853	1.850633680	4.871242628
H	-0.218671954	4.315675558	5.243715417
C	-0.045269350	-2.942412934	-3.395060675
H	-0.920956149	-2.472330226	-3.861841523
H	0.815880201	-2.475664085	-3.891443222
C	-0.052120368	-4.448890326	-3.625877265
H	-0.930146868	-4.917537022	-3.171883356
H	0.840636558	-4.920594857	-3.205025573
H	-0.072569238	-4.664025564	-4.697071789
C	0.045269350	2.942412934	-3.395060675
H	0.920956149	2.472330226	-3.861841523
H	-0.815880201	2.475664085	-3.891443222
C	0.052120368	4.448890326	-3.625877265
H	0.930146868	4.917537022	-3.171883356
H	-0.840636558	4.920594857	-3.205025573
H	0.072569238	4.664025564	-4.697071789

32

B7 ES CAM-B3LYP/6-31G(d)

N	0.000000000	1.216881095	0.265733020
C	0.000000000	1.138127085	1.666381126
C	0.000000000	2.465941188	2.185057164
C	0.000000000	3.316287254	1.101500087
C	0.000000000	2.518795190	-0.081999006
N	0.000000000	-1.216881095	0.265733020
C	0.000000000	-1.138127085	1.666381126
C	0.000000000	-2.465941188	2.185057164

C	0.000000000	-3.316287254	1.101500087
C	0.000000000	-2.518795190	-0.081999006
B	0.000000000	0.000000000	-0.682806051
N	0.000000000	0.000000000	2.365040179
F	1.136685087	0.000000000	-1.488100115
F	-1.136685087	0.000000000	-1.488100115
H	0.000000000	4.396994337	1.115816083
H	0.000000000	-4.396994337	1.115816083
C	0.000000000	-2.948789223	-1.506163114
H	-0.868377066	-2.489558189	-1.998276154
H	0.868377066	-2.489558189	-1.998276154
C	0.000000000	-4.459492340	-1.711497132
H	-0.885889065	-4.922942377	-1.267480097
H	0.885889065	-4.922942377	-1.267480097
H	0.000000000	-4.692170359	-2.779220211
C	0.000000000	2.948789223	-1.506163114
H	0.868377066	2.489558189	-1.998276154
H	-0.868377066	2.489558189	-1.998276154
C	0.000000000	4.459492340	-1.711497132
H	0.885889065	4.922942377	-1.267480097
H	-0.885889065	4.922942377	-1.267480097
H	0.000000000	4.692170359	-2.779220211
H	0.000000000	-2.710217207	3.237389250
H	0.000000000	2.710217207	3.237389250

60

C1 ES CAM-B3LYP/6-31G(d)

N	-0.678961060	-1.240648091	0.166548839
C	0.692759420	-1.152414971	0.175027770
C	1.258782355	-2.482388038	0.048860552

C	0.170876328	-3.326068455	-0.089495782
C	-1.013872097	-2.569608223	-0.000881398
N	-0.664313693	1.239379140	0.137900815
C	0.706761582	1.136755510	0.118568071
C	1.284716681	2.460867448	-0.033456718
C	0.200155506	3.314766409	-0.139174221
C	-0.989097404	2.571304422	-0.030759864
B	-1.592302502	0.006002969	0.286611918
N	1.378327956	-0.011319688	0.182871397
F	-2.176734360	0.022701443	1.566680608
F	-2.570329767	0.000780786	-0.688547783
H	0.198788221	-4.402886863	-0.166777660
H	0.233750354	4.386787704	-0.262266526
C	-2.324228241	3.136710309	-0.089163153
C	-3.429637212	2.589472548	0.588437158
C	-2.513864777	4.324369231	-0.825812149
C	-4.669206934	3.206888425	0.521732919
C	-3.757007023	4.929542053	-0.893055782
C	-4.842965639	4.373382379	-0.218809631
H	-3.302811062	1.702192450	1.192012855
H	-1.682505955	4.752076642	-1.374870762
H	-5.507691625	2.776514257	1.060057189
H	-3.882823840	5.834929173	-1.477910166
H	-5.817606662	4.848259856	-0.269279569
C	-2.352784317	-3.123761633	-0.051987373
C	-2.553200257	-4.317853339	-0.775273595
C	-3.452456880	-2.558281147	0.620341995
C	-3.801837729	-4.912278720	-0.834945595
C	-4.697563236	-3.164786210	0.560967308

C	-4.882103520	-4.338072752	-0.166526647
H	-1.725031115	-4.759085589	-1.318610960
H	-3.316433117	-1.665312320	1.213741428
H	-3.936650771	-5.823263619	-1.408980258
H	-5.531989010	-2.720976925	1.094638430
H	-5.861147101	-4.804397354	-0.211224866
C	2.669192232	-2.837817542	0.044551329
C	3.079124410	-4.063152355	-0.512142607
C	3.650874297	-2.003932683	0.608590604
C	4.413097830	-4.437741250	-0.506867832
C	4.986002847	-2.384316217	0.611256890
C	5.375187123	-3.599401348	0.054655381
H	2.343070316	-4.715468324	-0.970312173
H	3.355629995	-1.060571428	1.049153582
H	4.706958926	-5.384887630	-0.948206799
H	5.727479204	-1.727851800	1.056109749
H	6.420337855	-3.892165549	0.056051881
C	2.696549902	2.815411325	-0.056661989
C	3.710051585	1.865021887	-0.276655110
C	3.078804999	4.157792859	0.129463155
C	5.044238719	2.247702511	-0.311256534
C	4.411603973	4.532947698	0.095225756
C	5.404289702	3.578954727	-0.125776411
H	3.439193867	0.828703764	-0.426265252
H	2.321692593	4.910295210	0.321903032
H	5.808874973	1.497233619	-0.487274935
H	4.680336913	5.573578108	0.247391626
H	6.448828758	3.872973058	-0.150441177

54

C2 ES CAM-B3LYP/6-31G(d)

N	0.223262842	1.285361706	0.158711556
C	-1.031285388	0.736135815	0.171468830
C	-2.008342190	1.802944178	0.048646192
C	-1.264961372	2.973296522	-0.090720238
C	0.090486759	2.656618468	-0.010280424
N	1.063689721	-1.040687217	0.160114560
C	-0.252614723	-1.409188489	0.141670782
C	-0.337541798	-2.846984061	-0.017988495
C	0.982410990	-3.287446692	-0.122229801
C	1.830049309	-2.187840700	-0.008459704
B	1.511259689	0.434729699	0.303641024
N	-1.288760087	-0.572509822	0.192705747
F	2.046379360	0.632158100	1.589934026
F	2.440331026	0.768667536	-0.661498010
H	-1.657384811	3.976208390	-0.177919515
H	1.313247987	-4.309678233	-0.228245664
C	-1.522517554	-3.638416147	-0.067442473
S	-3.136750442	-2.976406127	0.061893003
C	-1.587268042	-5.015519513	-0.220005366
C	-3.843736988	-4.533518527	-0.082892797
C	-2.903482189	-5.519734058	-0.228025053
H	-0.704802134	-5.635255516	-0.321890862
H	-4.919459465	-4.642963082	-0.057953462
H	-3.148754860	-6.569161759	-0.336719483
C	-3.427457673	1.656601898	0.070668147
S	-4.468461037	3.013818921	-0.302738041
C	-4.189122337	0.544789883	0.386446030
C	-5.881840207	2.072325577	-0.029603031

C	-5.580896100	0.787153301	0.328162033
H	-3.741322209	-0.399283393	0.658589450
H	-6.859973657	2.517077618	-0.151450588
H	-6.333152305	0.038372314	0.544898740
C	1.177360138	3.623945536	-0.083460704
C	2.383623900	3.487236287	0.623356443
C	0.983579080	4.781256237	-0.860496522
C	3.356694970	4.473034186	0.545680292
C	1.962562997	5.758848566	-0.937276193
C	3.155673064	5.609073783	-0.233725072
H	2.542009307	2.622294147	1.252095994
H	0.067439110	4.894797656	-1.429847643
H	4.279181997	4.355786373	1.105568819
H	1.797637547	6.637375670	-1.552713133
H	3.923339127	6.374131844	-0.292324645
C	3.285137061	-2.246267578	-0.058423463
C	4.117466959	-1.377756932	0.666768186
C	3.884044557	-3.259035001	-0.830493849
C	5.496468357	-1.520876396	0.612981880
C	5.262289237	-3.392136473	-0.883332219
C	6.076345285	-2.522457176	-0.160847281
H	3.679349536	-0.610729570	1.289961722
H	3.259003314	-3.925455629	-1.414888765
H	6.124431357	-0.846663082	1.186827611
H	5.703758886	-4.172240530	-1.495155566
H	7.156044676	-2.625759666	-0.200959114

54

C3 ES CAM-B3LYP/6-31G(d)

N	-0.325735118	1.249853828	0.132823719

C	-1.441323529	0.456913827	0.161953318
C	-2.610408340	1.298614833	0.050988307
C	-2.135052497	2.597530562	-0.104515191
C	-0.740734831	2.564533583	-0.040103365
N	0.961102536	-0.867511803	0.168221503
C	-0.257864514	-1.488838401	0.135668557
C	-0.049220181	-2.910134688	-0.016429903
C	1.329296779	-3.091070915	-0.087250954
C	1.942299006	-1.842668128	0.025537906
B	1.105598173	0.672640815	0.287006174
N	-1.440756812	-0.875353766	0.184542737
F	1.588120727	0.994344477	1.569054978
F	1.952201230	1.166906871	-0.685052657
H	-2.735120526	3.490131395	-0.196189257
H	1.844386383	-4.034981662	-0.180986681
C	-1.070418702	-3.891872274	-0.096664231
O	-0.667202143	-5.198496268	-0.161902584
C	-2.447382053	-3.841930716	-0.149973561
C	-1.779802188	-5.959501413	-0.253288948
C	-2.898444279	-5.181427990	-0.252192466
H	-3.035997507	-2.939438995	-0.144185891
H	-1.619848459	-7.025388115	-0.307946604
H	-3.919566903	-5.527152647	-0.317931093
C	-3.956122758	0.851975741	0.105992926
O	-4.928434835	1.778771685	-0.156290105
C	-4.552407752	-0.353527252	0.409061866
C	-6.122041963	1.159528574	-0.022850156
C	-5.952085894	-0.147202885	0.323523661
H	-4.027772082	-1.253654199	0.685552862

H	-6.993430889	1.768959341	-0.207086911
H	-6.734475674	-0.871109399	0.497666144
C	0.125263419	3.732412647	-0.133482695
C	1.344817320	3.847618948	0.554060894
C	-0.310346699	4.821231224	-0.911577382
C	2.097376315	5.009107012	0.456416163
C	0.449384877	5.976058840	-1.007972199
C	1.659034108	6.075534426	-0.323971567
H	1.683678057	3.037467191	1.184443011
H	-1.239410673	4.743315927	-1.465816286
H	3.033023610	5.084534027	1.001508807
H	0.100653871	6.798903110	-1.623724661
H	2.255283346	6.979437061	-0.398253521
C	3.381195929	-1.614574440	-0.006891831
C	4.017235983	-0.583046710	0.703419013
C	4.178894024	-2.507417080	-0.746855633
C	5.398252531	-0.453800394	0.666493818
C	5.557109636	-2.369008548	-0.782585464
C	6.174697851	-1.339849071	-0.075115299
H	3.428803048	0.097434749	1.302625383
H	3.705572222	-3.297142024	-1.319971062
H	5.873237902	0.343987888	1.228809126
H	6.151509939	-3.061792284	-1.369604115
H	7.254197848	-1.230234766	-0.101908691

58

? ES CAM-B3LYP/6-31G(d)

N	-0.676858665	-1.240095863	0.169506147
C	0.695804396	-1.151721040	0.179808208
C	1.261021113	-2.479709126	0.059514739

C	0.178131580	-3.326079113	-0.077074288
C	-1.010925997	-2.567667445	0.005844416
N	-0.659570979	1.241299884	0.139312519
C	0.712396637	1.134555302	0.120543584
C	1.292817083	2.453465078	-0.036652466
C	0.216374955	3.312303534	-0.147243277
C	-0.979837580	2.571323726	-0.034394935
B	-1.590722294	0.008317694	0.286199873
N	1.384359281	-0.012695969	0.187146818
F	-2.177449331	0.026243299	1.563629620
F	-2.563560595	0.003476241	-0.692742899
H	0.207523761	-4.403134783	-0.150199001
H	0.255886522	4.383358625	-0.277237865
C	2.708312677	2.791361257	-0.059371930
C	3.115774163	4.115323442	0.182613974
C	3.722948080	1.860290498	-0.324993663
N	4.376828302	4.530044796	0.163130677
C	5.042589194	2.284333340	-0.343641072
C	5.323414855	3.622876153	-0.099623475
H	3.468493543	0.826395625	-0.517585875
H	5.846366810	1.586423750	-0.551358465
H	6.348470769	3.985148718	-0.113187906
C	2.673854146	-2.822812721	0.055619167
C	3.104207872	-4.027119289	-0.526974313
C	3.662394851	-2.011010153	0.629868075
N	4.366657854	-4.437920090	-0.556961584
C	4.983436422	-2.429357726	0.598192546
C	5.288707194	-3.646596968	0.000824693
H	3.388518123	-1.072971273	1.095895560

H	5.768793288	-1.824727167	1.038351762
H	6.315124083	-4.003536230	-0.033293518
C	-2.346403964	-3.124660118	-0.046110756
C	-3.452463457	-2.551790979	0.611026385
C	-2.539707454	-4.328751882	-0.756392609
C	-4.695958903	-3.160240652	0.548942845
C	-3.787135396	-4.924099598	-0.819454704
C	-4.873153598	-4.342230651	-0.166577153
H	-3.321831301	-1.653193076	1.196875978
H	-1.707410672	-4.776267562	-1.287945874
H	-5.535049083	-2.711678164	1.071011222
H	-3.916977940	-5.841941671	-1.383435496
H	-5.851350654	-4.810026753	-0.213523431
C	-2.309695853	3.143064262	-0.092719390
C	-3.421424322	2.593274210	0.574295139
C	-2.490982676	4.338874688	-0.820112280
C	-4.658018752	3.215228910	0.505549303
C	-3.731616177	4.947519284	-0.890017230
C	-4.823234196	4.388376966	-0.226822169
H	-3.300770375	1.702065183	1.173146707
H	-1.655723983	4.769369541	-1.360750413
H	-5.501035580	2.783849797	1.035732700
H	-3.851588418	5.858178681	-1.467678064
H	-5.796000619	4.866816504	-0.278985461
H	2.379230696	-4.679592789	-1.008196448
H	2.372359859	4.872030281	0.421637832

58

C5 ES CAM-B3LYP/6-31G(d)

N	-0.677245102	-1.242638581	0.164001539

C	0.696911906	-1.148661734	0.175257175
C	1.265969947	-2.473929029	0.048620299
C	0.187726357	-3.322183517	-0.094128928
C	-1.006885057	-2.567953460	-0.005718508
N	-0.670177744	1.239306965	0.144079982
C	0.704074262	1.138170223	0.123782028
C	1.277282238	2.459902874	-0.030990445
C	0.198455529	3.312763939	-0.140490182
C	-0.997155699	2.565167431	-0.028284679
B	-1.596501291	0.001709109	0.287867070
N	1.378574881	-0.006646440	0.186196621
F	-2.180130000	0.011647037	1.565813318
F	-2.569609139	-0.002700374	-0.689996930
H	0.222007739	-4.398444579	-0.176578160
H	0.233800926	4.384467690	-0.266410451
C	2.691119739	2.815611360	-0.061410004
C	3.710750422	1.883584436	-0.298699937
C	3.090880099	4.146209322	0.135175174
C	5.029422037	2.315966810	-0.327953381
C	4.436544891	4.469612865	0.084901780
N	5.410045193	3.581655034	-0.141603593
H	2.364353296	4.923224153	0.343393586
H	5.824867662	1.597593968	-0.514142089
H	4.750542610	5.499381372	0.239604947
H	3.476173408	0.841105700	-0.462772063
C	2.679773269	-2.824808571	0.051000311
C	3.665255128	-1.999029496	0.608650438
C	3.112695574	-4.042047739	-0.494444820
C	4.988404603	-2.417581431	0.589788174

C	4.459746985	-4.363514922	-0.459242390
N	5.401411031	-3.575479059	0.069197025
H	3.402077581	-1.049052425	1.053970328
H	2.410795606	-4.725673867	-0.958481484
H	5.758932970	-1.783017887	1.022056871
H	4.802103382	-5.304551856	-0.883635448
C	-2.338312706	-3.131572046	-0.058721394
C	-3.448168374	-2.563830730	0.597490127
C	-2.524776523	-4.336661907	-0.770121456
C	-4.688454418	-3.177824116	0.533001068
C	-3.769390839	-4.936770075	-0.836222828
C	-4.858890038	-4.359814052	-0.184426449
H	-3.322278980	-1.666044050	1.185511182
H	-1.689923322	-4.780531090	-1.300581013
H	-5.530364012	-2.734084630	1.054503395
H	-3.894621545	-5.854605359	-1.401119605
H	-5.834881071	-4.831946581	-0.233296218
C	-2.327355726	3.133378403	-0.080840402
C	-3.437064288	2.575542528	0.583938500
C	-2.512635567	4.333582207	-0.800961171
C	-4.675351434	3.193804703	0.519589701
C	-3.755269300	4.937766389	-0.867090287
C	-4.844477260	4.370571993	-0.206386747
H	-3.312852486	1.681944969	1.178419207
H	-1.679260908	4.770493656	-1.339242803
H	-5.516674408	2.757145291	1.047990218
H	-3.879029825	5.851328164	-1.439208270
H	-5.818822129	4.846100395	-0.255130592

58

C6 ES CAM-B3LYP/6-31G(d)

N	-0.606759693	-1.262909974	0.143464243
C	0.763491749	-1.123342026	0.173969076
C	1.372900640	-2.431215281	0.052087792
C	0.326768719	-3.311931591	-0.122558145
C	-0.890080853	-2.598615265	-0.044912865
N	-0.688978285	1.215870171	0.172826549
C	0.687561079	1.164944618	0.135032522
C	1.204624625	2.508414230	-0.028452111
C	0.095685196	3.323521631	-0.110958952
C	-1.067014050	2.533118166	0.014858741
B	-1.568445689	-0.056116629	0.290721805
N	1.403910517	0.043464569	0.191503856
F	-2.155125846	-0.096984686	1.568333974
F	-2.542309366	-0.073808375	-0.687796007
H	0.420820956	-4.380922171	-0.233428940
H	0.117745499	4.396865371	-0.215116682
C	2.592132367	2.951987803	-0.102570203
N	2.767481858	4.289167974	-0.104238368
C	3.674058219	2.066077566	-0.186240941
C	4.004658419	4.762797355	-0.178254954
C	4.960664296	2.581398896	-0.263043522
C	5.140038005	3.957622737	-0.255913981
H	3.492288104	1.000745818	-0.202691444
H	4.103360533	5.846317861	-0.175407622
H	5.811928643	1.911390509	-0.330529274
H	6.126565158	4.403788036	-0.311958229
C	2.788307914	-2.777046446	0.090981618
N	3.091778033	-4.023568706	-0.322825925

C	3.769801116	-1.894745927	0.561239850
C	4.362861092	-4.404753075	-0.291514788
C	5.093453881	-2.311459569	0.584719328
C	5.406325447	-3.590891352	0.146246702
H	3.485075609	-0.910890695	0.908337087
H	4.567549799	-5.416546294	-0.635097521
H	5.868696291	-1.643823363	0.947014573
H	6.426309726	-3.958353097	0.144930189
C	-2.203218744	-3.203443153	-0.126514751
C	-3.340213104	-2.685863348	0.523699302
C	-2.339254520	-4.400669235	-0.861296786
C	-4.558549987	-3.339475325	0.429803804
C	-3.562179375	-5.040832749	-0.956722795
C	-4.679697905	-4.512698635	-0.311406861
H	-3.252613563	-1.795643353	1.129883323
H	-1.481305161	-4.806944470	-1.384979434
H	-5.422019786	-2.933472489	0.946942875
H	-3.648055666	-5.952080390	-1.539499170
H	-5.638682838	-5.015781403	-0.383471863
C	-2.419124242	3.051510370	-0.009831538
C	-3.499473707	2.442500070	0.657645525
C	-2.657158302	4.256901476	-0.704816184
C	-4.760244181	3.016616094	0.619861021
C	-3.921366127	4.817581594	-0.744079702
C	-4.981240019	4.199616107	-0.081387516
H	-3.336118913	1.542216971	1.232214868
H	-1.845663049	4.734028031	-1.242300288
H	-5.578507129	2.539619966	1.149776075
H	-4.084690996	5.736967510	-1.296773768

H	-5.972671048	4.640315762	-0.109420045

40

C7 ES CAM-B3LYP/6-31G(d)

N	1.172113091	0.128577010	1.240820095
C	2.558353195	0.111529009	1.139642087
C	3.117988240	-0.031872002	2.444086189
C	2.067068159	-0.145519011	3.311004250
C	0.850542063	-0.034712003	2.562375196
N	1.172113091	0.128577010	-1.240820095
C	2.558353195	0.111529009	-1.139642087
C	3.117988240	-0.031872002	-2.444086189
C	2.067068159	-0.145519011	-3.311004250
C	0.850542063	-0.034712003	-2.562375196
B	0.251427019	0.262218020	0.000000000
N	3.246559246	0.143255011	0.000000000
F	-0.339046026	1.539901117	0.000000000
F	-0.725164055	-0.715521053	0.000000000
H	2.118552163	-0.251909019	4.384819334
H	2.118552163	-0.251909019	-4.384819334
C	-0.470141036	-0.068484005	-3.145300242
C	-1.592030124	0.566574041	-2.572740196
C	-0.643822050	-0.744247059	-4.374789335
C	-2.825324218	0.513627039	-3.201197246
C	-1.881444141	-0.797868061	-4.989420380
C	-2.981360226	-0.169480013	-4.405478334
H	-1.478615115	1.131700084	-1.659003125
H	0.196113015	-1.258408094	-4.827720371
H	-3.674078280	1.018467080	-2.750887209
H	-1.993522152	-1.335526100	-5.925304445

H	-3.952150304	-0.209059016	-4.889151372
C	-0.470141036	-0.068484005	3.145300242
C	-0.643822050	-0.744247059	4.374789335
C	-1.592030124	0.566574041	2.572740196
C	-1.881444141	-0.797868061	4.989420380
C	-2.825324218	0.513627039	3.201197246
C	-2.981360226	-0.169480013	4.405478334
H	0.196113015	-1.258408094	4.827720371
H	-1.478615115	1.131700084	1.659003125
H	-1.993522152	-1.335526100	5.925304445
H	-3.674078280	1.018467080	2.750887209
H	-3.952150304	-0.209059016	4.889151372
H	4.177348318	-0.059231005	2.653270204
H	4.177348318	-0.059231005	-2.653270204

40

D1 ES CAM-B3LYP/6-31G(d)

N	0.005906637	1.214351702	2.272842516
C	-0.011625314	1.144030628	0.909308699
C	0.000000000	2.503146085	0.381382306
C	0.040388980	3.340826584	1.516372452
C	0.035626727	2.535688168	2.632512084
N	-0.005906637	-1.214351702	2.272842516
C	0.011625314	-1.144030628	0.909308699
C	0.000000000	-2.503146085	0.381382306
C	-0.040388980	-3.340826584	1.516372452
C	-0.035626727	-2.535688168	2.632512084
B	0.000000000	0.000000000	3.225716959
N	0.000000000	0.000000000	0.216320088
F	-1.135051353	0.003002671	4.023918883

F	1.135051353	-0.003002671	4.023918883
C	-0.023277170	2.903046830	-1.000040430
H	0.041445421	4.420403514	1.529433716
H	-0.041445421	-4.420403514	1.529433716
C	0.023277170	-2.903046830	-1.000040430
C	-0.356155354	2.420061637	-3.361409807
C	-0.326569519	1.997102412	-2.042659149
C	0.216965290	4.659129156	-2.668507302
C	0.356155354	-2.420061637	-3.361409807
C	-0.083005670	3.747453653	-3.684172416
C	0.244089362	4.247991560	-1.350224379
C	0.326569519	-1.997102412	-2.042659149
C	0.083005670	-3.747453653	-3.684172416
H	-0.102840103	4.073271763	-4.719222826
H	0.550566408	-0.968740448	-1.794649193
H	0.102840103	-4.073271763	-4.719222826
H	0.432652392	5.693917036	-2.914172236
H	0.597127566	-1.709429399	-4.145657829
H	0.491607089	4.964059978	-0.574796974
C	-0.244089362	-4.247991560	-1.350224379
C	-0.216965290	-4.659129156	-2.668507302
H	-0.597127566	1.709429399	-4.145657829
H	-0.550566408	0.968740448	-1.794649193
H	-0.491607089	-4.964059978	-0.574796974
H	-0.432652392	-5.693917036	-2.914172236
H	0.044998481	2.814629471	3.677112715
H	-0.044998481	-2.814629471	3.677112715

34

D2 ES CAM-B3LYP/6-31G(d)

N	0.002447709	1.214430623	2.189907063
C	-0.019671317	1.138722028	0.827289062
C	-0.034369163	2.490823320	0.302081692
C	0.000000000	3.341730308	1.433556641
C	0.015555043	2.539786932	2.547695194
N	-0.002447709	-1.214430623	2.189907063
C	0.019671317	-1.138722028	0.827289062
C	0.034369163	-2.490823320	0.302081692
C	0.000000000	-3.341730308	1.433556641
C	-0.015555043	-2.539786932	2.547695194
B	0.000000000	0.000000000	3.143373418
N	0.000000000	0.000000000	0.127656721
F	-1.134915434	0.001079409	3.942538434
F	1.134915434	-0.001079409	3.942538434
H	-0.005829002	4.422351368	1.431763381
H	0.005829002	-4.422351368	1.431763381
C	0.085008332	-2.847089902	-1.068103398
S	-0.078194770	-4.518586648	-1.572170033
C	0.277198992	-2.031092351	-2.180708047
C	0.109118909	-4.091347871	-3.224220520
C	0.290490066	-2.742179041	-3.395513735
H	0.428030423	-0.965716279	-2.084956170
H	0.072999914	-4.856075550	-3.988248897
H	0.431482170	-2.281006011	-4.365331868
C	-0.085008332	2.847089902	-1.068103398
S	0.078194770	4.518586648	-1.572170033
C	-0.277198992	2.031092351	-2.180708047
C	-0.109118909	4.091347871	-3.224220520
C	-0.290490066	2.742179041	-3.395513735

H	-0.428030423	0.965716279	-2.084956170
H	-0.072999914	4.856075550	-3.988248897
H	-0.431482170	2.281006011	-4.365331868
H	0.030525791	2.819906628	3.591605318
H	-0.030525791	-2.819906628	3.591605318

34

D3 ES CAM-B3LYP/6-31G(d)

N	-0.020155140	1.214972522	-1.970975386
C	0.001419366	1.136743916	-0.607723240
C	0.000000000	2.483282417	-0.086649625
C	-0.040927003	3.342782281	-1.208511458
C	-0.047289455	2.541794778	-2.325360484
N	0.020155140	-1.214972522	-1.970975386
C	-0.001419366	-1.136743916	-0.607723240
C	0.000000000	-2.483282417	-0.086649625
C	0.040927003	-3.342782281	-1.208511458
C	0.047289455	-2.541794778	-2.325360484
B	0.000000000	0.000000000	-2.924437079
N	0.000000000	0.000000000	0.096417741
F	1.134826881	0.018226159	-3.723953347
F	-1.134826881	-0.018226159	-3.723953347
H	-0.051870599	4.422143581	-1.191166631
H	0.051870599	-4.422143581	-1.191166631
C	-0.050499707	-2.830191383	1.278980415
O	0.041561976	-4.159807204	1.595753151
C	-0.201026810	-2.100002510	2.447827716
C	-0.046530944	-4.260772815	2.937447161
C	-0.197760730	-3.029364069	3.510144121
H	-0.324748696	-1.030339569	2.495172294

H	0.015682973	-5.259582315	3.342056408
H	-0.296182659	-2.823036341	4.565689127
C	0.050499707	2.830191383	1.278980415
O	-0.041561976	4.159807204	1.595753151
C	0.201026810	2.100002510	2.447827716
C	0.046530944	4.260772815	2.937447161
C	0.197760730	3.029364069	3.510144121
H	0.324748696	1.030339569	2.495172294
H	-0.015682973	5.259582315	3.342056408
H	0.296182659	2.823036341	4.565689127
H	-0.066642159	2.823846823	-3.368665584
H	0.066642159	-2.823846823	-3.368665584

38

D4 ES CAM-B3LYP/6-31G(d)

N	0.006666001	1.214841095	2.264797175
C	-0.011222001	1.142282089	0.900984069
C	0.000000000	2.502192190	0.370698028
C	0.040111003	3.340969256	1.498382113
C	0.036503003	2.534616192	2.619067198
N	-0.006666001	-1.214841095	2.264797175
C	0.011222001	-1.142282089	0.900984069
C	0.000000000	-2.502192190	0.370698028
C	-0.040111003	-3.340969256	1.498382113
C	-0.036503003	-2.534616192	2.619067198
B	0.000000000	0.000000000	3.221469248
N	0.000000000	0.000000000	0.206069015
F	-1.135356085	0.003680000	4.015031309
F	1.135356085	-0.003680000	4.015031309
H	0.038735003	4.420574337	1.511205115

H	-0.038735003	-4.420574337	1.511205115
C	0.020773002	-2.885496222	-1.015139079
C	-0.286353022	-4.215054324	-1.387075108
C	0.349612027	-2.000982154	-2.061210156
N	-0.280247022	-4.664973358	-2.631851200
C	0.356630027	-2.467558190	-3.363615259
C	0.039165003	-3.800216292	-3.604116275
H	0.606124045	-0.974739074	-1.834837141
H	0.612297046	-1.811088137	-4.187933318
H	0.041034003	-4.194956321	-4.616902351
H	-0.567133043	-4.934991378	-0.622803045
C	-0.020773002	2.885496222	-1.015139079
C	0.286353022	4.215054324	-1.387075108
C	-0.349612027	2.000982154	-2.061210156
N	0.280247022	4.664973358	-2.631851200
C	-0.356630027	2.467558190	-3.363615259
C	-0.039165003	3.800216292	-3.604116275
H	0.567133043	4.934991378	-0.622803045
H	-0.606124045	0.974739074	-1.834837141
H	-0.612297046	1.811088137	-4.187933318
H	-0.041034003	4.194956321	-4.616902351
H	0.045808004	2.818093217	3.662597282
H	-0.045808004	-2.818093217	3.662597282

38

D5 ES CAM-B3LYP/6-31G(d)

N	0.007572001	1.215327092	2.282055174
C	-0.010509001	1.141837088	0.911807072
C	0.000000000	2.504144193	0.380029029
C	0.039178003	3.337578256	1.499635115

C	0.036541003	2.527752192	2.629619199
N	-0.007572001	-1.215327092	2.282055174
C	0.010509001	-1.141837088	0.911807072
C	0.000000000	-2.504144193	0.380029029
C	-0.039178003	-3.337578256	1.499635115
C	-0.036541003	-2.527752192	2.629619199
B	0.000000000	0.000000000	3.242473246
N	0.000000000	0.000000000	0.220173017
F	-1.135498084	0.004726000	4.031664308
F	1.135498084	-0.004726000	4.031664308
H	0.040641003	4.417303339	1.513065115
H	-0.040641003	-4.417303339	1.513065115
C	0.021205002	-2.894055219	-1.011463075
C	-0.275232021	-4.217503320	-1.388101103
C	0.351041027	-2.005220155	-2.049706155
C	-0.234036018	-4.570197347	-2.724711207
C	0.364431028	-2.467863187	-3.357669254
N	0.077680006	-3.722101285	-3.712488283
H	0.604047045	-0.976635074	-1.833978138
H	-0.467015036	-5.589718437	-3.022392233
H	0.622054048	-1.786655134	-4.165428320
H	-0.551956044	-4.959565380	-0.648462051
C	-0.021205002	2.894055219	-1.011463075
C	0.275232021	4.217503320	-1.388101103
C	-0.351041027	2.005220155	-2.049706155
C	0.234036018	4.570197347	-2.724711207
C	-0.364431028	2.467863187	-3.357669254
N	-0.077680006	3.722101285	-3.712488283
H	0.551956044	4.959565380	-0.648462051

H	-0.604047045	0.976635074	-1.833978138
H	0.467015036	5.589718437	-3.022392233
H	-0.622054048	1.786655134	-4.165428320
H	0.047987004	2.817537216	3.671805279
H	-0.047987004	-2.817537216	3.671805279

38

D6 ES CAM-B3LYP/6-31G(d)

N	0.018435001	1.214548095	2.241958170
C	-0.005774000	1.143019085	0.875855066
C	0.000000000	2.506255192	0.353693027
C	0.049733004	3.342108257	1.475982114
C	0.052548004	2.532852196	2.597398199
N	-0.018435001	-1.214548095	2.241958170
C	0.005774000	-1.143019085	0.875855066
C	0.000000000	-2.506255192	0.353693027
C	-0.049733004	-3.342108257	1.475982114
C	-0.052548004	-2.532852196	2.597398199
B	0.000000000	0.000000000	3.198338244
N	0.000000000	0.000000000	0.182134014
F	-1.135210085	0.015759001	3.992114304
F	1.135210085	-0.015759001	3.992114304
H	0.067420005	4.420459337	1.461163114
H	-0.067420005	-4.420459337	1.461163114
C	0.042264003	-2.935740226	-1.022152080
N	-0.117379009	-4.265464326	-1.225035092
C	0.257292020	-2.046265159	-2.090467160
C	-0.080663006	-4.717785358	-2.467029187
C	0.294209023	-2.543488193	-3.385249258
C	0.117658009	-3.904185297	-3.588278275

H	0.404664031	-0.995197077	-1.886080142
H	-0.215114016	-5.790061451	-2.592264196
H	0.461639035	-1.873737146	-4.222424324
H	0.135287010	-4.338221329	-4.581568350
C	-0.042264003	2.935740226	-1.022152080
N	0.117379009	4.265464326	-1.225035092
C	-0.257292020	2.046265159	-2.090467160
C	0.080663006	4.717785358	-2.467029187
C	-0.294209023	2.543488193	-3.385249258
C	-0.117658009	3.904185297	-3.588278275
H	-0.404664031	0.995197077	-1.886080142
H	0.215114016	5.790061451	-2.592264196
H	-0.461639035	1.873737146	-4.222424324
H	-0.135287010	4.338221329	-4.581568350
H	0.073448006	2.813064213	3.641855280
H	-0.073448006	-2.813064213	3.641855280

20

D7 ES CAM-B3LYP/6-31G(d)

N	0.000000000	1.212355772	-0.299237700
C	0.000000000	1.137737271	1.111959598
C	0.000000000	2.464597475	1.624562539
C	0.000000000	3.314333378	0.537476913
C	0.000000000	2.504135821	-0.631563871
N	0.000000000	-1.212355772	-0.299237700
C	0.000000000	-1.137737271	1.111959598
C	0.000000000	-2.464597475	1.624562539
C	0.000000000	-3.314333378	0.537476913
C	0.000000000	-2.504135821	-0.631563871
B	0.000000000	0.000000000	-1.261076719

N	0.000000000	0.000000000	1.810390221
F	1.134437126	0.000000000	-2.054930616
F	-1.134437126	0.000000000	-2.054930616
H	0.000000000	2.711717424	2.676017555
H	0.000000000	4.395275929	0.542847347
H	0.000000000	2.815058761	-1.668110509
H	0.000000000	-2.711717424	2.676017555
H	0.000000000	-4.395275929	0.542847347
H	0.000000000	-2.815058761	-1.668110509
